# Dysfunction of hippocampal cells and its role in cognitive impairment

**DOI:** 10.4103/NRR.NRR-D-25-00410

**Published:** 2025-09-29

**Authors:** Jingwen Ye, Lihong Zhou, Qiaohuizi Li, Yuchen Huang, Xiaoqin Wu, Liusuyu Zhu, Jie Zhu, Jiahao Liu, Dengsiyuan Gao, Xia Chen, Gang Chen, Ying Chen

**Affiliations:** 1 Department of Histology and Embryology, Medical School of Nantong University, Nantong, Jiangsu Province, China; 2 Key Laboratory of Neuroregeneration of Jiangsu Province and the Ministry of Education, Co-innovation Center of Neuroregeneration, Nantong University; Center for Basic Medical Research, Medical School of Nantong University; Department of Anesthesiology, Affiliated Hospital of Nantong University, Nantong, Jiangsu Province, China

**Keywords:** apoptosis, cerebral ischemia model, cognitive decline, hippocampal neurons, hippocampus, mitochondrial dysfunction, neuroinflammation, neuroprotection, neurovascular unit, poststroke cognitive impairment

## Abstract

Ischemic stroke has a higher survival rate and is more likely to result in cognitive impairment than hemorrhagic stroke. The primary pathological mechanism underlying cognitive impairment involves dysfunction of neural circuits and damage to specific brain regions. This review aims to investigate the role of the hippocampus in cognitive impairment following a stroke. A review of the literature suggests that the hippocampus is a metabolically active structure that is easily involved in various metabolic states, such as hypoxia and hypoglycaemia. The functional changes in hippocampal cells associated with poststroke cognitive impairment mainly manifest as neuronal apoptosis, impaired synaptic plasticity, and decreased neurogenesis. The primary pathological mechanism of poststroke cognitive impairment involves a complex cascade of reactions, including neuroinflammatory activation, bursts of oxidative stress, and neuronal apoptosis induced by mitochondrial dysfunction. Interventional drugs for cognitive impairment after cerebral ischemia include neuroprotective drugs, traditional Chinese medicines and their extracts, and stem cell therapies. Many of these drugs have unique advantages, including the inhibition of neuroinflammation, the prevention of apoptosis, and the promotion of neurogenesis. They hold great potential for the prevention and treatment of cognitive impairment following cerebral ischemia. However, most current studies are animal experiments, and relatively few clinical studies exist. In future research, emphasis should be placed on interventions for cognitive impairment following cerebral ischemia. These findings offer novel perspectives for the treatment of cognitive impairment after cerebral ischemia. Finally, the role of hippocampal cell dysfunction in other diseases associated with cognitive decline is briefly discussed. The aim of this review is to provide researchers with a comprehensive overview of the role of the hippocampus in cognitive impairment and its intervention strategies.

## Introduction

Cognition is a process in which the human brain takes in external information, processes it, transforms it into internal psychological operations, and acquires knowledge or applies that knowledge (Miller et al., 2024). It encompasses memory, language, visual-spatial skills, executive function, calculation, comprehension, and judgment (Wang et al., 2024). Cognitive impairment refers to the deterioration of one or more of the aforementioned cognitive functions, which impacts an individual’s daily and social capabilities (Lee and Jung, 2024; You et al., 2024). With the increasingly evident trend of population aging, the issue of cognitive impairment has attracted widespread attention (El Husseini et al., 2023).

The primary pathological mechanism of cognitive impairment is closely related to the dysfunction of neural circuits and cell damage in specific brain regions. Pathological processes such as neuroinflammation, abnormal protein aggregation (e.g., amyloid-β [Aβ] and tau protein aggregation), and neurotransmitter imbalance selectively target key circuit nodes (e.g., the hippocampus and prefrontal cortex), resulting in decreased information transmission efficiency and disrupted network connectivity, which ultimately manifests as a decline in specific cognitive functions (Mok et al., 2024). Recent studies have revealed that several brain regions, such as the medial temporal lobe, prefrontal cortex (Miller and Cohen, 2001; Friedman and Robbins, 2022), parietal cortex (Culham and Kanwisher, 2001; Cabeza et al., 2012; Freedman and Ibos, 2018), amygdala (Gallagher and Chiba, 1996; LeDoux, 2000; Meisner et al., 2022), basal nucleus of Meynert (Chen et al., 2023), and striatum (Chersi and Burgess, 2015), are confirmed to be involved in cognitive function. The medial temporal memory system is composed of the hippocampus and adjacent related cortices, including the entorhinal cortex, perirhinal cortex, and parahippocampal cortex, which play a crucial role in long-term memory, specifically declarative memory (Squire and Zola-Morgan, 1991; Clark, 2018). As a key hub of the limbic system, the hippocampus has become the focus of research on cognitive decline due to its central role in memory encoding, spatial navigation, and situational integration. Over the past two decades, advancements in optogenetics, single-cell sequencing, and *in vivo* calcium imaging have gradually revealed the specific contributions of heterogeneous cell populations in the hippocampus, such as CA1–CA3 pyramidal cells, dentate gyrus (DG) granule cells, and interneurons, to the maintenance of cognitive function. An early study primarily focused on the relationship between hippocampal atrophy and cognitive impairment-related diseases (Jia et al., 2025). However, recent findings indicate that the process of cognitive decline often precedes obvious structural changes, suggesting that microscopic cellular dysfunction may serve as a more sensitive warning indicator. For example, reduced dendritic spine density in pyramidal cells in the CA1 region disrupts the neural representation of spatial memory, while neurogenic defects in granule cells in the DG are directly associated with reduced pattern separation ability. Notably, glial cells indirectly exacerbate neuronal network imbalance by altering the local microenvironment, such as through glutamate reuptake disorders in astrocytes and abnormal synaptic pruning in microglia (Lee et al., 2021). These findings challenge the limitations of the traditional “neuron-centric theory” and emphasize the dynamic balance of cell interaction networks in the maintenance of cognition.

The causes of cognitive impairment are multifactorial, with cerebrovascular events (e.g., cerebral hemorrhage and infarction) and neurodegenerative conditions serving as core drivers. Inflammation, metabolic abnormalities, and psychological states act synergistically to accelerate the disease course. Clinical evaluation requires the integration of imaging, biomarkers, and neuropsychological testing. However, a central yet not fully answered scientific question remains: why do vascular events, neurodegenerative lesions, or metabolic abnormalities trigger the dysfunction of critical cells (e.g., neurons and neural stem/progenitor cells) in specific brain regions (e.g., the hippocampus) and ultimately lead to clinical symptoms such as memory loss and executive dysfunction? Furthermore, a previous study has shown that neurogenesis in the DG of the hippocampus, a rare process involving the continuous generation of new neurons in the adult brain, plays a key role in learning, memory (especially pattern separation), and emotion regulation (Qin et al., 2022). A decline in or impairment of nerve regeneration capacity is an important early pathological feature of various cognitive disorders (e.g., Alzheimer’s disease [AD] and vascular dementia). However, the changes that occur at different stages of the disease, the specific mechanisms of damage (e.g., microenvironment inhibition, stem cell depletion, and failure of newborn neuron integration), and the differences in nerve regeneration ability among different stages of the disease are not yet clear (Xing and Bai, 2020). Additionally, the influence of interactions with other pathological factors, such as neuronal loss, glial activation, and vascular lesions, still needs to be systematically elucidated.

**Figure 1:**
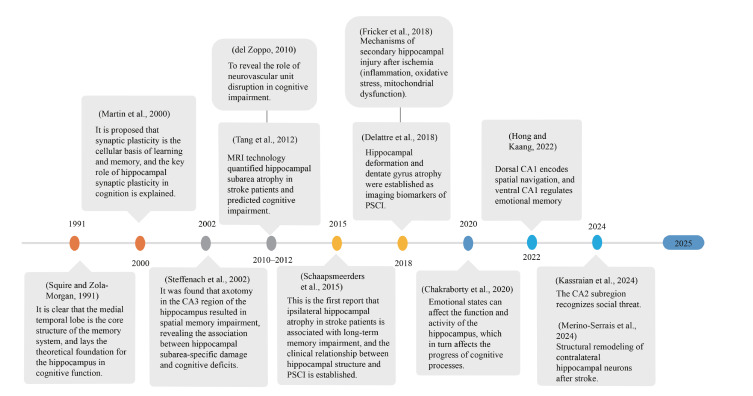
Timeline of research progress on the relationship between the hippocampus and post-stroke cognitive impairment (PSCI).

The annual incidence of cerebrovascular events (e.g., stroke) is high in China, with approximately 30%–70% of patients developing poststroke cognitive impairment (PSCI), which is a main type of vascular dementia (Tu and Wang, 2023). Compared with neurodegenerative diseases (e.g., AD), PSCI has a clear etiology (e.g., acute neuronal damage caused by ischemia or hemorrhage), preventable risk factors (e.g., hypertension and diabetes), and an effective window for intervention (a golden period within 6 months after stroke). Antihypertensive therapy, antiplatelet therapy, and cognitive rehabilitation can considerably improve patient prognosis, with some patients even achieving functional reversal (Wang et al., 2017). However, the mechanisms of neurodegeneration are complex (e.g., Aβ deposition and tau protein tangling), and there is a lack of effective blocking methods. Therefore, this review provides a brief introduction to the relationship between hippocampal cell dysfunction and cognitive decline, focusing specifically on PSCI. It elaborates on the role and mechanisms of action of hippocampal somatic cells, particularly regarding nerve regeneration disorders, neuronal damage, and glial cell activation, in the development of PSCI, as well as effective treatment measures. This review highlights the possible side effects and potential neurotoxic effects associated with treatment techniques for hippocampal dysfunction in PSCI, particularly focusing on invasive or intensive interventions. It provides crucial warnings and clinical references to enhance safety (**Figure 1**). Furthermore, it briefly investigates the role of hippocampal cell dysfunction in other diseases linked to cognitive decline. Ultimately, this review aims to offer researchers a comprehensive understanding of the hippocampus’s role in cognitive impairment and the currently available intervention strategies. Its primary goal is to promote the future development of precise diagnostic tools and effective, safe, individualized treatments that target hippocampal pathology.

## Search Strategy

A comprehensive search was conducted in both the PubMed and Web of Science databases. The search period spanned from January 2015 to December 2024, utilizing the following search terms to identify relevant papers: “Hippocampus,” “Hippocampal cells,” “Cognitive impairment,” “Cognitive decline,” and “Cognitive decline.” Initial screening was based on the title and abstract, excluding case reports, conference abstracts, studies that did not focus on the hippocampus, studies that did not assess cognitive function, those with incomplete data, those with low methodological quality, and studies that examined only other brain regions or unrelated diseases. Studies focusing on the role of hippocampal cells in PSCI were emphasized. Additionally, studies investigating the impact of hippocampal cells on other related degenerative diseases were included. The references from the included studies and relevant reviews were subsequently searched retrospectively to supplement the potential literature. The final selection encompassed literature reviews and studies involving animal simulation experiments.

## Structure, Function, and Neural Signaling of the Hippocampus

The hippocampus is an integral component of the brain’s limbic system, comprising the cornu ammonis (CA), DG, and subiculum. All these structures are located near the temporal lobe and are tightly interconnected with the adjacent entorhinal cortex. Anatomically, the hippocampus is described as a medial extension of the temporal horn within the lateral ventricle. It consists of four regions: CA1, CA2, CA3, and CA4. The CA1 region is connected to the subiculum, while the CA4 region is located within the hilum of the DG (Pronier et al., 2023; Lang et al., 2024). Histologically, the hippocampus and DG belong to the archicortex and each contains three layers: a molecular layer, a pyramidal cell layer (in the hippocampus), and a granule cell layer (in the DG), along with a polymorphic layer (Kominami et al., 2023). The principal neurons in the hippocampus are pyramidal cells, which are organized into distinct laminae, with their dendrites oriented towards the hippocampal sulcus. Pyramidal cells are crucial to hippocampal function, particularly in the CA1 and CA3 regions, and are especially important for memory function (Steffenach et al., 2002; Mertens et al., 2024). In addition to the pyramidal cell layer, the hippocampus contains the polymorphic cell layer, which consists of multiple cell types that also contribute critically to hippocampal function. The molecular layer, predominantly composed of nerve fibers and neurotransmitter receptors, contains few cellular elements involved in hippocampal physiology (de Oliveira et al., 2020; Yasuda et al., 2022). The DG is primarily populated by granule cells, which are small, densely packed neurons that serve as the principal neuronal type. Granule cells are essential for information processing and memory within the hippocampus (Sun et al., 2015).

The structure of the hippocampus is highly complex, with each region exhibiting distinct functions and characteristics. The hippocampus plays a crucial role in human episodic memory, learning, and spatial cognition.

In the hippocampus, the CA region is particularly important for memory consolidation and learning. The CA1 region is crucial for processing short-term memory and transforming information. This region interacts with adjacent hippocampal structures, such as the CA3 region, and cortical areas, such as the entorhinal cortex, to facilitate memory formation and consolidation. Notably, studies (Hong and Kaang, 2022; Cope et al., 2023) have revealed that specialized neurons in the hippocampal CA1 region are responsible for processing positive information related to other mice, suggesting that this subregion plays an important role in the formation of social relationship memories. The hippocampus also contributes to spatial processing, with the CA1 region involved in processing spatial information and navigation. It is responsible for integrating spatial data from the entorhinal cortex and other inputs, enabling the organism to navigate its environment (Geiller et al., 2023) and determine its position.

However, the involvement of the CA1 region of the hippocampus in learning is primarily reflected in its role as a key hub for information transmission. The hippocampal CA1 region is an essential subregion that receives input from other hippocampal areas, such as the DG and CA3, and subsequently integrates this information before transmitting it to the rest of the brain. This process of information transmission is fundamental to learning, as it encompasses the reception, processing, and storage of information (Jeong and Singer, 2022). The CA1 region integrates a wide variety of information by receiving projection fibers from different pathways. This integration process aids in forming a complete memory representation, making learning more effective and enduring. By interacting with other hippocampal structures and facilitating neuronal connections, the CA1 region can enhance functional connectivity between related neurons, thereby consolidating memory (Grienberger and Magee, 2022; Bai et al., 2023).

The neural mechanisms of learning and memory in the hippocampal CA2 region involve numerous interactions and regulatory processes. These mechanisms encompass episodic memory formation, social odor discrimination, associative learning, and the regulation of social aggression memory (Hassan et al., 2023). Hippocampal CA2 pyramidal neurons can differentiate allogenic social odors, with this ability enhanced following reward association learning. Not only do CA2 neurons recognize social odors, but they also associate them with emotional cues, such as rewards or punishments, through associative learning. This process aids animals in making adaptive behavioral decisions in social contexts (Hassan et al., 2023).

The dorsal CA2 subregion (dCA2) plays a crucial role in regulating social novelty recognition and aggression. dCA2 drives adaptive aggression by distinguishing between novel and familiar conspecifics. When exposed to an unfamiliar animal, dCA2 neurons are activated, encoding new information and initiating an aggressive response. Additionally, the dCA2 region can promote aggression toward new conspecific species by integrating social novelty information with behavioral state signals (Kassraian et al., 2024). This integration mechanism enables the animal to engage in adaptive aggression or avoidance behaviors in its environment.

The CA3 region of the hippocampus is crucial for memory formation and retrieval, the activity of place cells, the regulation of working memory, and associated neural mechanisms (Li and Roxin, 2023; Sheintuch et al., 2023; Sammons et al., 2024). Finally, the hippocampal CA4 region plays a vital role in the formation, storage, and retrieval of memories, including episodic memories. Moreover, its neural structure and connectivity enable it to communicate and interact with various other brain regions (Ono et al., 2021; Wong et al., 2021).

The DG region of the hippocampus plays a key role in memory formation and storage. It is a component of the hippocampal trisynaptic circuit involved in forming new episodic memories. Specifically, the hippocampal DG may contribute to memory processes by transforming similar input information into different patterns of neural activity during the formation of new memories, thereby avoiding memory confusion (Hainmueller and Bartos, 2020). Input information from different brain regions converges in the DG of the hippocampus, which can also promote the binding of this information to create a complete memory representation. The ongoing generation and turnover of immature neurons in the DG may help maintain selective activity in response to experiences during development, thus aiding in indexing a neocortical representation of those experiences (Borzello et al., 2023). Neural stem cells (NSCs) in the DG may be activated and proliferate through the Wnt/β-catenin pathway after ischemia, promoting the integration of new neurons into the hippocampal circuit and enhancing spatial memory (U et al., 2025). The hippocampal DG is also associated with spatial orientation. Although this aspect has been relatively understudied, it is closely related to spatial memory and navigation abilities as part of the overall structure of the hippocampus. Therefore, the hippocampal DG region may also be involved in these processes, although the specific mechanisms of action are not yet fully understood.

At present, learning and memory functions, which are related to the large number of neurons in the brain, are the main concerns of researchers. The connections between these neurons, known as synapses, play a crucial role in the process of memory formation. Neurons rely on electrochemical signals to communicate with one another, thus constructing an intricate neural network. Through the synaptic connections between neurons, the hippocampus encodes the received information and stores it in the brain (Alam et al., 2018). By means of neuronal activation and changes in connection strength, the hippocampus is able to encode and store new memory information. Neurons possess structurally variable dendritic spines on their dendrites, which create abundant connections between neurons, allowing the encoding capacity for information to grow exponentially. This process is essential for individual learning and memory abilities (Borczyk et al., 2021).

As a pivotal medial temporal lobe structure, the hippocampus engages in extensive functional connectivity with distributed brain regions to mediate higher-order cognition. Anatomical studies have revealed robust hippocampal-entorhinal interactions, with the entorhinal cortex serving as the principal afferent hub that integrates neocortical sensory and cognitive inputs (Roy et al., 2017; Lopez-Rojas et al., 2022). This connectivity architecture critically supports information synthesis for both episodic memory formation and social memory processing. In the hippocampal–prefrontal circuits, the prefrontal cortex plays a key role in executive function, decision-making, and attention control, while the hippocampus is essential for learning and memory. The connection between the two may involve the formation and maintenance of working memory, as well as the consolidation of long-term memory (Tang et al., 2023). It has also been suggested that the hippocampal-prefrontal circuits play important roles in episodic memory and that the prefrontal cortex and hippocampus are coupled through oscillatory synchronization, reflecting bidirectional information flow (Tanninen et al., 2017). The medial prefrontal cortex and hippocampus work together to supply memory via multiple pathways. These pathways support synchronization as well as the bidirectional exchange of specific information (Eichenbaum, 2017). Furthermore, the hippocampus is closely related to the thalamus (Leszczyński and Staudigl, 2016), cingulate cortex (Remondes and Wilson, 2013), amygdala (Yang and Wang, 2017), and striatum (van de Ven et al., 2020). The hippocampus is not only associated with memory but is also involved in spatial cognition and navigation processes. Spatial maps and navigation paths are formed by integrating spatial information from multiple senses, such as vision and hearing, through interactions with other brain regions (Ekstrom and Ranganath, 2018; Rolls and Wirth, 2018). These regions interact and collaborate to promote learning, memory, spatial navigation, and other cognitive functions. Additionally, the hippocampus is closely related to emotion regulation, and emotional states can affect its function and activity, in turn influencing the progression of cognitive processes (Chakraborty et al., 2020).

**Figure 2:**
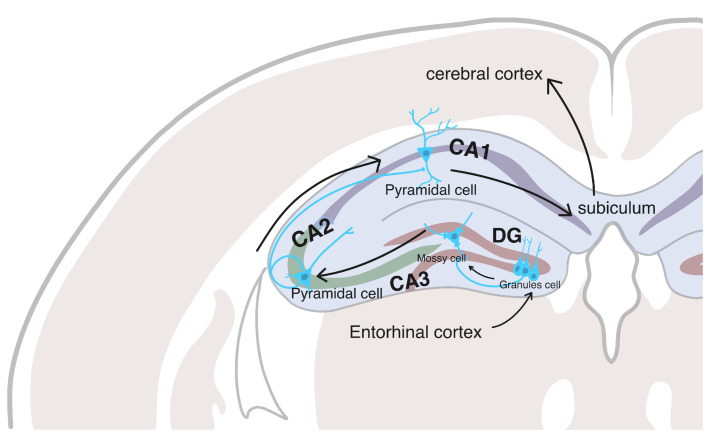
Hippocampal information transmission pathways. The entorhinal cortex-hippocampus pathway (the transmural pathway) inputs sensory/cognitive information into the dentate gyrus (DG) and CA3 area. The three-synaptic circuit (DG→CA3→CA1) forms a closed loop, which is related to long-term memory formation and long-term potentiation. The hippocampus-thalamus-cortex pathway outputs the processed information to the cortex for memory consolidation and retrieval. Together, these three pathways constitute the hippocampal information network, the integrity of which is critical for memory.

The hippocampus mediates information processing through electrochemical synaptic transmission across three principal interconnected pathways (**Figure 2**), forming a sophisticated neural communication network. First, the entorhinal cortex-hippocampus pathway: The entorhinal cortex serves as the convergence point for the ascending pathways of the main sensory areas of the neocortex and is the primary information source for the hippocampus, responsible for transmitting sensory and cognitive information from the cerebral cortex to the hippocampus. Information travels from the entorhinal cortex to the DG and the CA3 region of the hippocampus via the perforant pathway (Deller et al., 1996). Second, the trisynaptic circuit: This closed-loop circuit forms within the hippocampus, encompassing the DG, CA3 region, and CA1 region. Among them, information first reaches the granule cells in the DG. After the integration of the first synapse, the axons of the granule cells transmit signals to the CA3 area of the hippocampus through the mossy fibers, forming the second synapse (Henze et al., 2000). Subsequently, the axons of the CA3 area convey neural information to the CA1 area through the Schaffer collaterals, resulting in the third synapse (Smith et al., 2003). Pyramidal cells in the CA1 area project to multiple target areas through the subiculum and hippocampal fimbriae, allowing information to be transmitted to other brain areas, such as the thalamus and brainstem, or back to the entorhinal cortex through reverse connections, forming feedback loops. The trisynaptic circuit serves as a connection between the entorhinal area and the hippocampal DG, which may be related to the formation of long-term memory. This is linked to the phenomenon of long-term potentiation (LTP), which supports the mechanism of long-term memory (Safari et al., 2021; Lee et al., 2023). Moreover, studies have found that this pathway induces an enhanced LTP phenomenon, indicating synaptic plasticity (Bowden et al., 2012; Ahnaou et al., 2020). Third, the hippocampal-thalamo-cortical pathway: This pathway involves connections between the hippocampus and the thalamus, particularly the anterior thalamic nucleus, as well as the cerebral cortex. Through this pathway, the hippocampus transmits processed information back to the cerebral cortex to support memory consolidation and retrieval. It has been suggested that hippocampal cortical memory flow is transmitted from the entorhinal cortex to the hippocampus (DG, CA1, and CA3), and then follows the subiculum to the cortex (parahippocampus, prefrontal cortex, and retrosplenial cortex), accompanied by parallel CA1 projections to the parahippocampus and prefrontal cortex (Aggleton and O’Mara, 2022). The diencephalic memory pathway originates in the anterior thalamic nuclei, projecting efferents to the hippocampal subiculum, parahippocampal regions, and retrosplenial cortex. This pathway exhibits bidirectional connectivity, integrating afferent signals from hippocampal outputs, retrosplenial networks, anterior cingulate circuits, and prefrontal cortical areas.

These two parallel and partially independent streams of memory project to the para-hippocampus, prefrontal cortex, and retrosplenial cortex, where they communicate with one another, and synaptic plasticity promotes memory consolidation. Damage to any of these areas can result in memory impairment. This review focuses on PSCI and discusses on the role of the hippocampal soma in its development, mechanisms, and effective treatment measures.

## Stroke and Cognitive Decline

### Classification and pathological mechanism of stroke

Stroke is a disorder that results in damage to cerebral blood vessels due to various factors, leading to focal or global injury to brain tissue. It is characterized by high rates of incidence, disability, recurrence, and mortality. There are two main types of stroke: hemorrhagic and ischemic. Ischemic stroke (IS) accounts for 75% to 90% of all strokes (Fan et al., 2023).

IS is a general term for the necrosis of brain tissue and the consequent insufficient blood supply caused by stenosis or occlusion of the brain’s feeding arteries, namely the carotid and vertebral arteries. IS is a nervous system disease resulting from cerebrovascular pathology. From a pathological perspective, disorders of cerebral perfusion lead to hypoxic-ischemic injury, metabolic disturbances, and ultimately, necrosis of the brain parenchyma. Following cerebral vascular occlusion, neurons undergo substantial structural remodeling and activate regulated cell death pathways, including apoptosis and necroptosis cascades (Li et al., 2021a). Microglia rapidly infiltrate the lesion area and exert dual regulatory functions during neurotoxicity and neuroprotection through proliferation and phenotypic polarization. Astrocytes maintain metabolic homeostasis by precisely regulating extracellular ionic gradients and clearing excitatory neurotransmitters (Ranjbar Taklimie et al., 2019).

Postthrombolytic reperfusion in IS induces an abrupt restoration of oxygen and glucose metabolism, triggering excessive generation of reactive oxygen species (ROS). In the acute phase, ROS overproduction in ischemic tissue promotes oxidative stress and a systemic inflammatory response. Concurrently, pro-inflammatory mediators (i.e., cytokines and chemokines) and oxidative radicals are released into extracellular compartments. Cerebral endothelial activation increases the expression of adhesion molecules (e.g., vascular cell adhesion molecule 1 and intercellular adhesion molecule 1), facilitating leukocyte-endothelial interactions and blood–brain barrier (BBB) transmigration (Jin et al., 2013; Zhang et al., 2023b). In the subacute phase, the release of pro-inflammatory factors (including cytokines, chemokines, ROS, and matrix metalloproteinases) by infiltrating leukocytes exacerbates inflammation. This cascade activates local glial cells, enhances peripheral leukocyte recruitment, and culminates in BBB breakdown, brain edema, neuronal death, and hemorrhagic complications. However, some researchers suggest that the inflammatory response after ischemia may promote tissue repair and functional recovery in the chronic phase (Kamel and Iadecola, 2012; Zera and Buckwalter, 2020).

Intracerebral hemorrhage (ICH) is a common acute severe disease in neurology, associated with a high disability and recurrence rate. Blood flow into brain tissue following blood vessel rupture can cause severe damage. Early injury in ICH results from mechanical compression due to hematoma expansion: within the first 4 hours, physical destruction and stretching of surrounding neurons and glial cells trigger excessive release of neurotransmitters, calcium influx, and mitochondrial dysfunction, leading to cytotoxic edema and cell death (Magid-Bernstein et al., 2022; Zhang et al., 2022b). Over the following days, the hematoma becomes disorganized, releasing toxic metabolites such as thrombin and free ferrous ion (Fe^2+^) (Wang et al., 2018b). These metabolites activate oxygen free radicals, matrix metalloproteinases, complement proteins, and inflammatory markers. The downstream molecules then increase BBB permeability, attract inflammatory cells, and trigger apoptotic pathways, ultimately exacerbating brain edema and neuronal death (Ren et al., 2018).

### Relationship between stroke and cognitive decline

The diagnosis and treatment of stroke complications, especially PSCI, remain great challenges. PSCI is a clinical syndrome characterized by cognitive impairment that emerges following a stroke event and can last for up to 6 months. This includes cognitive impairment immediately after the stroke, as well as new or worsening cognitive impairment in the months that follow. The number of patients with stroke in China is the highest in the world, and the incidence of PSCI among patients with stroke has exceeded 50% (Chau et al., 2023). In other countries, more than 30% of patients with stroke experience PSCI, which places a heavy economic and mental burden on society and families (Wang et al., 2017; Tu and Wang, 2023). The occurrence of PSCI notably influences the rehabilitation, participation in activities, and recovery of other physical functions of patients. The potential effects and clinical challenges posed by PSCI have become hotspots in current related research. The occurrence and development of PSCI are related to many factors, such as the duration of the stroke, age, location and size of the infarction, degree of neuronal lesions, preexisting cognitive deficits, and other brain dysfunctions (Tang et al., 2018). The pathological mechanisms of PSCI are associated with acute focal brain injury and the triggering of strong secondary injury cascades (i.e., inflammation, excitotoxicity, BBB disruption, and apoptosis), white matter lesions, and changes in neuroplasticity (Sun et al., 2014). The anatomical basis of PSCI includes identifiable macroscopic lesions of brain injury (e.g., infarction/hemorrhage).

To date, although there have been certain advancements in research on PSCI, its high heterogeneity due to variations in stroke type, lesion location, and patient background as well as the extremely complex pathological mechanisms involving interwoven factors such as direct damage, neurodegeneration, vascular lesions, and inflammation, means that there is still no precise and efficient early warning mechanism or intervention measures for PSCI. This often results in patients with PSCI missing the optimal treatment and rehabilitation time points upon seeking medical attention (Sun et al., 2014; Levine et al., 2015). Therefore, in-depth research into the pathological mechanisms of PSCI remains a pressing issue in stroke research both domestically and internationally.

The hippocampus is the most important brain region related to cognitive functions, including spatial learning and memory (Cohen and Stackman, 2015). Among its components, the vulnerability of pyramidal neurons in the CA1 area of the hippocampus plays a remarkable role in the onset of cognitive disorders. IS is more likely to cause memory-related cognitive impairments due to the greater vulnerability of the hippocampal region to blood flow, while hemorrhagic stroke has a greater impact on executive functions (Potter et al., 2021). As a result, the hippocampus has become an important model for studying the cellular and molecular basis of cognitive processes such as learning and memory.

In recent decades, scholars in the field of neuroscience have not only conducted in-depth studies on the cellular and molecular basis of cognitive processes in the hippocampus but have also utilized magnetic resonance technology to examine changes in the hippocampus related to cognitive impairments from both functional and structural perspectives (Schaapsmeerders et al., 2015; Zhao et al., 2021; Khlif et al., 2022). Research has shown that the hippocampus is composed of multiple structural regions, and neurons in different regions play distinct roles in functional damage after stroke, repair, and the occurrence of cognitive impairments. Understanding the role and mechanisms of action of cells in different regions of the hippocampus in cognitive impairment after stroke is crucial for formulating effective prevention and treatment strategies.

## Commonly Used Animal Models of Cognitive Impairment after Stroke

### Vascular supply of the hippocampus

The blood supply to the brain primarily comes from two systems: the internal carotid artery system and the vertebrobasilar arterial system. The internal carotid artery system supplies blood to the anterior three-fifths of the cerebral hemisphere, also known as the anterior circulation. The key arteries in this system include the left and right internal carotid arteries (ICA), which branch into the middle cerebral artery (MCA) and the anterior cerebral artery. These arteries are crucial for cognitive functions such as decision-making, language, and motor control. Vascular issues in the anterior circulation are common in strokes and transient ischemic attacks, leading to symptoms such as unilateral limb weakness and speech disorders (Wolman et al., 2022). The vertebrobasilar system is responsible for supplying blood to the posterior two-fifths of the cerebral hemisphere, the brainstem, and the cerebellum, collectively known as the posterior circulation. This system is formed by the convergence of the left and right vertebral arteries into the basilar artery, which further divides into branches, including the posterior cerebral artery (PCA), the superior cerebellar artery, the anterior inferior cerebellar artery, and the posterior inferior cerebellar artery (Reyes-Soto et al., 2024). These arteries provide blood for functions such as visual processing, balance, coordination, and vital centers that regulate breathing and heartbeat. Vascular problems in the posterior circulation can result in symptoms such as vertigo, visual impairment, dysphagia, and even loss of consciousness. The two arterial systems are interconnected through the circle of Willis, forming a complex vascular network (Berghout et al., 2025).

**Figure 3:**
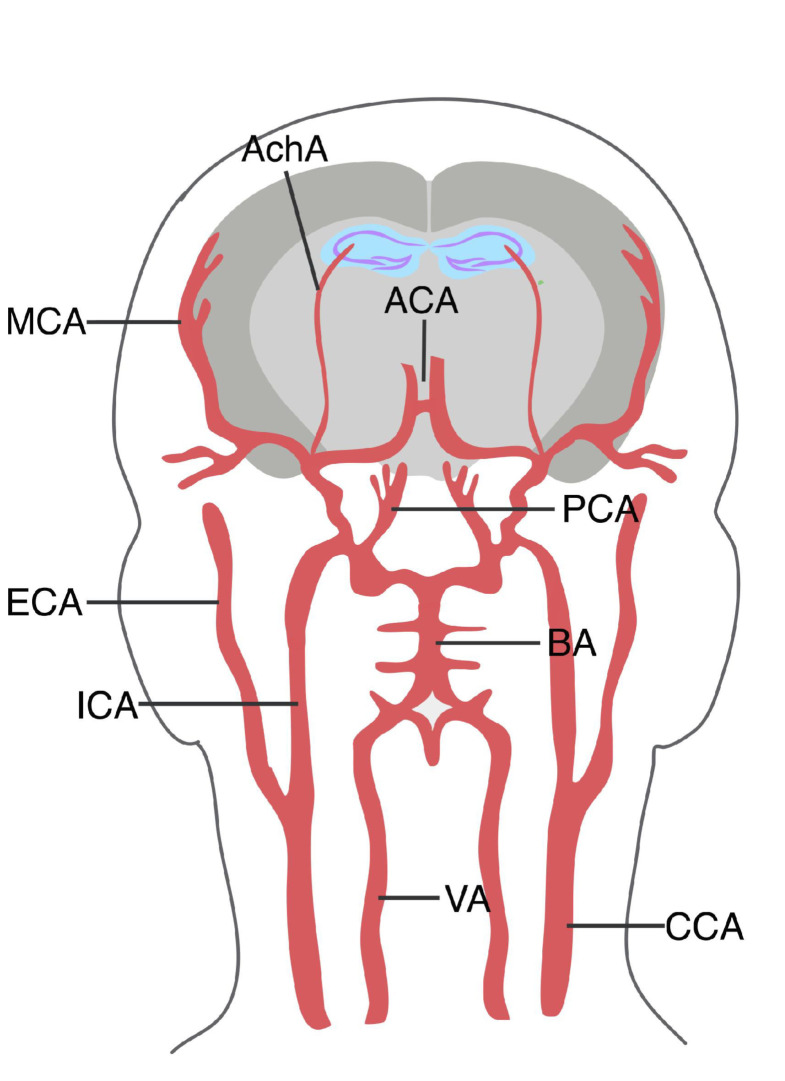
Origin and branch distribution of blood supply in the hippocampus. MCA is the largest branch of ICA and serves as the primary artery supplying the anterior cerebral circulationn. The blood supply to the hippocampus primarily comes from the PCA, which originates from the BA. Its branches, including the anterior, middle, and posterior hippocampal arteries, supply the head, body, and tail of the hippocampus, respectively. In some individuals, the AChA, which originates from the ICA, may also contribute to the blood supply, resulting in a mixed vascular pattern. ACA: Anterior cerebral artery; AchA: anterior choroidal artery; BA: basilar artery; CCA; common carotid artery; ECA: external carotid artery; ICA: internal carotid artery; MCA: middle cerebral artery; PCA: posterior cerebral artery; VA: vertebral artery.

The hippocampus requires robust vascular perfusion to maintain cognitive function (**Figure 3**). Its blood supply is derived from two primary sources: a combined input from the PCA and the anterior choroidal artery (AChA), or exclusively from the PCA. This dual-source pattern enhances hemodynamic stability, potentially protecting against cognitive decline during ischemic events (Perosa et al., 2020). The PCA originates from the basilar artery and gives rise to the anterior hippocampal artery, middle hippocampal artery, and posterior hippocampal artery. The anterior hippocampal artery supplies the head of the hippocampus, while the middle and posterior hippocampal arteries supply the body and tail, respectively. Most of the AChA arises from the posterior lateral wall of the internal carotid artery, with a smaller number originating from the MCA or the posterior communicating artery (Erdem et al., 1993). Although it is generally accepted that the PCA supplies the majority of blood to the hippocampus, the regional contribution of blood flow from the PCA can vary due to the heterogeneity of the circle of Willis. This unique blood supply pattern makes the hippocampus particularly sensitive to ischemia. Previous studies have shown a positive correlation between whole-brain or hippocampal blood flow and cognitive function (Rosen et al., 1984; Ois et al., 2009; Iliff et al., 2013). Ischemia can lead to a series of pathological changes in the hippocampus, potentially resulting in conditions such as hippocampal sclerosis.

In humans, the PCA is predominantly supplied by the vertebrobasilar arterial system. As a result, the posterior circulation serves as the primary source for vascular perfusion of the hippocampus. Conversely, in rodents (for example, rats), the PCA is mainly supplied by the carotid artery through the posterior communicating artery. Thus, a larger proportion of hippocampal blood flow originates from the anterior circulation (Goetzen and Sztamska, 1992). In smaller mammals such as rats, proximal occlusion of the MCA may lead to ischemic damage in the anterior hippocampus (El Amki et al., 2015). Building on this vascular framework of hippocampal blood supply, we will elaborate on common methods for creating an animal model of cognitive impairment following cerebral ischemia (**Additional Table 1**).

**Additional Table 1:** Characteristics of commonly used animal models of cognitive impairment after ischemic stroke

Ischemic model	Time of onset of cognitive impairment	Affected cognitive domain	Advantage	Disadvantage	Reference
Middle cerebral artery occlusion	14 d-6 mon	Spatial learning/memory, short-term memory, anxiety-like behavior, associative memory, social ability, and recognition memory	Avoid craniotomy; allow precise temporal control; suitable for experimental studies	Technical challenges may lead to variable infarct volumes, vascular rupture, or subarachnoid hemorrhage	Delattre et al., 2018; Kim et al., 2020
BCAO	BCAO: 1 d-3 mon BCAS: 1-6 mon	Spatial working memory, spatial learning/memory, short-term memory, and object recognition memory	High reproducibility; quantifiable outcomes; tunable occlusion duration; applicable to reperfusion studies	BCAO rarely induces long-term neurological deficits; BCAS causes prolonged cognitive impairment	Gooch and Wilcock, 2016; Ojo et al., 2019; Ishikawa et al., 2023
Four-vessel occlusion	7-40 d	Spatial recognition memory, spatial learning/memory, anxiety-like behavior, and object recognition memory	Technically simple; high predictability of neuronal damage; low seizure incidence	Risk of visual deficits; elevated mortality; frequent postoperative complications	Barros et al., 2009; Wen et al., 2014; Zhang et al., 2019; Sun et al., 2021
Photochemical ischemia	2-28 d	Spatial learning/memory, associative memory, and social memory	Minimally invasive; precise lesion targeting; high experimental reproducibility	Rapid ischemic penumbra collapse; poor clinical translatability	Ong et al., 2018; Sun et al., 2020a; Hossein Geranmayeh et al., 2023

BCAO: Bilateral common carotid artery occlusion; BCAS: bilateral common carotid artery stenosis

### Commonly used animal models of cognitive impairment after ischemic stroke

***Middle cerebral artery occlusion***

The animal model of middle cerebral artery occlusion (MCAO) is a globally acknowledged method for researching cerebral ischemic injury. In human IS, the MCA and its branches are the cerebral vessels most often affected, accounting for approximately 70% of infarcts. Therefore, occluding this artery closely mimics human ischemic stroke (Li and Zhang, 2021). The typical approach involves exposing the unilateral common carotid artery (CCA), external carotid artery (ECA), and ICA. Once the ECA is ligated, a silicone nylon wire with a rounded end is inserted into the ICA via the ECA to obstruct the MCA (Gong et al., 2023). Alternatively, the ECA can be ligated, and a thread plug can be inserted into the ICA via the CCA (Wang et al., 2023). The difference between ICA and ECA emboli lies in the late reperfusion caused by embolus removal. CCA emboli require ligation of the proximal CCA, allowing reperfusion through the circle of Willis via the contralateral CCA. In contrast, ICA insertion does not require ligation of the CCA, and reperfusion occurs through the bilateral common carotid arteries (Li et al., 2023d).

MCAO can lead to damage in the cortex, striatum (Li and Zhang, 2021), and hippocampus (He et al., 2018). However, the degree of infarction is determined by factors such as the location and duration of occlusion, as well as the volume of collateral blood in the MCA. The duration of ischemia can be categorized as transient or permanent. Transient MCAO (tMCAO) can cause damage to neurons in the ischemic core area and non-ischemic areas away from the ischemic core, such as the lateral substantia nigra and hippocampus. For example, tMCAO causes severe and acute harm to neurons and oligodendrocytes in the ipsilateral hippocampal CA1 region (Uchida et al., 2010). Permanent MCAO (pMCAO) leads to extensive ischemic damage in the MCA territory and is characterized by remarkable brain edema and notable lesion expansion caused by delayed ischemic cell death (Shanbhag et al., 2016).

A previous study revealed that a reduction in hippocampal volume in patients subjected to MCAO, assessed through volumetric magnetic resonance imaging and proton magnetic resonance spectroscopy, caused secondary hippocampal damage, which in turn led to cognitive impairment (Tang et al., 2012). Considerable hippocampal deformation and entorhinal cortex atrophy were observed 6 months after ischemia in both post-MCAO rats and stroke patients with cognitive impairment (Delattre et al., 2018). When MCAO occurs in rodents, a decrease in cerebral blood flow (CBF) affects not only the MCA region but also the hippocampus, thalamus, and hypothalamus. Neurotoxic molecules generated in the region predominantly supplied by the MCA can disseminate to the hippocampus, resulting in ischemic injury. Therefore, hippocampal damage may be due to excitation of the hippocampal neuron network following MCAO (El Amki et al., 2015).

Within 28 days after MCAO-induced ischemic stroke (lasting 45 minutes), the escape latency of mice in the Morris water maze test was remarkably prolonged, indicating that their spatial learning and memory abilities were obviously impaired (Kim et al., 2020). Similarly, hippocampal PSD-95 was suppressed 28 days after MCAO-induced ischemic stroke, the number of platform crossings was reduced, and spatial learning and memory in rats gradually declined (Wang et al., 2023).

This model has several advantages including no craniotomy and accurate control of reperfusion and infarction times, making it convenient for experimental research. However, unskilled technical application may lead to inconsistent infarct sizes and could even trigger vascular rupture, resulting in subarachnoid hemorrhage.

#### Bilateral common carotid artery occlusion

Another common model used to simulate cognitive impairment in cerebral ischemia is bilateral common carotid artery occlusion (BCAO), which results in symptoms related to chronic cerebral hypoperfusion. The changes in cerebral blood flow (CBF) occur in three phases: the acute phase (lasting up to 2–3 days post-occlusion), the chronic hypoperfusion phase (lasting from 8 weeks to 3 months), and the recovery phase (Li and Zhang, 2021). At 3 days after BCAO in rats, the BBB is disrupted, as indicated by an increase in the apparent diffusion coefficient, suggesting vasogenic edema (Yang et al., 2016). The common method for inducing BCAO involves isolating the bilateral common carotid arteries in rats and permanently ligating them with surgical silk sutures (Zhao et al., 2014). Alternatively, the technique can be modified: first, the right common carotid artery can be occluded, and then, after 1 week, the left artery can be occluded (Cechetti et al., 2012). BCAO induces neuronal death in the hippocampus, caudate nucleus, and cerebral cortex (Kitagawa et al., 1998).

In mice, hypoplasia of the circle of Willis exacerbates ischemic severity in common carotid artery occlusion models. For animals with hypoplasia of the circle of Willis, such as Mongolian gerbils (Shin et al., 2022) and C57BL/6 mice (Han et al., 2020), transient carotid occlusion or bilateral common carotid artery stenosis (BCAS) are often chosen. A transient ischemic attack involves exposing both common carotid arteries and occluding them for a certain period before removing the occlusion to restore cerebral blood flow. A study examining the effects of 20 minutes of transient cerebral ischemia in mice (Li et al., 2023a) revealed disruption of neurovascular ultrastructures, evidenced by the destruction of neuronal morphology, thickening and edema of the capillary walls, and swelling of the terminal foot processes of hippocampal astrocytes. Through the novel object recognition test, it was found that the discrimination rate in the model group was lower, indicating decreased recognition memory ability. The Morris water maze results showed that spatial learning and memory were notably diminished, suggesting that the mice likely suffered from cognitive impairment (Gooch and Wilcock, 2016; Ojo et al., 2019; Ishikawa et al., 2023). In the case of BCAS, the model is established by positioning microcoils around both carotid arteries for partial occlusion, with the severity of occlusion influenced by the diameter of the coil. Coils with a diameter smaller than 0.2 mm lead to an acute reduction in CBF, while coils with a diameter of 0.22 mm have no effect on CBF (Tuo et al., 2021). When a microcoil (0.18 mm) was wrapped around the common carotid artery to establish a cognitive impairment model, it was discovered that BCAS-induced white matter damage resulted in cognitive decline. The Y-maze test demonstrated impairment in short-term working memory, which could be mitigated by knocking down CXCL5 in astrocytes (Cao et al., 2023b).

Following BCAO, increased blood flow fluctuations in the vertebral and basilar arteries allow the unaffected vasculature of the brain to largely compensate for the occlusion (Rots et al., 2019). This compensatory mechanism provides an incomplete blood supply to the brain through the basins of the right and left posterior cerebral arteries and the circle of Willis. Despite these compensatory mechanisms, BCAO can lead to cerebral circulation defects, resulting in diffuse structural and functional injury (Artyukhina and Sarkisova, 2007). BCAO in rats is associated with severe white matter pathology and hippocampal neuron damage. Mice also exhibit white matter damage following BCAS, indicating that hippocampal atrophy caused by BCAS is attributable to pathology in the white matter closely connected to the hippocampus. This damage can indirectly induce hippocampal atrophy through long-range effects. Additionally, CBF in the hippocampus is remarkably reduced after BCAS (Washida et al., 2019).

This model has several advantages: a high success rate for modeling, quantifiable outcome measures, and a tunable occlusion duration according to experimental needs, allowing for accurate control of the degree of cerebral ischemia. These features render it suitable for vascular reperfusion research. However, the success of this model depends on the appropriate strain and a precise understanding of the timing of ischemia (Yang et al., 2016). Specifically, cognitive impairment caused by BCAO tends to be of relatively short duration, while long-term neurological dysfunction is less common. Notably, BCAS elicits prolonged cognitive deficits with longer periods of occlusion

#### Four-vessel occlusion

In addition to BCAO, four-vessel occlusion (4VO) can also be performed for global cerebral ischemia. Because permanent occlusion is associated with high mortality and common complications, transient ischemic occlusion is usually selected. Cerebral blood flow is reduced to 95% after 4VO compared with 50% after BCAO (Jaspers et al., 1990). A modified version of the common method is permanent ligation of the vertebral artery outside the atlantoaxial joint between the first and second transverse processes using nylon surgical sutures. Twenty-four hours later, animals are subjected to a period of global cerebral ischemia by temporary occlusion of both CCAs with vascular clips (Sun et al., 2021). Electrocoagulation of the vertebral artery is avoided due to risks of hemorrhagic complications, spinal cord trauma, and incomplete occlusion.

4VO induces neurodegeneration in the hippocampal CA1 region, striatum, and neocortex, with lesion patterns resembling those of two-vessel occlusion (McBean and Kelly, 1998). Despite severe hippocampal pathology, the modified 4VO protocol spares visual pathways, as evidenced by intact optic tract morphology and normal light‒dark box performance (Sun et al., 2021), suggesting that cognitive deficits arise directly from hippocampal dysfunction. This model can lead to severe death of hippocampal neurons in the CA1 region, resulting in learning and memory deficits, as shown by the water maze (Wen et al., 2014). At 24 hours after the electrocoagulation of the two vertebral arteries, rats underwent transient CCA occlusion for 10 minutes, resulting in the loss of hippocampal synapses and dendritic spines. The novel object recognition test and Barnes maze test revealed that spatial memory and recognition ability were reduced and that cognitive function was impaired (Barros et al., 2009; Zhang et al., 2019).

When ischemia occurs, the pyramidal cells in the hippocampus are damaged, and the lesions in CA1 are more severe than those in CA3. In contrast, the onset of cellular necrosis in CA3 is delayed compared with that in CA1 (Ordy et al., 1993). The biochemical mechanisms underlying the slow pathology of hippocampal neurons are not well understood. Mechanisms triggered by ischemia may have sustained and deleterious, but not acutely lethal, effects on CA1 neurons (Kirino et al., 1984).

The advantages of this model include its technical simplicity, high reproducibility of ischemic neuronal lesions, and low incidence of seizures. However, the disadvantages are that the traction on the surrounding tissues is severe, the restoration of blood flow is easily affected, and the vertebral artery is permanently blocked. In addition, the model may lead to visual impairment and thus affect the accuracy of the cognitive impairment results (Tuo et al., 2021). Owing to high mortality rates and common complications, experimental animals require more meticulous postoperative care.

#### Photothrombotic stroke

Photothrombotic stroke (PTS) serves as a model of permanent cerebral ischemia. Compared with other models, the PTS model has several advantages. One of its key strengths is the predictability of the ischemic lesion. Additionally, the ischemic location is clearly defined, as it is determined by directing the laser beam at a prespecified brain area. The extent of the lesion is regulated by two factors: light intensity and duration of exposure (Labat-Gest and Tomasi, 2013). Due to accurate stereotactic positioning during irradiation and continuous monitoring of light intensity, the resulting lesion exhibits excellent reproducibility. This method is simple to perform, has low invasiveness, requires less surgical intervention, and has a low mortality rate in animals (Uzdensky, 2018). However, this model has the disadvantages of lacking ischemic penumbra areas and collateral blood flow. The common method for this model involves applying a photosensitive dye (e.g., Rose Bengal) to a specific area and then irradiating that area with light of a specific wavelength through the skull to activate the dye. This leads to the formation of singlet oxygen and superoxide, which, in turn, causes endothelial damage, platelet activation, and aggregation (Sommer, 2017). As a result, there is a rapid development of ischemic cell death in the illuminated brain area of the arterial terminal region, ultimately leading to cortical ischemic lesions.

Focal microinfarcts induced by PTS are linked to clinically “silent” strokes, where patients are unaware that they have had a stroke. However, over time, numerous clinically silent cerebral microinfarcts can result in nonfatal subclinical impairments of cognitive functions, including memory loss, difficulties in decision-making, and behavioral alterations (Uzdensky, 2018). One study established a photothrombotic model in C57BL/6 mice and assessed cognitive function and hippocampal neurogenesis using the Morris Water Maze test, Golgi staining, and immunohistochemical staining *in vitro* and *in vivo* (Sun et al., 2020a). Hippocampal neurons were found to have abnormal morphology characterized by short apical dendrites and a low density of spines along the dendrites (Sun et al., 2020a). Photothrombotic ischemia was induced in the left medial prefrontal cortex of adult male BALB/c mice, resulting in remrakably decreased social memory index and spatial learning and memory abilities, as demonstrated by the social interaction test and Barnes maze (Hossein Geranmayeh et al., 2023). To investigate the effects of recombinant human growth hormone on cognitive function, Ong et al. (2018) used a photochemical method to induce ischemia, performed a paired associative learning task on a touchscreen platform to evaluate cognitive function in mice, and found that the accuracy rate in the final paired associative learning stage was low.

PTS can target longitudinal hippocampal veins in adult mice and cause hippocampal ischemic injury (Blanco-Suárez, 2023). PTS directly induces ischemia in the hippocampus, and the necrotic area of the hippocampus is clearly demarcated. In ischemic regions, liquefactive necrosis leads to cavitation (Hosseini et al., 2018).

Due to its repeatability, ease of handling, minimally invasive nature, and flexible controllability of infarct size and location, this model has been widely used in rats and mice. However, this model leads to permanent occlusion of cortical terminal arteries, and reperfusion cannot be achieved, which is not conducive to the establishment of collateral circulation or the study of ischemia–reperfusion injury (Labat-Gest and Tomasi, 2013). Although an ischemic penumbra exists, it is narrow in extent and rapidly disappears, which is inconsistent with the clinical presentation of IS.

### Commonly used animal models of cognitive impairment after hemorrhagic stroke

#### Collagenase-induced intracerebral hemorrhage

The collagenase-induced intracerebral hemorrhage (ICH) model involves the stereotactic injection of collagenase to disrupt the vascular basement membrane, thereby inducing localized hemorrhage and mimicking the hematoma formation and inflammatory responses observed in human ICH. Due to its operational simplicity and high reproducibility, this model is extensively used to investigate the effects of acute-phase hematoma expansion, edema, and neuroinflammation on cognitive function. Previous studies have demonstrated that collagenase-induced ICH can lead to hippocampus-dependent spatial memory deficits (e.g., reduced performance in the Morris water maze test) and executive dysfunction (Shi et al., 2019; Cao et al., 2023a). Recently, Cao et al. (2023a) reported that collagenase-induced ICH may exacerbate cognitive impairment, suggesting that CAV1 plays a role in aggravating neurological dysfunction in a rat model of ICH.

#### Autologous blood injection model

The autologous blood injection model involves directly injecting arterial blood from an animal into the basal ganglia or cerebral cortex of its brain to simulate the space-occupying effects and blood toxicity associated with clinical cerebral hemorrhage (Fang et al., 2023). This model allows for precise control over the location and volume of the hematoma, making it particularly useful for studying the effect of secondary brain injury on cognitive function. For example, Jiang et al. (2019) confirmed that this model leads to the proliferation of microglia and astrocytes in the CA1, CA2, and CA3 regions, as well as the DG of the hippocampus. Additionally, levels of pro-inflammatory cytokines, such as tumor necrosis factor-α and interleukin-1β, were found to increase. These pathological changes result in impaired performance in behavioral tests, such as the Morris water maze and T-maze tests. Furthermore, some researchers believe that the cognitive dysfunction that occurs after intraventricular hemorrhage is linked to the accumulation of Aβ peptides and the exacerbation of neuroinflammation (Chen et al., 2011; Jiang et al., 2019).

#### Hypertensive intracerebral hemorrhage models

Hypertension-related models are used to simulate the pathological processes of hemorrhage following vascular rupture, utilizing genetic modifications (e.g., spontaneously hypertensive stroke-prone [SHRSP] rats) or drug-induced hypertension. These models effectively reflect the vascular lesions caused by long-term hypertension and its chronic effects on cognition. Chambers et al. (2022) reported that SHRSP rats exhibited a persistent decline in executive function and attention following ICH, which was associated with damage to the corticostriatal circuit. These models are particularly well-suited for exploring the mechanisms underlying the interaction between hypertension and cognitive impairment after ICH.

## Changes in Hippocampal Somatic Function in Poststroke Cognitive Impairment and the Related Mechanisms

Postischemic hippocampal injury refers to irreversible damage to the hippocampus caused by either persistent or transient insufficient cerebral blood flow perfusion. Post-ICH hippocampal injury is primarily characterized by distal secondary damage, which is related to ischemia resulting from physical compression of the hematoma and the diffusion of toxic blood products. The hippocampus is a metabolically active structure that is vulnerable to various metabolic states, such as hypoxia and hypoglycemia (Lana et al., 2020). The sensitivity of different cell types to hypoxia varies, with neurons being the most sensitive, followed by astrocytes, oligodendrocytes, and endothelial cells. Additionally, the susceptibility of different regions of the hippocampus to ischemic injury follows this order: CA1 and hilus > CA2 > CA3 > entorhinal gyrus. Notably, the CA1 region of the hippocampus is particularly sensitive to ischemia (Gao et al., 2015). The occurrence of ischemic lesions in hippocampal tissue involves multiple pathophysiological mechanisms, including excitatory amino acid toxicity, oxidative stress, immune inflammation, and apoptosis.

**Figure 4:**
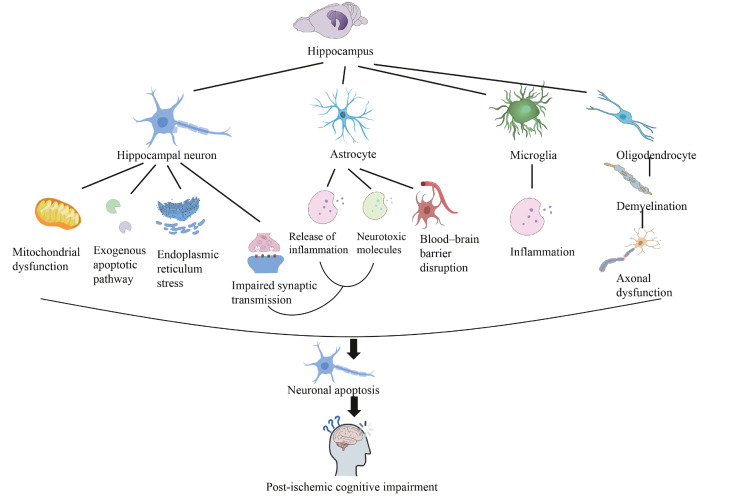
Pathological mechanisms of cognitive impairment after cerebral ischemia. This figure illustrates the contributions of neurons, astrocytes, microglia, and oligodendrocytes within the hippocampus to the development of cognitive deficits following ischemic events. The interplay among these cell types is examined to elucidate the pathological mechanisms underlying post-ischemic cognitive impairment.

The occurrence of PSCI is mainly associated with factors such as age, sex, educational level, primary hypertension, hyperlipidemia, and diabetes, as well as cerebrovascular damage, neurodegenerative changes in brain cells, inflammation, genetics, and molecular mechanisms (Wang et al., 2021). However, the pathogenesis of PSCI has not been fully elucidated. In this review, we will discuss the structural and functional changes in various cells involved in PSCI within the hippocampus and the related mechanisms. This analysis aims to provide new insights and approaches for further in-depth research into the pathogenesis and treatment of PSCI (**Figure 4**).

### Hippocampal neurons

Neurons are the primary nerve cells in the hippocampus and play crucial roles in processing and transmitting information. Hippocampal neurons can be divided into two main types: pyramidal cells and non-pyramidal cells. Pyramidal cells are the primary excitatory neurons in the hippocampus and are distributed mainly in the CA1, CA2, CA3, and DG regions. These cells have a typical pyramidal morphology and are rich in dendrites and axons for receiving and transmitting neural signals. Non-pyramidal cells are primarily inhibitory neurons, distributed in various regions of the hippocampus, and are mainly responsible for regulating the activity of pyramidal cells (Han, 1996; Soltesz and Losonczy, 2018).

Neuronal synapses are an important part of communication between neurons. Through information transmission at synapses, individuals can engage in various cognitive activities, including learning, memory, and other higher-level intellectual activities. Both learning and memory require multiple repeated stimuli that form and strengthen the neural circuits established by synapses between neurons. Stimulation triggers chemical or electrical changes in synapses, which in turn alters the connections between neurons (Martin et al., 2000). Neuronal synapses in the hippocampus are plastic and can directly participate in the integration and transmission of information in the nervous system, which forms the basis of memory formation and maintenance. When individuals receive information from other regions of the brain, it ultimately reaches the hippocampus through neuronal connections. In the hippocampus, this information is processed and stored as long-term memories. Studies have shown that when animals undergo certain learning or memory processes, the number of neurons in their hippocampus increases, as additional neurons are required to transmit and store information during memory formation (Lavenex and Banta Lavenex, 2013; Ramsaran et al., 2023).

Research has identified apoptosis as an important form of selective delayed neuronal death after cerebral ischemia, while necroptosis is recognized as a form of acute cell death following acute infarction (Gu et al., 2024). In the central nervous system (CNS), the hippocampus, striatum, and cerebral and cerebellar cortices are relatively sensitive to ischemia and are prone to delayed neuronal damage after ischemia. Cerebral ischemia can induce neuronal damage in the hippocampus, with neurons in the hippocampal CA1 region considered the most vulnerable. The duration of ischemia is the primary factor determining the severity of neuronal ischemic injury in the CA1 region (Gao et al., 2015). Some neurons in the hippocampus exhibit reduced volume, blurred boundaries, and disorganized cytoarchitecture, while some cells display cytoplasmic turbidity and vacuolation. Dendritic spine morphology in the CA1 region of the hippocampus changes considerably. Notably, within 12–24 hours post-reperfusion, the density of mushroom-shaped spines increases, whereas the density of swollen spines decreases. Concurrently, the density of short and thick dendritic spines decreases, but the density of slender dendritic spines increases (Merino-Serrais et al., 2024). Studies have shown that damage to neurons in the hippocampus affects individuals’ cognitive abilities (Zhao et al., 2014; Dong et al., 2022). When individuals experience cognitive impairment, the hippocampus presents different states, indicating that hippocampal neurons are closely related to cognitive function. Research on cognition in mice has revealed that hippocampal neurogenesis plays an important role in improving cognitive function (Sun et al., 2020a), and that a decrease in cognitive ability is related to damage to hippocampal neurons.

Apoptosis is a tightly regulated signaling cascade involving gene activation, enzymatic cascades, and intracellular signaling pathways. The occurrence of apoptosis is a complex process, and multiple pathways can independently trigger apoptosis. There are three main types of apoptotic signal transduction in hippocampal neurons: intrinsic (mitochondria-mediated apoptosis), extrinsic (death receptor-mediated apoptosis), and endoplasmic reticulum-mediated apoptosis (Fricker et al., 2018). Apoptosis is initiated either when cell surface death receptors such as Fas bind to their corresponding ligands or when pro-apoptotic proteins of the B-cell lymphoma 2 (Bcl-2) family increase the permeability of the mitochondrial outer membrane. Both of these pathways activate the caspase protease family, ultimately resulting in cell disintegration (Green and Llambi, 2015). The endoplasmic reticulum (ER) plays a role in reducing protein synthesis in cells, facilitating the proper folding of proteins, and maintaining Ca^2+^ homeostasis. Nevertheless, an excessive stress response in the ER can trigger apoptotic signals within cells and accelerate the process of apoptosis.

The apoptosis of hippocampal neurons leads to a reduction in the number of synapses, resulting in abnormal synaptic function and affecting the stability and efficiency of synaptic transmission. These effects may manifest as either weaker or stronger synaptic transmission, which in turn impacts the transfer of information between neurons. Additionally, neuronal apoptosis may be accompanied by changes in synaptic morphology, such as a smaller presynaptic membrane and a wider synaptic cleft (Ge et al., 2020). These morphological changes can affect the efficiency of synaptic transmission and the performance of synaptic plasticity. The apoptosis of hippocampal neurons and changes in synapses may jointly affect hippocampal function, leading to impairments in higher neural activities, such as memory and learning. For example, apoptosis in hippocampal neurons could inhibit synaptic plasticity, thereby affecting learning and memory.

The pathological mechanism of cognitive ICH is complex and involves a dynamic imbalance among hippocampal neurons, synaptic plasticity, and the glial cell network.

#### Mitochondria-mediated intrinsic apoptosis and mitochondrial dysfunction

As key energy-producing organelles, mitochondria generate more than 90% of neuronal ATP through oxidative phosphorylation, taking glucose and oxygen as substrates. Following cerebral ischemia, impaired oxygen and glucose delivery to brain tissue triggers mitochondrial dysfunction, finally leading to neuronal death and functional impairment (Chouchani et al., 2014; Venna et al., 2014). This central role positions mitochondria as pivotal regulators of cell death mechanisms.

The Bcl-2 family of proteins plays a crucial role in regulating endogenous apoptotic signaling. These proteins govern the permeability of the mitochondrial outer membrane, ultimately triggering the activation of the caspase cascade, which results in cell apoptosis and death. This gene family consists of antiapoptotic proteins such as Bcl-2 and Bcl-xL, along with proapoptotic members such as Bax and Bad. These proteins are located in the outer mitochondrial membrane, endoplasmic reticulum, and perinuclear areas. Antiapoptotic proteins, such as Bcl-2, sequester their proapoptotic counterparts, thereby inhibiting apoptosis (Fricker et al., 2018; Zhang et al., 2022a). Cytochrome c is a potent proapoptotic factor that binds to Apaf-1 and procaspase-9 to promote apoptosis. This complex allows for the autocatalytic activation of caspase-9, followed by the cleavage of the protein caspase-3, thus achieving endogenous apoptosis (Mao et al., 2022). Understanding these molecules highlights their therapeutic potential for alleviating cognitive deficits after ischemia by targeting apoptotic pathways.

Zhang et al. (2023a) reported that after BCAO-induced cerebral ischemia in rats, levels of the proapoptotic proteins Bax and caspase-3 in the hippocampus increased, while levels of the antiapoptotic protein Bcl-2 decreased. This imbalance led to a decline in spatial navigation ability and in learning and memory functions. In contrast, edaravone dexborneol treatment reduced Bax and caspase-3 levels, inhibited apoptosis in the CA1 region of the hippocampus, promoted neuronal survival, and improved cognitive performance. Edaravone dexborneol is a new neuroprotective agent composed of edaravone and right borneol.

Complementary evidence reveals that TAT-LBD-Ngn2 can reduce cerebral I/R injury by inhibiting neuronal degeneration and apoptosis in the hippocampus. Moreover, it can improve short-term cognitive performance by alleviating caspase-dependent and mitochondrial apoptosis pathways through brain-derived neurotrophic factor-tropomyosin receptor kinase B (BDNF-TrkB) signaling (Zhao et al., 2018; Feng et al., 2023). Alternatively, targeted treatment with the mitochondrial antioxidant MitoQ (Ibrahim et al., 2023) also plays a role. After cerebral ischemia induced by BCAO for 45 minutes, treatment with MitoQ can alleviate neuronal damage and mitochondrial oxidative stress in rat hippocampal neurons. It can also restore mitochondrial biogenesis-related proteins PGC-1α, TEAM, and NRF-1 in hippocampal neurons to normal levels, helping to stabilize mitochondrial function after cerebral ischemia, reduce neuronal apoptosis, and improve learning and memory function.

Autophagy plays a protective role in the early stages of ischemia by eliminating damaged mitochondria. However, if it is continuously activated, it can deplete lysosomes and increase the severity of neuronal apoptosis. Clearly, autophagy has a dual role in neuronal death: moderate activation is protective, while excessive activation is detrimental. In the early stages, autophagy selectively removes damaged mitochondria through the lysosomal pathway, a process known as mitophagy, preventing the accumulation of ROS and oxidative stress (Nixon and Yang, 2012; Gupta et al., 2021). Autophagy also alleviates ER stress by clearing misfolded protein aggregates such as Aβand α-synuclein. AMP-activated protein kinase activation inhibits mechanistic target of rapamycin complex 1 and promotes the initiation of autophagy by removing damaged organelles. However, if ischemia persists, it can cause problems in lysosomal acidification and block autophagic flow, resulting in the accumulation of autophagosomes and ultimately cell death (Mizushima and Levine, 2020). Autophagy can also maintain the survival of neurons by degrading proapoptotic factors such as Bax and caspase-8. Ischemic preconditioning can enhance autophagic flow and reduce the degree of apoptosis in penumbral neurons.

However, the continuous formation of autophagosomes can lead to excessive degradation of cytoplasmic components, triggering lysosomal membrane rupture. This increases the LC3-II/I ratio and causes the release of cathepsin B. If autophagy is activated for a prolonged period, ATP stores may be depleted, and the mitochondrial membrane potential may decrease. When beclin1 and Bcl-2 are separated, both the autophagy and mitochondrial apoptosis pathways are activated (Deng et al., 2021). JNK phosphorylates Bcl-2, which enhances the interaction between autophagy and apoptosis (Liu et al., 2012).

In summary, therapeutic strategies targeting mitochondria-mediated intrinsic apoptosis through the inhibition of apoptotic cascades or the restoration of mitochondrial homeostasis have shown promise in preserving hippocampal neuron integrity and alleviating postischemic cognitive dysfunction.

#### Exogenous apoptosis pathways in neurons

Neuronal apoptosis is regulated through both intrinsic (mitochondrial) and extrinsic (death receptor-mediated) pathways. Following cerebral ischemia, proapoptotic signaling involves the Bcl-2 family-mediated activation of caspase cascades (Zhang et al., 2022a). Caspases exhibit functional specialization, with caspase-3 (the executioner) and caspase-9 (the initiator) being central to neuronal apoptosis.

Members of the tumor necrosis factor (TNF) superfamily, such as TNF, Fas ligand (FasL), and TNF-related apoptosis-inducing ligand (Fricker et al., 2018; Zhang et al., 2022a), bind to cognate death receptors, such as TNF receptor (TNFR), to induce apoptosis. FasL and Fas participate in the recruitment of Fas-associated death domain to form the death-inducing signaling complex, which also promotes the aggregation and activation of procaspase-8 and the cleavage of downstream caspase-3/7. This cascade of reactions eventually leads to cognitive impairment.

Notably, activation of the sonic hedgehog pathway exerts a neuroprotective effect through the upregulation of glioma-associated oncogene 1 (Gli-1)-dependent Bcl-2 expression and the inhibition of caspase-3 activity, thereby rescuing cognitive deficits after ischemia (Wang et al., 2023). The neurotrophic factor/tropomyosin receptor kinase (Trk) signaling axis (Li et al., 2021b) is activated by BDNF binding to TrkB, which subsequently binds to mitogen-activated protein kinase/extracellular signal-regulated kinase (MEK3)/phosphorylated extracellular signal-regulated kinase 1/2 (p-ERK1/2)/cAMP response element-binding protein (CREB) to downregulate c-Jun N-terminal kinase 1 (JNK1)/c-Fos, thereby promoting hippocampal neuron survival and neurogenesis. Studies have confirmed the importance of the phosphoinositide 3-kinase (PI3K)/protein kinase B (Akt) pathway, which is a cascade mediated by G protein-coupled receptors that regulates proliferation, differentiation, and migration (Sun et al., 2020b; Fan et al., 2021). Sun et al. (2022) demonstrated that the activation of the PI3K/Akt and mitogen-activated protein kinase/extracellular signal-regulated kinase (MAPK/ERK) pathways together drives hippocampal neurogenesis and that hydrogen sulfide can promote neuronal survival through Akt phosphorylation and apoptosis signal-regulating kinase 1/Jun N-terminal kinase 3 (ASK1/JNK3) inhibition (Wen et al., 2014).

Pyroptosis represents a proinflammatory form of programmed cell death initiated by NLRP3 inflammasome activation and subsequent caspase-1 cleavage. Caspase-1 executes pyroptosis via gasdermin D (GSDMD), which forms plasma membrane pores to mediate lytic cell death. Dong et al. (2022) demonstrated that cerebral ischemia-induced hippocampal upregulation of pyroptotic markers (i.e., caspase-1, NLRP3, GSDMD, apoptosis-associated speck-like protein containing a CARD [ASC], interleukin [IL]-1β, and IL-18) directly linked pyroptosis to short- and long-term postischemic memory deficits.

After IS, primary brain injury generates large amounts of ROS in mitochondria, especially superoxide (O_2_^–^) and hydroxyl radicals (^–^OH). Excessive ROS can cause oxidative damage to neurons and lead to various types of neuronal cell death, such as ferroptosis (Xie et al., 2016). Moreover, the continuous production of ROS can also trigger neuroinflammation, which is even more damaging to the brain. This neuroinflammation increases the permeability of the BBB, allowing immune cells to infiltrate the ischemic brain. Neuroinflammation produces cytokines, such as interleukin IL-1 and IL-6, that worsen the inflammatory response. In addition, excessive ROS production can increase the size of infarctions (Simpson and Oliver, 2020; Zhao et al., 2021c). After an IS or traumatic brain injury, the antioxidant mechanisms in neurons are unable to resist the oxidation of nucleic acids, lipids, and proteins. Excessive ROS-induced damage to subcellular structures in neurons, especially mitochondria, can trigger an inflammatory response. Yan et al. (2021a) further identified dimethyl fumarate as a potent modulator of the nuclear factor erythroid 2-related factor 2 (NRF2)/ARE/nuclear factor kappa-light-chain-enhancer of activated B cells (NF-κB) axis that suppressed hippocampal tumour necrosis factor alpha (TNF-α)/IL-1β/IL-6 expression, inhibited ferroptosis, and rescued postischemic cognitive deficits in rats.

Collectively, some signalling pathways can regulate cell proliferation, differentiation and migration; promote neuronal regeneration and repair; improve cerebral ischemic lesions, and reduce learning and memory impairment after ischemia.

#### Endoplasmic reticulum–mediated apoptosis

The ER is a major processing plant for protein synthesis and an important Ca^2+^ reservoir. Consequently, the ER is crucial for preserving cellular Ca^2+^ homeostasis, as well as protein synthesis and processing (Marchi et al., 2018). Hippocampal neurons may suffer from ER stress due to factors such as ischemia and hypoxia. This stressed state can lead to impaired ER function, which in turn affects protein synthesis and folding, resulting in the production of misfolded proteins. These proteins accumulate in the ER and further aggravate ER stress.

Studies have shown that BCAO induces a decline in spatial learning in rats and increased expression of the ER stress-related proteins PERK, elF2α, IRE1, p-IRE1, p-PERK, p-eif2a, XBP1, XBP1, ATF6, GRP78, CHOP and caspase-1 (Niu et al., 2019; Meng et al., 2024). After stroke, the balance of Ca^2+^ within hippocampal neurons may be disrupted, resulting in a massive release of Ca^2+^ from the ER into the cytoplasm. This Ca^2+^ imbalance triggers a series of downstream responses, including the activation of Ca^2+^-dependent enzymes and signalling pathways that ultimately promote apoptosis. ER stress activates the mitochondrial apoptosis pathway by releasing stored Ca^2+^. This results in increased permeability of the mitochondrial membrane and the release of cytochrome c (Han et al., 2021; Jiang et al., 2023). Moreover, the generation of ROS induced by ER stress further exacerbates mitochondrial dysfunction, forming a vicious cycle (Sano and Reed, 2013). Hippocampal neurons may be damaged by oxidative stress after cerebral ischemia. Oxidative stress generates large amounts of ROS that attack proteins and other molecules in the ER, leading to impaired ER function and apoptosis.

#### Hippocampal neurons after hemorrhagic stroke: Aplasia and the regulation of apoptosis

The imbalance between damage to and regeneration of hippocampal neurons is the core mechanism of cognitive impairment. Zhang et al. (2024b) have reported that iron ions abnormally accumulate in the hippocampus after ICH, triggering excessive activation of NSCs through the ROS/Itga3 signaling axis. This activation leads to depletion of the NSC pool and reduced neurogenesis. As a novel mode of cell death, ferroptosis exacerbates neuronal damage through lipid peroxidation, while bovine serum albumin-stabilized selenium nanoparticles inhibit ferroptosis, improve mitochondrial function, and restore cognition by activating the Nrf2/glutathione peroxidase 4 (GPX4) axis (Zhou et al., 2021; Li et al., 2024b). Additionally, the upregulation of PTEN signaling inhibits the AKT/mTOR pathway and hinders NSC proliferation, whereas tetrahydrofolate promotes neurogenesis by antagonizing phosphatase and tensin homolog (PTEN) (Zhang et al., 2024c). Lithium chloride further improves cognitive function by inhibiting glycogen synthase kinase-3β (GSK-3β), stabilizing β-catenin, and reducing neuronal apoptosis (Liu et al., 2018). The interaction between iron homeostasis imbalance and the PTEN/AKT/mTOR pathway may represent a key target for addressing neuronal aplasia, and targeting iron chelation or PTEN inhibition may provide a new direction for the treatment of this condition.

#### Synaptic plasticity

Neuronal apoptosis not only directly reduces the number of synapses through cell death but also indirectly affects synaptic protein synthesis and plasticity by disrupting the mitochondrial energy supply and ER protein homeostasis. Neurons are functionally interconnected, and the key structure facilitating information transmission between them is the synapse. Synapses are the most sensitive and plastic structures in the CNS and directly participate in the integration and transmission of information within the nervous system (Bin Ibrahim et al., 2022). Synaptic efficacy depends on the coordination of presynaptic and postsynaptic structural integrity and plasticity. Activity-dependent changes in synaptic connection strength in the hippocampus are fundamental to memory formation and maintenance (Buffington et al., 2014; Magee and Grienberger, 2020). Synaptic function is influenced by changes in synaptic density and protein composition. Cerebral ischemia reduces dendritic spine density and functional synapses, accompanied by decreased expression of postsynaptic receptor subunits (e.g., NR2A and NR2B) and synaptic proteins such as postsynaptic density protein 95 (PSD95). However, remote ischemic preconditioning has been shown to mitigate these impairments in synaptic plasticity and alleviate BCAO-induced cognitive deficits by downregulating miR-218a-5p expression (Li et al., 2023b).

LTP refers to the sustained strengthening of synaptic responses and the subsequent enhancement of interneuronal signaling. Hippocampal interneurons play an important role in the LTP mechanism. When hippocampal LTP is inhibited, cognitive impairment may occur (Lu et al., 2014). In addition to BDNF, environmental enrichment enhances LTP in the hippocampus through α7-nicotinic acetylcholine receptors, which are cholinergic receptors abundantly expressed in the hippocampus and are particularly critical for learning and memory (Yuan et al., 2020). LTD is another important form of synaptic plasticity that may be closely related to learning. The induction of LTD typically requires the activation of N-methyl-D-aspartate (NMDA) receptors followed by the endocytosis of α-amino-3-hydroxy-5-methyl-4-isoxazolepropionic acid receptors. This reduced efficiency of synaptic transmission is thought to underlie some types of learning (Bin Ibrahim et al., 2022). LTD is involved in the regulation of learning processes by altering synaptic transmission efficiency and affecting the strength of connections between neurons.

Cerebral ischemia induces decreases in the levels of synaptic markers, such as synaptophysin (SYN) and PSD95, which are associated with cognitive impairment. In the hippocampus, the constitutively active α7-nAChR/PI3K pathway (Chen et al., 2020; Fan et al., 2021) can enhance synaptic plasticity and play a neuroprotective role by restoring the expression of SYN and PSD95 after middle cerebral artery occlusion/reperfusion (MCAO/R). Hippocampal neurons and medial prefrontal cortical neurons form functional synapses, allowing the hippocampus to directly regulate prefrontal activity. After MCAO/R, the level of synaptosomal-associated protein 29 decreases in neurons following ischemia, disrupting synaptic homeostasis and neural circuits (Yan et al., 2021b). This indicates that synaptosomal-associated protein 29 deficiency is a potential factor leading to cognitive impairment.

Cerebral ischemia also alters the dendritic morphology of hippocampal neurons, resulting in reduced dendritic complexity, shortened dendrites, and fewer dendritic spines in CA1 pyramidal neurons—changes associated with learning and memory deficits (Sarkala et al., 2023). Zhang et al. (2019) reported decreased levels of growth associated protein 43 (GAP-43), synaptophysin (SYP), SYN, and PSD-95 following four-vessel occlusion-induced cerebral ischemia. Cdh1 overexpression rescued hippocampal synapse and dendritic spine loss, prevented dendritic network disorganization, and improved post-ischemic cognitive deficits by modulating the expression of synaptic and axonal growth-related proteins.

Synaptic loss is an important feature of cognitive impairment after intracerebral hemorrhage (ICH). Complement components such as C3 label synapses after activation, leading to a decrease in synaptic density through microglial phagocytosis. In contrast, the targeted complement inhibitor B4Crry reduces synaptic loss and improves cognition (Alawieh et al., 2020). In addition, MFGE8 activates PI3K/mTOR through the integrin β3/Akt signalling pathway, which promotes the expression of synaptic proteins (e.g., PSD95) and neurite outgrowth and restores synaptic function (Li et al., 2024c). The dynamic balance between the complement system and the synaptic repair mechanism may determine cognitive outcomes, and the combination of complement inhibition and synaptic regeneration strategies may have greater therapeutic potential.

### Astrocytes

Astrocytes, the most abundant glial cell population in the CNS, perform a variety of supportive functions, including nutrient transport, BBB formation, maintenance of ionic homeostasis, and tissue repair following injury. After cerebral ischemia, astrocytes detect neuronal damage and undergo a phenotypic transition from a quiescent to a reactive state, exerting dual regulatory effects on synaptic integrity and neuronal survival (Yamagata, 2021). When ischemic damage to astrocytes exceeds a critical threshold, they overexpress and secrete pro-inflammatory mediators, such as IL-6, TNF-α, IL-1α, IL-1β, and interferon-γ, as well as free radicals and neurotoxic molecules (Patabendige et al., 2021). This response can precipitate synaptic dysfunction and neuronal impairment, contributing to cognitive deficits.

Local neuronal activity can modulate the interactions between astrocytes and synapses through various mechanisms, including the secretion of glial transmitters, regulation of synaptic proteins in an activity-dependent manner, and contact-dependent signaling pathways. Conversely, the specific responses of astrocytes can orchestrate two-way communication between neurons and glial cells, thereby influencing synaptic communication within the adult hippocampus (Wang et al., 2022a). Astrocytes closely regulate the remodeling of synaptic structures and long-term functional plasticity. Abnormal astrocytic signaling can disrupt homeostasis at both the synaptic and network levels, ultimately leading to cognitive impairment (Santello et al., 2019). Increasing evidence suggests that activated astrocytes can enhance synaptic transmission and promote memory consolidation (Adamsky et al., 2018). Reactive astrocytes can differentiate into pro-inflammatory A1 subtypes or reparative A2 subtypes. A1-type astrocytes release complement component C3, which directly induces synaptic phagocytosis and contributes to cognitive decline. In contrast, A2 astrocytes promote synapse regeneration by secreting thrombomodulin (Liddelow et al., 2017). Fu et al. (2023) reported that in a 4VO model of transient cerebral ischemia, knocking down lysosome-associated membrane protein type 2A in rat astrocytes decreased astrocytic activation. Furthermore, this intervention increased the number of surviving pyramidal neurons in the hippocampal CA1 region and alleviated synaptic damage induced by ischemia, resulting in improved spatial learning and memory deficits.

Astrocytes also metabolically support neurons through the astrocyte-neuron lactate shuttle, converting glycolytic pyruvate to lactate for neuronal energy metabolism. Ischemia upregulates PFKFB3, the rate-limiting enzyme of astrocytic glycolysis, enhancing lactic acid production while suppressing glutamine synthetase activity. This pathological shift reduces glutamate clearance capacity and exacerbates excitotoxic neuronal damage. Kang et al. (2023a) demonstrated that the astrocyte-neuron lactate shuttle-mediated lactate supply preserves neuronal viability, mitigates oxidative stress and microtubule destabilization, and alleviates neurological and cognitive dysfunction, even under conditions of pyruvate kinase M2 deficiency.

The interplay between astrocytes and endothelial cells, mediated by secreted factors, inflammatory signaling pathways, and metabolic coupling, plays a critical role in maintaining the integrity and functionality of the BBB. Following IS, the synergistic dysfunction of these two cell types contributes to BBB breakdown, neuroinflammation, and energy metabolism disruption, ultimately leading to cognitive deficits. After cerebral ischemia, vascular endothelial growth factor (VEGF) secreted by astrocytes activates the Src kinase signaling pathway in endothelial cells, resulting in the phosphorylation and degradation of occludin and claudin-5, which increases the permeability of the BBB. Activated astrocytes and endothelial cells release pro-inflammatory factors such as IL-6 and TNF-α, which activate the NF-κB pathway in endothelial cells, inducing the expression of adhesion molecules (such as intercellular adhesion molecule-1) and promoting the infiltration of peripheral white blood cells and neuroinflammation (Archie et al., 2021). Post-ischemic swelling of astrocytic endfeet (aquaporin-4 polarity disruption) triggers BBB leakage, enabling peripheral inflammatory cells (e.g., neutrophils and monocytes) and pro-inflammatory cytokines (e.g., IL-1β and TNF-α) to infiltrate the brain parenchyma, a process that drives chronic neuroinflammation and selective hippocampal/cortical neuron loss (Sweeney et al., 2018). Activated astrocytes secrete matrix metalloproteinase-9, which degrades basement membrane collagen IV and laminin, compromising BBB structural integrity and exacerbating vasogenic edema and secondary neuronal injury (Rosenberg, 2009). BBB breakdown permits peripheral glutamate precursors to enter the CNS, while astrocytic GLT-1 dysfunction impairs synaptic glutamate clearance; these dual mechanisms ultimately lead to excitotoxic neuronal death. Concurrent disruption of the BBB and loss of astrocytic aquaporin-4 polarity impair the function of the glymphatic system, leading to reduced clearance of cerebral Aβand tau proteins, and accelerating the pathogenesis of vascular dementia (Iliff et al., 2012). Decreased function of endothelial glucose transporters and astrocyte lactate shuttles (e.g., MCT1/2) results in insufficient energy supply to neurons, further exacerbating cognitive decline (Moskowitz et al., 2010).

Collectively, astrocytic dysfunction provokes neuroinflammation, synaptic-neuronal damage, and BBB breakdown, which are key pathological drivers of PSCI. Thus, targeting pathological astrocyte activation may be a promising therapeutic strategy for mitigating cognitive deficits following IS.

Astrocytes also play an important role in ferroptosis. Elevated ROS levels after ICH trigger lipid peroxidation, whereas bovine serum albumin-stabilized selenium nanoparticles increase GPX4 expression by activating Nrf2, inhibiting ferroptosis and protecting astrocyte function (Li et al., 2024b). In addition, total flavonoids in camellia sinensis can attenuate astrocyte apoptosis by reducing the malondialdehyde level and the Bax/Bcl-2 ratio (Lu and Wen, 2020). The antioxidant capacity of astrocytes is key to maintaining the stability of the hippocampal microenvironment, and targeting the Nrf2/GPX4 axis may enhance its neuroprotective effect.

### Microglia

Microglia exert context-dependent dual effects in cerebral ischemia: moderate activation facilitates the phagocytic clearance of cellular debris, whereas overactivation perpetuates neuroinflammatory cascades. These cells engage in dynamic crosstalk with, and are reciprocally modulated by, neurons, astrocytes, oligodendrocytes, and endothelial cells throughout the progression of IS (Qin et al., 2019; Wang et al., 2022b). The M1 phenotype compromises CNS tissue repair via the secretion of pro-inflammatory mediators, whereas the M2 phenotype promotes tissue regeneration through the production of anti-inflammatory cytokines.

On one hand, microglia can actively regulate neurogenesis, thereby contributing to cognitive improvement. They achieve this by guiding cell migration and affecting synaptic activity through the regulation of the quantity of functional synapses in the CNS. On the other hand, inflammation mediated by activated microglia is harmful to both neurogenesis and the survival of newborn neurons, ultimately resulting in dysfunction (Xiong et al., 2016). In addition to M1/M2 microglia, Mhem microglia can degrade heme through the heme oxygenase-1 pathway and reduce oxidative damage in the hippocampus; thus, they may constitute a new target for intervention. Microglia can recognize the synaptic marker C3 through complement C1q and mediate the elimination of dendritic spine abnormalities in the CA1 region. Additionally, inhibition of C3aR can restore synaptic density.

Microglia may participate in microglial polarization and neuroinflammation-related pathways associated with cognitive impairment after cerebral ischemia (Zeng et al., 2024). These pathways include the microglial Toll-like receptor (TLR) signaling pathway, the Janus kinase-signal transducer and activator of transcription signaling pathway, the NF-κB signaling pathway, and the P2 receptor family signaling pathway. Song et al. (2022) reported that the transgenic deletion of the microglial pH-regulating protein sodium/hydrogen exchanger 1 shifted microglial metabolism from glycolysis to oxidative phosphorylation, resulting in increased ATP production. These microglia also exhibited enhanced phagocytic activity, synaptic remodeling, and myelin repair capacity. These metabolic alterations were followed by improved cognitive recovery after stroke. Xia et al. (2022) reported that TRIM45 knockdown specifically in mouse microglia can inhibit excessive activation of microglia after MCAO-induced cerebral ischemia injury, limit the activation of NF-κB pro-inflammatory signals, and considerably improve neuronal activity as well as learning and directed memory abilities.

Microglial activation is key to chronic inflammation after ICH. The activation of complement-dependent synaptic phagocytosis, namely, C3 opsonization, and the Fc receptor/DAP12 pathway can cause synaptic loss, but the complement inhibitor B4Crry can inhibit microglial proliferation (Alawieh et al., 2020). Additionally, increased intracranial pressure combined with ICH can activate the classical complement pathway, increasing the severity of microglia-mediated synaptic pruning (Puglisi et al., 2023). PLX3397 can reduce the number of microglia by inhibiting colony-stimulating factor 1 receptor, thereby significantly improving cognitive function (Shi et al., 2019). Microglia play dual roles, as they can be involved in protective clearance and pathological phagocytosis, which requires precise regulation. Targeting the complement or colony-stimulating factor 1 receptor signaling pathway may optimize the therapeutic window.

### Oligodendrocytes

In the CNS, the myelin sheath is formed by oligodendrocytes (OLs), which encircle the axons of neurons in segments, providing the structural basis for the rapid conduction of nerve impulses. OL injury can lead to demyelination and disorders of myelination, greatly affecting axon function, structure, metabolism, and survival. OL injury is induced following cerebral ischemia, resulting in demyelination, axon instability, and white matter loss, which adversely affect neuronal viability and lead to long-term neurological dysfunction (Huang et al., 2023). The plasticity of synapses has been recognized, and the plasticity of OLs, which produce the myelin sheath, plays a crucial role in learning and memory. OLs influence multiple aspects of neuronal function, including axonal conductance, synaptic efficacy, and excitability (Xin and Chan, 2020).

OLs may also affect neuronal plasticity at the synaptic level. Olig2 deficiency in OLs leads to myelin dysplasia, which can directly impair neuronal functional development, disrupt synaptic structure, and cause functional defects. Conversely, M1R depletion in oligodendrocyte precursor cells (OPCs) improves myelination and subsequently promotes functional recovery after chronic hypoxia (Wang et al., 2018a). After BBB leakage, astrocytes release ROS and proinflammatory factors such as interferon-γ, which inhibit OPC differentiation and myelin repair, leading to leukoaraiosis and cognitive decline (Domingues et al., 2016).

The CX3CR1–CX3CL1 axis critically regulates microglial activation. A preclinical study has demonstrated that CX3CR1 downregulation suppresses microglial hyperactivation following 60-minute BCAO-induced ischemia, concomitantly attenuating systemic inflammation (e.g., reduced serum IL-1β, IL-6, and TNF-α levels) and reversing striatal, cortical, and hippocampal demyelination (Du et al., 2020). This therapeutic approach promotes the differentiation of OPCs into mature myelinating OLs, preserves hippocampal neuron integrity, and ameliorates postischemic depression-like phenotypes and cognitive deficits in murine models.

Although few direct studies exist, white matter damage after ICH may affect oligodendrocyte function. Diffusion tensor imaging (DTI) has revealed decreased fractional anisotropy in the hippocampus of patients with ICH, suggesting white matter microstructural disruption (Song et al., 2020). In a model of germinal matrix–intraventricular hemorrhage, transcriptome analysis revealed the downregulation of neurodevelopmental pathways, possibly involving oligodendrocyte myelination disorders (Qin et al., 2025). OL-mediated myelin repair may constitute a future research direction, and the combination of DTI and molecular regulation may elucidate its potential mechanisms.

### Neurotransmitters

Neurotransmitters are chemical substances released by nerve terminals, functioning as messengers during chemical synaptic transmission. They act upon receptors located on the membranes of innervated neurons or effector cells to facilitate information transmission. Torres-López et al. (2023) have reported that various neurotransmitters change after PSCI. After permanent MCAO, mice exhibited episodic memory impairment and increased levels of γ-aminobutyric acid (GABA) in the hippocampus. Over-activated GABA neurotransmission leads to abnormal morphology in newborn neurons and subsequent hippocampus-dependent memory deficits in mice.

Cognitive dysfunction is also linked to cholinergic neuron degeneration. Four weeks after BCAO, hippocampal choline acetyltransferase and acetylcholine (ACh) levels increase, while acetylcholinesterase activity declines (Du et al., 2017). Acetylcholinesterase catalyzes the degradation of synaptic ACh to terminate cholinergic signaling, whereas choline acetyltransferase synthesizes ACh and serves as a cholinergic marker. Cerebral hypoperfusion-induced oxidative stress primarily underlies cholinergic dysfunction, implicating redox imbalance in the disruption of central cholinergic pathways (Bin Ibrahim et al., 2022).

Additionally, cerebral ischemia reduces hippocampal glutamate content but enhances neuronal glutamate uptake via excitatory amino acid transporter 2. This paradoxical increase in glutamate excitotoxicity exacerbates neuronal damage and cognitive deficits (Yeh et al., 2005; Kulbe et al., 2014).

## Effective Measures to Improve Cognitive Impairment after Poststroke Cognitive Impairment

Hippocampal injury is the primary pathological basis of cognitive decline, particularly memory deficits in PSCI. Effective interventions targeting hippocampal injury, along with the promotion of nerve repair and functional remodeling, are essential for improving the prognosis of patients with PSCI. Therefore, this review explores and summarizes measures that can effectively enhance hippocampal recovery in patients with PSCI, focusing on rehabilitative interventions including Chinese herbal medicines and their active ingredients, and stem cell therapies.

### Neuroprotective agents

The core pathological mechanism of PSCI involves a complex cascade of reactions, including neuroinflammatory activation, oxidative stress bursts, and mitochondrial dysfunction-induced neuronal apoptosis. The research and development of targeted neuroprotective drugs has become an important strategy. At present, a variety of neuroprotective drugs have been widely explored in this field.

The inflammatory response emerges during the early acute stage of stroke and persists throughout the injury process. Its intensity is associated with the severity and prognosis of the condition. Therefore, understanding the mechanism of post-stroke inflammation and its regulation is a promising treatment strategy for stroke. Valproic acid is an anticonvulsant and mood-stabilizing drug with anti-inflammatory and neuroprotective effects (Xuan et al., 2012). It ameliorates severe deficits in spatial cognitive performance. Post-injury treatment with valproic acid remrakably inhibits hippocampal CA1 microglial activation, reduces the number of microglia, and inhibits both histone deacetylase activity and the induction of heat shock proteins. Dimethyl fumarate is an FDA-approved drug for the treatment of multiple sclerosis and plays a protective role in various neurological diseases (Yan et al., 2021a). It exerts antioxidative stress, antiferroptotic, and anti-inflammatory effects through the NRF2/ARE/NF-κB signaling pathway, reducing damage to hippocampal neurons in rats after BCAO.

Oxidative stress, a common pathway for cognitive decline, drives the development of innovative antioxidant intervention strategies. Some antioxidants neutralize ROS and reactive nitrogen species by activating the endogenous antioxidant pathway (i.e., the Nrf2/KEAP1-ARE axis), which activates the Nrf2 transcription factor and upregulates the expression of enzymes including HO-1, SOD, and GPx. Edaravone combined with right borneol can increase Nrf2 nuclear translocation and reduce neuronal apoptosis after ischemia-reperfusion (Xu et al., 2022b). Antioxidants also regulate the GPX4/SLC7A11 pathway by inhibiting ferroptosis to maintain glutathione homeostasis and inhibit lipid peroxidation (Zheng et al., 2024). Rehmannioside A upregulates SLC7A11/GPX4 and attenuates hippocampal ferroptosis and cognitive impairment following ischemia (Fu et al., 2022). The Elabela peptide protects neurons after ischemia-reperfusion by inhibiting ferroptosis through HO-1 (Xu et al., 2023). Targeting ferroptosis is an emerging strategy, particularly for treating PSCI. Additionally, antioxidants ameliorate mitochondrial dysfunction to protect against cognitive decline. MitoQ, an antioxidant targeting mitochondria, reduces ROS and improves post-ischemic memory by increasing complex I activity (Ibrahim et al., 2023). β-Hydroxybutyric acid, a regulator of energy metabolism, replaces energy substrates and restores mitochondrial respiratory chain function, thereby alleviating executive function and working memory impairment (Gureev et al., 2024).

Apoptosis is a primary pathological link between cognitive impairment caused by ischemic brain injury and neurodegenerative diseases. Inhibiting the pathological apoptotic cascade is a key strategy to protect neurons and maintain neural network function, thereby preventing or improving cognitive decline. Cognitive improvement is directly related to an increased number of surviving neurons and reduced damage to key cognitive regions such as the hippocampus. Oxidative stress is one of the major triggers of apoptosis following cerebral ischemia. Substances with strong antioxidant capacity (Zhang et al., 2024a) can directly remove ROS, eliminate important triggers for apoptosis, protect the structural and functional integrity of neurons, and preserve cognitive function. The inhibition of apoptosis is crucial for protecting neurons, glial cells, and synaptic connections, thereby maintaining or restoring cognitive function. By targeting the endogenous mitochondrial apoptosis pathway, the death receptor/inflammatory pathway, and the activation of cell survival/autophagic pathways, various interventions based on direct antioxidant and multitarget synergistic strategies have demonstrated clear anti-apoptotic effects and the potential to improve cognitive impairment in preclinical models. These anti-apoptotic mechanisms ultimately protect or enhance cognitive functions such as learning and memory by reducing neuron loss in key brain areas (e.g., the hippocampus and cortex), maintaining white matter/myelin integrity, and supporting synaptic plasticity. The aggravated inflammatory response after cerebral ischemia serves as an important inducer and amplifier of apoptosis. Targeting death receptors such as Fas and TNFR1, as well as inflammation-related signals such as TNF-α, IL-1β, and NF-κB, can yield both anti-inflammatory and anti-apoptotic effects. This dual protective mechanism jointly promotes the repair of the neural network and the recovery of cognitive function (Gu et al., 2016; Yang et al., 2022).

First, the endogenous or mitochondrial apoptosis pathway is the core executor of apoptosis, and targeting its key regulatory points, such as Bcl-2 family members, mitochondrial permeability transition pores, and caspases, can effectively block the apoptotic process. VX-765 is a potent small-molecule caspase-1 inhibitor that has shown promise in preventing CNS diseases. Treatment with VX-765 may alleviate Aβ deposition, secondary degeneration, pyroptosis, and apoptosis in the hippocampus, ultimately improving cognitive decline following focal cerebral ischemia by inhibiting the activation of the MAPK and NF-κB pathways (Dong et al., 2022). Additionally, receptor-mediated activation of erythropoietin can significantly upregulate the expression of anti-apoptotic proteins such as Bcl-2 and Bcl-xL, while inhibiting the pro-apoptotic effects of Bax, effectively protecting neurons from ischemia, hypoxia, and other injuries (Hernández et al., 2017).

Second, anti-apoptotic drugs enhance cellular resistance to stress by activating endogenous protective pathways, including the PI3K/Akt, Nrf2, and heat shock protein pathways (Li et al., 2024a). These pathways help regulate autophagy levels, remove damaged organelles and proteins, and inhibit apoptosis, particularly in response to ER stress. For instance, miR-29a promotes axonal regeneration and inhibits ferroptosis by targeting the PTEN/PI3K/Akt pathway, with its exosome-based delivery system representing a promising therapeutic strategy (Zhao et al., 2021b). FG4592 activates the unfolded protein response (UPR) and autophagy following ischemic stroke by inhibiting OGFOD1 (Xie et al., 2024). This adaptive branching of the UPR, along with moderate autophagy, can clear misfolded proteins and alleviate ER stress, thereby inhibiting ER stress-induced apoptosis (e.g., via the C/EBP homologous protein pathway). The resulting neuroprotective effect contributes to improvements in neurological deficits, including cognitive impairments, after a stroke. Simvastatin has been found to reduce oligodendrocyte injury and impaired myelination, an essential process for cognitive development, in a neonatal hypoxic-ischemic model (Li et al., 2010). Its primary neuroprotective mechanisms include the activation of pro-survival pathways such as the PI3K/Akt pathway, inhibition of apoptosis, and protection of myelinating cells (both oligodendrocyte precursor cells and mature oligodendrocytes). Enhancing cellular stress resistance and autophagic capacity is a crucial strategy for combating apoptosis. Moderate activation of these pathways (e.g., PI3K/Akt, UPR, and autophagy) can maintain cellular homeostasis and protect neurons and glial cells, especially white matter/myelin, which is critical for cognitive function.

PTEN inhibitors enhance autophagic activity and reduce secondary hippocampal injury by inhibiting PTEN (Zhao et al., 2021a). Direct antioxidative effects help reduce oxidative stress-induced apoptosis by scavenging excessive ROS and blocking direct ROS-induced damage to biological macromolecules (e.g., lipids, proteins, and DNA), which can activate downstream pro-apoptotic signaling pathways (e.g., the JNK/p38 MAPK and mitochondrial pathways). One of the primary mechanisms underlying the neuroprotective effects of cannabidiol (Khaksar et al., 2022) in ischemic stroke models is its powerful antioxidant and anti-apoptotic properties. This is evidenced by significantly reduced expression of oxidative stress markers, inhibited caspase-3 activation, and alleviated neuronal apoptosis, all accompanied by improved neurobehavioral scores.

Currently available neuroprotective agents that have been used in the clinic (Reisberg et al., 2003; Pantoni et al., 2005; Alvarez-Sabín and Román, 2011; Jia et al., 2016; Zhao et al., 2022) function primarily by improving microcirculation and inhibiting excitotoxicity, oxidative stress, and inflammation (**Additional Table 2**). Some neuroprotective drugs have shown therapeutic effects in experimental studies of IS and have not been studied for PSCI, but they may also have therapeutic value in PSCI. Most of these drugs currently remain limited to animal models, and successful clinical translation will require addressing challenges related to targeted delivery and long-term safety. Combination therapies, such as those that integrate antioxidant and anti-inflammatory approaches, may overcome the limited efficacy of single agents (Wang et al., 2015). In the future, it will be necessary to combine multiomic technology with clinical translational research to develop drugs that ensure safety and multitargeted synergy to improve the long-term prognosis of patients with PSCI.

**Additional Table 2:** Neuroprotective treatment improves cognitive impairment after ischemic stroke

Drug	Representative drug	Main mechanism of action	Reference
NMDA receptor antagonists	Memantine	Modulate glutamate toxicity: a noncompetitive NMDA receptor antagonist that reduces excitotoxicity Improve synaptic plasticity: enhance BDNF expression	Zhao et al., 2022
Calcium channel blockers	Nimodipine	Improve cerebral blood flow: L-type calcium channel blockers, which dilate cerebral blood vessels Neuroprotection: reduce calcium overload and protect the integrity of the BBB	Pantoni et al., 2005
Free radical scavengers	Edaravone	Antioxidant: scavenger free radicals and inhibit lipid peroxidation Anti-inflammatory: reduce TNF-α and IL-1β levels Anti-apoptosis: inhibit caspase-3 activity and reduce neuronal apoptosis	Reisberg et al., 2003
Neurotrophin activators	Citicoline	Repair cell membranes: promote phospholipid synthesis Neurotransmitter regulation: increase acetylcholine and dopamine levels Anti-apoptosis: inhibit mitochondrial caspase pathway	Jia et al., 2016
Mitochondrial protective agents	Butylphthalide	Improve microcirculation: promote angiogenesis and collateral circulation Anti-inflammation: inhibit the NF-kB pathway Anti-oxidation: activate the Nrf2 pathway	Alvarez-Sabin and Roman, 2011

BBB: Blood-brain barrier; BDNF: brain-derived neurotrophic factor; IL-1β: interleukin-lbeta; NF-kB: nuclear factor kappa-light-chain-enhancer of activated B cells; NMDA: N-methyl-D- aspartic acid; Nrf2: nuclear factor erythroid 2-related factor 2; TNF-α: tumor necrosis factor-α.

### Chinese herbs and their extracts

Recent research has revealed that traditional Chinese medicines, along with their extracts or compounds, possess anti-inflammatory, antioxidative, and anti-apoptotic properties that can prevent cognitive impairment following cerebral ischemia. Ginsenoside Rg1, an extract of ginsenoside, effectively suppresses the inflammatory activation of brain microglia (Guan et al., 2023). This occurs through the inhibition of the TLR4/MyD88/NF-κB and TLR4/TRIF/IRF-3 pathways within microglia, thereby markedly enhancing the cognitive function of rats after MCAO. *Buyang Huanwu* Decoction is a traditional Chinese herbal prescription that has been used for a long time to treat cerebral ischemia and related diseases, including cognitive impairment (Xue et al., 2022). Treatment with *Buyang Huanwu* Decoction has been found to activate the PKA/CREB pathway, thereby reversing mitochondrial dysfunction, inhibiting hippocampal neuron apoptosis, and improving cognitive impairment in ischemic rats. Astragaloside IV safeguards mitochondrial hexokinase II and sustains glycolytic activity by activating Akt and facilitating its binding to hexokinase II. This action reduces the release of pro-apoptotic proteins and apoptosis-inducing factors, thus inhibiting the apoptosis of hippocampal neurons and ultimately alleviating cognitive dysfunction after ischemia (Li et al., 2019; Xue et al., 2019; Sun et al., 2020a). Other Chinese herbs and their active ingredients, such as *Salvia miltiorrhiza* (Wang et al., 2016), *Ginkgo biloba* (Li et al., 2017), *Rhizoma coptidi*s (Huang et al., 2017), and *Panax notoginseng* (Zhu et al., 2021), also have similar effects on improving cognitive impairment after ischemia. Although some traditional Chinese herbs, such as *Chuanxiong Rhizoma* and *Tatarinogradus tatarinogradus*, show potential effects, there is a lack of high-quality studies directly targeting cognitive impairment after cerebral ischemia.

Certain traditional Chinese medicines and their active ingredients may improve memory problems after ICH by affecting multiple targets. For example, Zhang et al. (2024c) reported that tetrahydrofolate can activate the PTEN signaling pathway and promote the growth of new nerve cells in the hippocampus, thereby promoting cognitive recovery after ICH. The PTEN pathway regulates cell death primarily by affecting the PI3K/Akt/mTOR pathway but can also enhance the strength and flexibility of connections between nerve cells. This suggests that folate metabolism is important for neural repair and may also be involved in mechanisms of epigenetic regulation, such as DNA methylation. Synergism among multiple components of traditional Chinese herbal medicines may provide advantages over single-component therapies. In the future, new technologies such as metabolomics can be used to identify the best combinations of traditional Chinese medicine formulations. As another example, a water-soluble component extracted from Danshen (Radix Salviae miltiorrhizae) was found to inhibit a specific mode of cell death known as ferroptosis after ICH. Specifically, this component can activate the Akt/GSK-3β/Nrf2 signaling pathway, thereby reducing the damage caused by ICH (Shi et al., 2024).

### Stem cell therapy

Stem cell therapy has emerged as a novel method for treating stroke, potentially achieving neuroprotection and repair through the secretion of various neurotrophic factors and the replacement of damaged neurons (Anthony et al., 2022). Wang et al. (2014) have demonstrated that stem cells secrete therapeutic factors that mitigate neuroinflammation, oxidative stress, and apoptosis, while promoting neurogenesis, angiogenesis, and mitochondrial repair. Bone marrow mononuclear cells include mesenchymal stem cells (MSCs), hematopoietic progenitor cells, and endothelial progenitor cells (Wang et al., 2014). The transplantation of bone marrow mononuclear cells has been shown to increase vascular density, reduce neuronal damage in the hippocampal CA1 subregion, lessen white matter damage, and improve learning and memory in rats following BCAO. The underlying mechanism likely involves the upregulation of the VEGF-VEGFR2 signaling pathway, which enhances angiogenesis.

MSCs are multipotent cells that exhibit anti-inflammatory, anti-apoptotic, and immunomodulatory capabilities (Qu et al., 2022). The transplantation of MSCs can mitigate neurological impairment induced by stroke through immune regulation. Moreover, MSCs can facilitate neurovascular remodeling and the recovery of neurological function following a stroke, potentially due to their ability to maintain BBB stability. Recent research has indicated that transplanted MSCs integrate with pericytes, astrocytes, and neurons, thereby promoting the integrity of the BBB. Esneault et al. (2008) reported that combined treatment with MSCs and erythropoietin enhanced endogenous cell proliferation and neurogenesis, leading to improved memory performance during striatal neurogenesis after transient focal cerebral ischemia. This improvement could explain the observed enhancement in long-term memory following cerebral ischemia.

Stem cell therapies mitigate cognitive impairment following cerebral ischemia through the synergistic effects of multiple mechanisms (Arakawa et al., 2023; Ikeda et al., 2024; Liu and Jia, 2024), including regeneration, anti-inflammatory effects, and vascular repair. However, it is imperative to address challenges associated with transplantation efficiency, safety, and mechanistic analysis (**Additional Table 3**). Future strategies may include the use of engineered stem cells, such as those genetically modified to enhance secretory function, as well as cell-free therapies utilizing exosomes. Although stem cells (such as NSCs and MSCs) have demonstrated reparative potential in IS, there have been no studies directly targeting cognitive impairment following hemorrhagic stroke.

**Additional Table 3:** Stem cell therapy improves cognitive impairment after ischemic stroke

Category	Source	Main mechanism of action	Reference
Stem cell secretions	Culture supernatant of MSCs or NSCs	(1) Carry miRNAs and proteins: such as miR-17-92 cluster, TSG101, promote synapse growth and anti-apoptosis. (2) Reduce oxidative stress: deliver antioxidant enzymes such as SOD and CAT.	Ikeda et al., 2024
Stem cells	MSCs (bone marrow, fat, umbilical cord)	(1) Paracrine effects: Neurotrophic factors, such as BDNF, VEGF, and IGF-1, are secreted to promote nerve regeneration and synaptic plasticity. (2) Anti-inflammatory: Inhibit the activation of microglia and reduce pro-inflammatory factors TNF-α and IL-6. (3) Promote angiogenesis: Exosomal delivery of miRNAs (such as miR-21-5p) activates the PI3K/Akt pathway.	Liu and Jia, 2024
Stem cells	NSCs (embryonic stem cells, induced differentiation)	(1) Differentiation into functional neurons: Involve integration into the host neural network and the reestablishment of synaptic connections. (2) Secretion of support factors: Release NT-3 and GDNF to support surviving neurons. (3) Regulation of microenvironment: Inhibit glial scar formation and promote endogenous repair.	Liu and Jia, 2024
Stem cells	iPSCs (somatic cell reprogramming such as fibroblasts)	(1) Directed differentiation into neuronal precursors: Replace dead neurons. (2) Regulation of synaptic plasticity: Enhance synaptic function through the expression of synaptic proteins (such as PSD-95 and synapsin-1).	Arakawa et al., 2023

Akt: Protein kinase B; BDNF: brain-derived neurotrophic factor; CAT: catalase; GDNF: glial cell line-derived neurotrophic factor; IGF-1: insulin-like growth factor 1; IL-6: interleukin-6; iPSCs: induced pluripotent stem cells; miR-17-92: microRNA cluster 17-92; miR-21-5p: MicroRNA-21-5p; MSCs: mesenchymal stem cells; NSCs: neural stem cells; NT-3: neurotrophin-3; PI3K: phosphoinositide 3-kinase; PSD-95: postsynaptic density protein 95; SOD: superoxide dismutase; TNF-α: tumor necrosis factor alpha; TSG101: tumor susceptibility gene 101; VEGF: vascular endothelial growth factor.

### Other treatments

Emerging evidence supports non-pharmacological interventions, such as acupuncture, for PSCI. As an ancient traditional Chinese medicine modality, acupuncture has demonstrated therapeutic efficacy through targeted acupoint stimulation, promoting neurogenesis, axonal regeneration, and synaptic remodeling while suppressing apoptosis, inflammation, and oxidative stress (Qin et al., 2022; Li et al., 2023c).

Neurorehabilitative strategies are clinically employed to address PSCI. Repetitive transcranial magnetic stimulation (TMS) (Sheng et al., 2023; Zhou et al., 2023) is a neuroelectrophysiological technique that modulates neuronal excitability through sustained postsynaptic potential summation, enhancing the post-stroke recovery of executive function, memory, language, and visuospatial processing. Complementary approaches, including exercise therapy (Xing and Bai, 2020), occupational therapy (Lanctôt et al., 2020), and hyperbaric oxygen therapy (Hadanny et al., 2020), further optimize cognitive outcomes (Lee et al., 2013; Kober et al., 2017; Laver et al., 2017; Aprile et al., 2020; Daniel et al., 2021; Ho et al., 2022; Xu et al., 2022a; Gangemi et al., 2023; **Additional Table 4**).

**Additional Table 4:** Other treatments for cognitive impairment after ischemic stroke

Treatment measure	Methods	Mechanism of action	Reference
Exercise therapy	Utilize aerobic, resistance, or multicomponent exercise regimens for active/passive intervention.	Enhance neurotrophic factor levels Improve dendritic/axonal morphology and synaptic plasticity; Promote neurogenesis; Increase cerebral blood flow and gray matter volume; Reduce vascular risk factors; Induce neural compensation in peri-infarct regions.	Xing and Bai, 2020
Occupational therapy	Engage multisensory integration (visual, auditory, tactile, vestibular, and proprioceptive inputs) to drive adaptive cortical reorganization.	Facilitate object recognition through repetitive sensory feedback; Enhance synaptic remodeling via enriched environmental stimulation; Promote neuroplasticity through task-specific neural circuit reinforcement.	Lanctôt et al., 2020
Transcranial magnetic stimulation	Apply repetitive magnetic pulses to modulate cortical excitability via summation of excitatory postsynaptic potentials.	Regulate glutamatergic neurotransmission; Stimulate neurogenesis and angiogenesis; Exert antioxidant/anti-apoptotic effects; Suppress neuroinflammation; Augment cerebral perfusion.	Zhou et al., 2023
Hyperbaric oxygen therapy	Deliver 100% oxygen at >1 atmospheric pressure in sealed chambers to enhance tissue oxygenation.	Activate endogenous neural stem cell proliferation; Promote axonal regeneration and remyelination; Reduce blood-brain barrier permeability; Mitigate mitochondrial dysfunction via astrocyte-neuron transfer; Inhibit apoptosis to preserve peri-ischemic tissue.	Hadanny et al., 2020
Music therapy	Employ music-based interventions (listening, singing, instrumental play, and composition) to engage multimodal neural networks.	Activate bilateral fronto-temporo-parietal networks; Enhance attentional allocation and semantic processing; Strengthen motor-auditory coupling via rhythm entrainment; Facilitate memory consolidation through limbic system engagement.	Daniel et al., 2021; Xu et al., 2022a
Rehabilitation robotics	Combine flexible actuators, AI-driven human-machine interfaces, and IoT technologies to deliver personalized motor training (passive/active/resistive modes).	Improve executive function through multisensory-motor integration; Enhance neuroplasticity via task-oriented repetition; Provide real-time biofeedback for cortical remapping; Adapt training intensity using machine learning algorithms.	Aprile et al., 2020
Brain-computer interface	Establish direct neural-electronic communication pathways to decode cortical activity into device commands.	Enhance motor-cognitive plasticity through neurofeedback; Promote neural circuit reorganization via closed-loop stimulation; Facilitate compensatory network formation in damaged regions.	Lee et al., 2013; Kober et al., 2017
Computer-assisted cognitive training	Implement multimodal sensory stimulation (audiovisual tasks and gamified modules) for adaptive neurocognitive rehabilitation.	Induce synaptic plasticity through Hebbian learning mechanisms; Establish alternative cognitive neural circuits; Promote functional reorganization via distributed network engagement.	Ho et al., 2022
Virtual reality therapy	Simulate immersive environments via head-mounted displays/projection systems with multisensory feedback (visual, auditory, haptic, and olfactory).	Enhance spatial navigation through hippocampal engagement; Improve motor learning via visuomotor synchronization; Strengthen attentional networks through ecological validity; Facilitate fear extinction via controlled exposure therapy.	Laver et al., 2017; Gangemi et al., 2023

## Safety

Patients with PSCI typically aim to achieve the goals of slowing cognitive decline, enhancing cognitive function, and improving daily living capabilities during treatment. The therapeutic approaches generally include pharmacological interventions, management of neuropsychiatric symptoms, and rehabilitation therapies (**Additional Table 5**).

**Additional Table 5:** The adverse reactions, toxic effects and safety of treatment techniques

Treatment classification	Drug or method category	Adverse reactions	Toxic reactions	Safety assessment	Reference
Drug treatment	Cholinesterase inhibitors (such as Donepezil, Rivastigmine, and Galantamine)	Gastrointestinal reactions (nausea, vomiting, diarrhea, and incidence rate 20%-30%); Cardiovascular reactions (bradycardia, syncope, especially when combined with beta-blockers); central nervous system (headache, insomnia, and nightmares).	Cholinergic crisis (manifested by tremors, convulsions and respiratory depression when excessive); hepatotoxicity (elevated ALT, rare but needs monitoring).	Before medication administration, it is necessary to screen for electrocardiogram (risk of Q-T interval prolongation); for older adult patients, start with a low dose (such as donepezil 5 mg/d); conduct long-term follow-up on cognitive function (to avoid attenuation of therapeutic effect).	Zhang and Gordon, 2018
	NMDA receptor antagonist (such as Memantine)	Dizziness (10%-15%), drowsiness; hallucinations (especially in patients with Lewy body dementia).	High doses (> 20 mg/d) may aggravate mental symptoms or movement disorders; patients with renal insufficiency may accumulate toxicity (and dosage adjustment is necessary).	Monitoring of creatinine clearance rate (halve the dose when eGFR is less than 30 mL/min); avoid combination with amantadine (additive dopaminergic toxicity).	Kishi et al., 2017
	Neurotrophic drugs (such as Ganglioside GMland rat nerve growth factor)	Injection site pain and induration; allergic reactions (such as rash and fever).	Autoimmune neuritis (mediated by anti-GMl antibody); NGF-induced hyperalgesia (abnormal activation of dorsal root ganglion).	Before taking the medicine, a skin test is required (especially for GM1); The treatment course is limited (usually ≤ 4 wk to avoid long-term dependence).	Jeon et al., 2016
Non-drug treatment	Neural Transcranial regulation magnetic technology stimulation	Stabbing pain on the scalp and temporary sensitivity of hearing (ear protection is required).	Epileptic seizure (high-risk population: history of epilepsy in the past, and abnormal brain structure).	Screening for epilepsy risk (EEG pre-screening); parameter optimization (frequency ≤ 10 Hz, intensity ≤ 120% of maximal threshold).	Chou et al., 2022
	Transcranial direct current stimulation	Skin itching and mild headache.	Electrode-induced skin burns (when the current density exceeds 0.1 mA/cm^2^).	Avoid continuous stimulation for more than 20 min; Exclude patients with implanted metallic devices.	Begemann et al., 2020
	Stem cell therapy (such as mesenchymal stem cells and neural progenitor cells)	Acute infusion reactions (such as fever and chills); immunological rejection (cross-matching required for allogeneic sources).	Risk of teratoma (undifferentiated pluripotent stem cells); abnormal migration (cells colonize in non-target areas).	Imaging monitoring (MRI tracking of cell distribution); long-term follow-up (at least 5 yr, monitoring of tumor markers).	Wang et al., 2013
	Deep brain stimulation (applied for severe cases, the target areas: the hippocampus and the fornix)	Surgery-related (infection, bleeding, incidence rate 1%-3%); electrode displacement or fracture	Memory interference (resulting from improper stimulus parameters leading to retrograde amnesia); status epilepticus (rare).	Preoperative fMRI localization (avoiding areas with dense blood vessels); postoperative program-controlled optimization (voltage ≤ 3 V, frequency ≤ 130 Hz).	Heminghyt et al., 2022
Rehabilitation training and combined therapy	Cognitive rehabilitation training	Training fatigue (especially for severe cases); anxiety or frustration (due to improper difficulty setting).	Excessive training leads to metabolic overload of the hippocampus (as shown by fMRI, lactic acid accumulation).	Individualized plan (Montreal Cognitive Assessment score stratification); combined with heart rate variability monitoring for stress level assessment.	Huang et al., 2022b
	Drug-rehabilitation combined therapy (common combinations: Donepezil + rTMS)	Cholinergic enhancement superimposed effect (increased severity of nausea and dizziness); headache after stimulation (incidence increased by 15%).		Interval administration and stimulation time (such as taking medication 2 h after rTMS); Monitoring cholinesterase activity (avoiding excessive inhibition).	Bell et al., 2021
New therapeutic technology (in the research stage)	Gene therapy (such as AAV vector-mediated delivery of neurotrophic factors)	Not described.	Carrier-related inflammation (AAV antibody neutralization effect); gene off-target (such as BDNF overexpression inducing epilepsy).	Preclinical primate model validation; Monitoring of cerebrospinal fluid antibody titers.	Sevigny et al., 2024
	Nanomedicine delivery system	Not described.	Nanoparticle hepatic/kidney accumulation (long-term toxicity of gold nanoparticles); disruption of blood-brain barrier (side effect of ultrasound microbubbles assistance).	Biodegradable materials are given priority (such as PLGA); dynamic enhancement of MRI monitoring of barrier integrity.	Yi et al., 2020

AAV: Adeno-associated virus; ALT: alanine aminotransferase; BDNF: brain-derived neurotrophic factor; EEG: electroencephalogram; eGFR: estimated glomerular filtration rate; fMRI: functional magnetic resonance imaging; GM1: ganglioside GM1; MRI: magnetic resonance imaging; NGF: nerve growth factor; NMDA: N-methyl-D-aspartate; PLGA: poly (lactic-co-glycolic acid); rTMS: repetitive transcranial magnetic stimulation.

In cases of PSCI, according to the 2021 Expert Consensus on the Management of PSCI, medications such as cholinesterase inhibitors and non-competitive NMDA receptor antagonists may be used (Ravi et al., 2025). For instance, donepezil has been shown to improve performance in trail-making tests and Mini-Mental State Examination scores. Galantamine can enhance behavioral ratings but may induce gastrointestinal adverse effects. Memantine has demonstrated efficacy in alleviating aphasia following stroke. Additionally, deproteinized calf blood extract may contribute to cognitive improvement (Wang et al., 2013; Jeon et al., 2016; Kishi et al., 2017; Zhang and Gordon, 2018; Begemann et al., 2020; Yi et al., 2020; Bell et al., 2021; Chou et al., 2022; Heminghyt et al., 2022; Huang et al., 2022b; Sevigny et al., 2024).

Patients with PSCI often have symptoms of depression, anxiety, and delusions. If symptoms are mild, they can be managed with non-pharmacological approaches, such as clinical care and physical exercise. If the symptoms are severe, then medication is needed: selective serotonin reuptake inhibitors (which are antidepressants) can reduce depressive symptoms (Ravi et al., 2025); *Ginkgo biloba* extract can treat psychiatric symptoms; Lower doses of newer antipsychotic medications are appropriate for treating delusions and related symptoms. Cognitive training and psychological interventions are an important part of rehabilitation and include training attention, perception, long-term memory, working memory, numeration, and executive function. In addition, aerobic exercise and cognitive training may be better done together (Yu et al., 2021). Complementary treatments such as acupuncture, TMS, and transcranial direct current stimulation (tDCS) can also be considered (Li et al., 2023c). Some adverse reactions are inevitable during treatment, such as taking too much medicine or accumulating too much medicine in the body, which may cause toxic reactions; This reaction, although serious, can be thought of and prevented in advance. Therefore, it is very important for patients and their families to learn to identify and deal with these problems. To ensure the safety of patients, it is necessary to check and pay attention to possible adverse reactions when taking medicine, so that problems can be found and treated in time.

## Role of Hippocampal Cell Dysfunction in Other Forms of Cognitive Decline and Its Treatment

The aetiology of cognitive impairment is multifactorial. In addition to the aforementioned PSCI, there are many other diseases related to cognitive impairment. Age-related cognitive decline (ARCD) refers to a non-pathological, gradual, and widespread mild deterioration in cognitive functions (including processing speed, working memory, executive function, and episodic memory) that occurs during the normal aging process. The underlying mechanisms of ARCD include slowly progressing neurobiological aging, vascular aging, epigenetic changes, and a diffuse decline in brain functional network connectivity. The main manifestations are diffuse microscopic changes, such as synaptic loss, white matter lesions, and microangiopathy (Gonzales et al., 2022; Wang et al., 2024). The pathological mechanism of PSCI is related to acute focal brain injury and its triggering of severe secondary injury cascades, including inflammation, excitotoxicity, BBB disruption, and apoptosis, as well as white matter lesions and neuroplasticity changes. The anatomical basis of PSCI is the presence of identifiable macroscopic lesions resulting from brain injury, such as infarction or hemorrhage. Although both patients with ARCD and PSCI eventually experience cognitive decline, their starting points, driving factors, underlying mechanisms, pathophysiological processes, and lesion characteristics are considerably different. However, ARCD does not meet the diagnostic criteria for dementia, is considered part of “successful aging,” and lacks clear macroscopic lesions (Hullinger and Puglielli, 2017). Therefore, this review focuses on non-ARCD-related cognitive disorders.

A variety of neurodegenerative diseases, such as AD, dementia with Lewy bodies, and Parkinson’s disease dementia, are also core drivers of cognitive decline. The aetiology of AD is complex, and many studies have focused on the amyloid hypothesis, the microtubule-associated protein hypothesis, and genetics (Yao et al., 2024). The hippocampus is one of the earliest regions affected by the pathology. Neurofibrillary tangles caused by Aβ plaque deposition and abnormal tau protein phosphorylation directly impair synaptic plasticity and inhibit neurogenesis, resulting in progressive hippocampal atrophy, which is significantly associated with clinical memory deficits (Geigenmüller et al., 2024).

AD shows familial aggregation, with approximately 20% of patients having a positive family history, suggesting that genetic factors play a crucial role in its pathogenesis. Apolipoprotein E (APOE) has long been regarded as the most significant genetic factor regulating the risk of sporadic AD. APOE influences the average age of onset and lifetime probability of developing AD. The APOEε4 allele significantly increases the risk of AD (Jackson et al., 2024). The APOEε4 allele is crucial for the downstream impacts of Aβon the aggregation of phosphorylated tau in the living human brain (Ferrari-Souza et al., 2023). APOE4 can affect neuronal synaptic activity and function, lipid metabolism related to astrocytes, and immunoreactivity in microglial models derived from induced pluripotent stem cells. Compared to syngeneic APOE3, APOE4 variants are characterized by a greater number of synapses and enhanced secretion of Aβ42 in neurons. Moreover, APOE4 astrocytes exhibit defective Aβ uptake and cholesterol accumulation (Lin et al., 2018). AD that occurs before the age of 65 is usually called early-onset AD. Mutations in the APP gene and the PSEN1 and PSEN2 genes located on chromosome 21 are the three major causes of familial early-onset AD. Mutant APP produces excess Aβ42 through amylogenesis, forming soluble oligomers that directly impair synaptic function (Boon et al., 2020; Drummond et al., 2022). The PSEN1 and PSEN2 genes encode key components of γ-secretase, and dysfunction (caused by pathogenic mutations) leads to excessive production of toxic Aβ42, which is the core link in triggering the pathological cascade of early-onset familial AD (Li et al., 2022; Xia et al., 2022a). In addition, BDNF, PICALM, and CLU are widely recognized as important risk genes in AD research (Caffino et al., 2020; Maturana-Candelas et al., 2021; Ando et al., 2022; Gao et al., 2022).

In Lewy body dementia and Parkinson’s disease dementia, the abnormal aggregation of α-synuclein primarily disrupts the functional connectivity between the hippocampus and cortex, inducing visual‒spatial memory impairments. Although hippocampal atrophy is less pronounced in Lewy body dementia and Parkinson’s disease dementia than in AD, impaired neurogenesis remains a prominent feature (Xu et al., 2020; Villar-Conde et al., 2021).

Therapeutic strategies for treating hippocampal dysfunction in degenerative diseases have advanced in many ways. Drug development focuses on enhancing neurogenesis and clearing pathological proteins. For example, the BDNF enhancer LM22A-4 improves hippocampus-dependent memory in AD animal models by activating the TrkB receptor, while the anti-Aβ monoclonal antibody Lecanemab has demonstrated potential in slowing hippocampal atrophy during phase III clinical trials (van Dyck et al., 2023). Nonpharmacological interventions have also shown significant efficacy: meta-analyses have confirmed that regular aerobic exercise increases hippocampal volume in patients with mild cognitive impairment and that high-frequency TMS enhances memory function in patients with AD by modulating the connectivity between the hippocampus and the default mode network (Tuo et al., 2021; Yu et al., 2021). Nevertheless, current research must address the challenges of precise targeted therapies, such as elucidating the specific damage patterns of hippocampal subregions (e.g., CA1 and DG) across different diseases and exploring the diagnostic utility of biomarkers (e.g., tau PET imaging). Future combination therapies that integrate neurogenic promoters with anti-inflammatory agents may offer promising avenues for reversing cognitive decline.

What are the differences in the pathological mechanisms of cognitive impairment between PSCI and other degenerative diseases?

PSCI begins with a definitive cerebrovascular event (e.g., ischemic or hemorrhagic stroke) that causes not only acute focal brain tissue damage but also progressive neurodegeneration that extends well beyond the infarct focus through multiple cascades. Stroke directly leads to severe disruption of the BBB, microcirculatory disorders, and acute hypoxia-ischemic injury, releasing damage-related molecular patterns and triggering an intense early neuroinflammatory response (Sun et al., 2014). This clear angiogenic origin is fundamentally different from the primary degenerative mechanisms found in diseases such as AD, which is characterized by the abnormal accumulation of Aβ and tau proteins, and dementia with Lewy bodies, which involves α-synuclein aggregation. In PSCI, there is often direct hippocampal infarction, such as acute infarction in the CA1 region. In contrast, in other neurodegenerative diseases, hippocampal involvement is relatively mild and occurs later, with less obvious neuronal loss. Persistent BBB dysfunction, chronic hypoperfusion, and an inflammatory microenvironment collectively drive secondary protein pathology following a stroke. Increased BBB permeability impairs the clearance of Aβ (e.g., by LRP1 transporters), while ischemia and hypoxia enhance APP cleavage and promote Aβ deposition. However, the distribution of Aβ deposition differs from the extensive neocortical plaques seen in AD, occurring as a post-stroke event rather than as an initial cause (Ouyang et al., 2021; Kang et al., 2023b). Additionally, excitotoxicity and oxidative stress activate kinases, leading to tau hyperphosphorylation (Chi et al., 2019; Huang et al., 2022a). However, the propagation of tau in PSCI often follows stroke-related neural networks, which is distinct from the typical preferential involvement of the entorhinal cortex and hippocampus observed in AD.

## Limitations

This review has the following limitations. First, due to space constraints and the specific focus of the topic, the mechanisms and targeted therapies for hippocampal cell dysfunction in cognitive decline diseases other than AD (such as vascular dementia and frontotemporal dementia) have not been systematically analyzed. Second, several emerging fields have not been comprehensively explored, including the pattern of cognitive reorganization in adolescent depression, the interaction mechanisms between metabolic disorders (especially diabetes) and cognitive impairment, and electroencephalogram-based prediction models for diabetes-related cognitive decline. These advancements hold significant potential for interdisciplinary cognitive intervention and merit special review. Third, impaired neurogenesis in the DG region of the hippocampus is observed not only in patients with AD but also in those with bipolar disorder and schizophrenia. These neurobiological commonalities across diagnoses provide new insights for developing broad-spectrum cognitive enhancement therapies, which cannot be explored in depth in this paper due to space limitations. Finally, other key factors contributing to cognitive decline, beyond hippocampal atrophy and their complex interactions, were not adequately included in our study. These factors include age-related natural changes, cerebrovascular health status (influenced by hypertension, diabetes, high cholesterol, and smoking), lifestyle choices (e.g., exercise, cognitive activity, and a healthy diet), and genetic predisposition. A comprehensive understanding of cognitive decline requires the integration of these multiple factors, which represents an important limitation of this review.

## Conclusions and Future Perspectives

Great progress has been made in understanding PSCI through preclinical animal models that recapitulate disease pathology. MCAO and BCAO/BCAS are the predominant rodent models for studying IS and associated cognitive deficits (Fluri et al., 2015). These models enable mechanistic exploration of hippocampal neuron apoptosis, synaptic plasticity impairment, and neurovascular unit dysfunction. However, several key challenges in model standardization exist. One such challenge is age-dependent recovery bias: young and middle-aged animals exhibit better neurological recovery than their aged counterparts, introducing translational limitations given the clinical prevalence of stroke in elderly patients. Additionally, neglecting comorbidities such as hypertension, diabetes, and smoking, which are common clinical risk factors, remains a concern. It is essential to incorporate these factors into multifactorial models to improve clinical relevance.

Through establishment of these models, the cellular mechanisms that may be involved in cognitive impairment after cerebral ischemia have been studied, revealing that cognitive impairment is related to neurons in the hippocampus. Both endogenous and exogenous neuronal apoptosis pathways may be involved in the mechanisms of cognitive impairment following cerebral ischemia. Various signaling pathways, such as the NRF2-ARE-NF-κB and PI3K/Akt pathways, play roles in neuronal apoptosis. Neuronal synaptic plasticity, which is essential for the integration and transmission of information in the nervous system and the basis of memory formation and maintenance, significantly impacts cognitive impairment after cerebral ischemia. Increasing evidence suggests that nanoparticles (Chen et al., 2022) can cross the BBB in different ways and act directly or indirectly on nerve cells to modulate synaptic plasticity and ultimately improve neural function. These findings provide guidance for future research on cognitive impairment after cerebral ischemia and serve as an important reference for subsequent studies.

As mentioned above, hippocampal neurons can regenerate, and NSCs may be activated in the hippocampus after cerebral ischemia to repair damaged hippocampal tissue through proliferation and differentiation. This process may help reduce the degree of cognitive impairment following cerebral ischemia. Hippocampal neuron regeneration involves not only the generation of new neurons but also the reorganization and remodeling of connections between existing neurons (Yang et al., 2015; Li et al., 2018). This neuroplasticity is critical for restoring impaired cognitive function. Promoting the regeneration and neuroplasticity of hippocampal neurons may help improve cognitive impairment following cerebral ischemia. There is a strong connection between hippocampal neuron regeneration and cognitive impairment after cerebral ischemia. Although the regenerative capacity of hippocampal neurons in adulthood is limited, it is still possible to reduce the extent of cognitive impairment after cerebral ischemia by promoting hippocampal neuron regeneration and neuroplasticity.

The interaction and mutual influence of astrocytes, microglia, and OLs after cerebral ischemia are related to the cognitive impairment induced by IS. Various descriptive experimental studies have been conducted to elucidate these cellular mechanisms and reveal the relevant pathways leading to cognitive impairment after cerebral ischemia. These mechanisms need to be investigated in depth to identify favorable targets for future studies of ischemia-induced cognitive impairment (Jadhav et al., 2023; Rangel-Gomez et al., 2024).

The pathological changes following cerebral ischemia are complex, and nerves and blood vessels are closely interconnected. Therefore, a single type of neuroprotection can also protect entire nerves and blood vessels; this observation has led to the concept of the “neurovascular unit (NVU)”. The NVU consists of astrocytes, mural vascular smooth muscle cells, pericytes, and endothelial cells, and plays a crucial role in regulating neurovascular coupling (Kisler et al., 2017). After cerebral ischemia, neuronal ischemia and hypoxia cause energy metabolism disorders, leading to neuronal dysfunction and death. Neuronal damage is a key pathological basis of cognitive impairment following cerebral ischemia. Astrocytes and microglia undergo a series of reactive processes, including proliferation, migration, and activation, which contribute to neuronal protection and repair. However, excessive responses may also exacerbate neuronal damage and death. Cerebral blood vessels are closely connected to nerves, and microvessels and neurons are aligned through growth along the paths of the extracellular matrix. Astrocytes and endothelial cells work together to establish an intermediate basal barrier and interendothelial tight junctions, which are components of the capillary permeability barrier (del Zoppo, 2010). After ischemia, activated astrocytes and pericytes release vasoactive mediators. The balance of vasoconstriction and vasodilation controls the tone of the peripheral vasculature and regulates regional cerebral blood flow (Zhao et al., 2020). The core function of the NVU is to form and maintain the BBB. Repairing the BBB (e.g., enhancing endothelial tight junctions, restoring pericyte function, and regulating astrocyte end-feet) can reduce harmful substance infiltration, neuroinflammation, and neurotoxicity, thereby protecting neurons and synapses and maintaining cognitive function. Thus, the NVU plays a considerable role in cognitive impairment after cerebral ischemia. By regulating the interactions and signal transduction between neurons, glial cells, and cerebral vessels, the NVU can affect neuronal survival and repair, as well as cognitive function recovery after cerebral ischemia. Xu et al. (2024) have indicated that noninvasive neuromodulation techniques, such as transcranial direct current stimulation (tDCS), can promote neurovascular coupling repair by targeting the interaction between NVU components, making it a promising interventional strategy. tDCS may indirectly promote functional remodeling of the NVU by increasing the cortical excitability of damaged brain areas, optimizing the local hemodynamic response, and regulating neurovascular signaling pathways. These findings not only validate the clinical effectiveness of tDCS but also support the therapeutic strategy of noninvasive neuromodulation technology to alleviate neurological deficits by repairing the functional network of the NVU and reversing microcirculation disorders at the mechanistic level. Consequently, therapeutic strategies targeting the NVU may offer new ideas and targets for the treatment of cognitive impairment after cerebral ischemia.

Interventional drugs for cognitive impairment following cerebral ischemia include traditional Chinese medicines and their extracts, neuroprotective drugs, and stem cell therapies. Many of these drugs have unique advantages in terms of inhibiting apoptosis, promoting neurogenesis, inhibiting neuroinflammation, suppressing the release of pro-inflammatory cytokines, and regulating the central cholinergic system, highlighting their great potential for the prevention and treatment of cognitive impairment after cerebral ischemia. However, most existing studies have been based on animal experiments, with relatively few clinical studies available. Future research should focus on clinical investigations of interventions for cognitive impairment after cerebral ischemia to provide new ideas for treatment.

Cognitive impairment following ICH arises from neuronal aplasia, synaptic loss, glial cell inflammation, and white matter damage. Future research should aim to elucidate multicellular interaction networks, such as the microglia-neuron-astrocyte dialogue, as well as cross-scale mechanisms, including the crosstalk between iron metabolism and the complement system. Treatment strategies should integrate multitarget interventions, such as iron chelation (e.g., deferoxamine), complement inhibition (e.g., B4Crry), stem cell therapies (e.g., human umbilical cord blood-derived MSCs), and modulation of autophagic/apoptotic pathways. Additionally, advanced imaging biomarkers, such as those determined by quantitative susceptibility mapping and DTI, should be used to accurately monitor dynamic changes in hippocampal function for clinical translation.

In conclusion, this review offers practical guidance for the accurate diagnosis and the effective, safe, and personalized treatment of hippocampal pathologies.

## Additional files:

***Additional Table 1:*** *Characteristics of commonly used animal models of cognitive impairment after ischemic stroke.*

***Additional Table 2:*** *Neuroprotective treatment improves cognitive impairment after ischemic stroke.*

***Additional Table 3:*** *Stem cell therapy improves cognitive impairment after ischemic stroke.*

***Additional Table 4:*** *Other treatments for cognitive impairment after ischemic stroke.*

***Additional Table 5:*** *The adverse reactions, toxic effects and safety of treatment techniques.*

## References

[R1] Adamsky A, Kol A, Kreisel T, Doron A, Ozeri-Engelhard N, Melcer T, Refaeli R, Horn H, Regev L, Groysman M, London M, Goshen I (2018). Astrocytic activation generates de novo neuronal potentiation and memory enhancement. Cell.

[R2] Aggleton JP, O’Mara SM (2022). The anterior thalamic nuclei: core components of a tripartite episodic memory system. Nat Rev Neurosci.

[R3] Ahnaou A, White E, Biermans R, Manyakov NV, Drinkenburg W (2020). In vivo plasticity at hippocampal schaffer collateral-CA1 synapses: replicability of the ltp response and pharmacology in the long-evans rat. Neural Plast.

[R4] Alam MJ, Kitamura T, Saitoh Y, Ohkawa N, Kondo T, Inokuchi K (2018). Adult neurogenesis conserves hippocampal memory capacity. J Neurosci.

[R5] Alawieh AM, Langley EF, Feng W, Spiotta AM, Tomlinson S (2020). Complement-dependent synaptic uptake and cognitive decline after stroke and reperfusion therapy. J Neurosci.

[R6] Alvarez-Sabín J, Román GC (2011). Citicoline in vascular cognitive impairment and vascular dementia after stroke. Stroke.

[R7] Ando K, Nagaraj S, Küçükali F, de Fisenne MA, Kosa AC, Doeraene E, Lopez Gutierrez L, Brion JP, Leroy K (2022). PICALM and Alzheimer’s disease: an update and perspectives. Cells.

[R8] Anthony S, Cabantan D, Monsour M, Borlongan CV (2022). Neuroinflammation, stem cells, and stroke. Stroke.

[R9] Aprile I, Guardati G, Cipollini V, Papadopoulou D, Mastrorosa A, Castelli L, Monteleone S, Redolfi A, Galeri S, Germanotta M (2020). Robotic rehabilitation: an opportunity to improve cognitive functions in subjects with stroke. An explorative study. Front Neurol.

[R10] Arakawa M, Sakamoto Y, Miyagawa Y, Nito C, Takahashi S, Nitahara-Kasahara Y, Suda S, Yamazaki Y, Sakai M, Kimura K, Okada T (2023). iPSC-derived mesenchymal stem cells attenuate cerebral ischemia-reperfusion injury by inhibiting inflammatory signaling and oxidative stress. Mol Ther Methods Clin Dev.

[R11] Archie SR, Al Shoyaib A, Cucullo L (2021). Blood-brain barrier dysfunction in CNS disorders and putative therapeutic targets: an overview. Pharmaceutics.

[R12] Artyukhina NI, Sarkisova KY (2007). A possible mechanism of unilateral hippocampal stroke after bilateral occlusion of the common carotid arteries in rats with different types of behavior. Neurosci Behav Physiol.

[R13] Bai T, Zhan L, Zhang N, Lin F, Saur D, Xu C (2023). Learning-prolonged maintenance of stimulus information in CA1 and subiculum during trace fear conditioning. Cell Rep.

[R14] Barros CA, Ekuni R, Moro MA, Pereira FM, Dos Santos Pereira MA, Milani H (2009). The cognitive and histopathological effects of chronic 4-vessel occlusion in rats depend on the set of vessels occluded and the age of the animals. Behav Brain Res.

[R15] Begemann MJ, Brand BA, Ćurčić-Blake B, Aleman A, Sommer IE (2020). Efficacy of non-invasive brain stimulation on cognitive functioning in brain disorders: a meta-analysis. Psychol Med.

[R16] Bell MD, Pittman B, Petrakis I, Yoon G (2021). Donepezil and cognitive remediation therapy to augment treatment of alcohol use disorder related mild cognitive impairment (AUD-MCI): An open label pilot study with historical controls. Subst Abus.

[R17] Berghout BP, Soyupak RF, Ikram MK, Bos D (2025). Variations in intracranial arterial anatomy of the circle of Willis and their association with arteriosclerosis in patients with ischemic cerebrovascular disease. Int J Stroke.

[R18] Bin Ibrahim MZ, Benoy A, Sajikumar S (2022). Long-term plasticity in the hippocampus: maintaining within and ‘tagging’ between synapses. FEBS J.

[R19] Blanco-Suárez E (2023). Photothrombotic model to create an infarct in the hippocampus. Methods Mol Biol.

[R20] Boon BDC, Bulk M, Jonker AJ, Morrema THJ, van den Berg E, Popovic M, Walter J, Kumar S, van der Lee SJ, Holstege H, Zhu X, Van Nostrand WE, Natté R, van der Weerd L, Bouwman FH, van de Berg WDJ, Rozemuller AJM, Hoozemans JJM (2020). The coarse-grained plaque: a divergent Aβ plaque-type in early-onset Alzheimer’s disease. Acta Neuropathol.

[R21] Borczyk M, Radwanska K, Giese KP (2021). The importance of ultrastructural analysis of memory. Brain Res Bull.

[R22] Borzello M, Ramirez S, Treves A, Lee I, Scharfman H, Stark C, Knierim JJ, Rangel LM (2023). Assessments of dentate gyrus function: discoveries and debates. Nat Rev Neurosci.

[R23] Bowden JB, Abraham WC, Harris KM (2012). Differential effects of strain, circadian cycle, and stimulation pattern on LTP and concurrent LTD in the dentate gyrus of freely moving rats. Hippocampus.

[R24] Buffington SA, Huang W, Costa-Mattioli M (2014). Translational control in synaptic plasticity and cognitive dysfunction. Annu Rev Neurosci.

[R25] Cabeza R, Ciaramelli E, Moscovitch M (2012). Cognitive contributions of the ventral parietal cortex: an integrative theoretical account. Trends Cogn Sci.

[R26] Caffino L, Mottarlini F, Fumagalli F (2020). Born to protect: leveraging BDNF against cognitive deficit in Alzheimer’s disease. CNS Drugs.

[R27] Cao D, Li B, Cao C, Zhang J, Li X, Li H, Yu Z, Shen H, Ye M (2023). Caveolin-1 aggravates neurological deficits by activating neuroinflammation following experimental intracerebral hemorrhage in rats. Exp Neurol.

[R28] Cao Q, Chen J, Zhang Z, Shu S, Qian Y, Yang L, Xu L, Zhang Y, Bao X, Xia S, Yang H, Xu Y, Qiu S (2023). Astrocytic CXCL5 hinders microglial phagocytosis of myelin debris and aggravates white matter injury in chronic cerebral ischemia. J Neuroinflammation.

[R29] Cechetti F, Worm PV, Lovatel G, Moysés F, Siqueira IR, Netto CA (2012). Environmental enrichment prevents behavioral deficits and oxidative stress caused by chronic cerebral hypoperfusion in the rat. Life Sci.

[R30] Chakraborty S, Tripathi SJ, Srikumar BN, Raju TR, Shankaranarayana Rao BS (2020). N-acetyl cysteine ameliorates depression-induced cognitive deficits by restoring the volumes of hippocampal subfields and associated neurochemical changes. Neurochem Int.

[R31] Chambers LC, Diaz-Otero JM, Fisher CL, Jackson WF, Dorrance AM (2022). Mineralocorticoid receptor antagonism improves transient receptor potential vanilloid 4-dependent dilation of cerebral parenchymal arterioles and cognition in a genetic model of hypertension. J Hypertens.

[R32] Chau JPC, Lo SHS, Zhao J, Choi KC, Butt L, Lau AYL, Mok VCT, Kwok ZCM, Thompson DR (2023). Prevalence of post-stroke cognitive impairment and associated risk factors in Chinese stroke survivors. J Neurol Sci.

[R33] Chen A, Kang Y, Liu J, Wu J, Feng X, Wang M, Zhang Y, Wang R, Lai X, Shao L (2022). Improvement of synaptic plasticity by nanoparticles and the related mechanisms: applications and prospects. J Control Release.

[R34] Chen T, Cai C, Wang L, Li S, Chen L (2020). Farnesyl transferase inhibitor lonafarnib enhances α7nAChR expression through inhibiting DNA methylation of CHRNA7 and increases α7nAChR membrane trafficking. Front Pharmacol.

[R35] Chen Z, Gao C, Hua Y, Keep RF, Muraszko K, Xi G (2011). Role of iron in brain injury after intraventricular hemorrhage. Stroke.

[R36] Chen ZY, Yang YL, Li M, Gao L, Qu WM, Huang ZL, Yuan XS (2023). Whole-brain neural connectivity to cholinergic neurons in the nucleus basalis of Meynert. J Neurochem.

[R37] Chersi F, Burgess N (2015). The cognitive architecture of spatial navigation: hippocampal and striatal contributions. Neuron.

[R38] Chi NF, Chao SP, Huang LK, Chan L, Chen YR, Chiou HY, Hu CJ (2019). Plasma amyloid beta and tau levels are predictors of post-stroke cognitive impairment: a longitudinal study. Front Neurol.

[R39] Chou YH, Sundman M, Ton That V, Green J, Trapani C (2022). Cortical excitability and plasticity in Alzheimer’s disease and mild cognitive impairment: a systematic review and meta-analysis of transcranial magnetic stimulation studies. Ageing Res Rev.

[R40] Chouchani ET (2014). Ischaemic accumulation of succinate controls reperfusion injury through mitochondrial ROS. Nature.

[R41] Clark RE (2018). Current topics regarding the function of the medial temporal lobe memory system. Curr Top Behav Neurosci.

[R42] Cohen SJ, Stackman RW (2015). Assessing rodent hippocampal involvement in the novel object recognition task. A review. Behav Brain Res.

[R43] Cope EC, Wang SH, Waters RC, Gore IR, Vasquez B, Laham BJ, Gould E (2023). Activation of the CA2-ventral CA1 pathway reverses social discrimination dysfunction in Shank3B knockout mice. Nat Commun.

[R44] Culham JC, Kanwisher NG (2001). Neuroimaging of cognitive functions in human parietal cortex. Curr Opin Neurobiol.

[R45] Daniel A, Koumans H, Ganti L (2021). Impact of music therapy on gait after stroke. Cureus.

[R46] de Oliveira TCG, Carvalho-Paulo D, de Lima CM, de Oliveira RB, Santos Filho C, Diniz DG, Bento Torres Neto J, Picanço-Diniz CW (2020). Long-term environmental enrichment reduces microglia morphological diversity of the molecular layer of dentate gyrus. Eur J Neurosci.

[R47] del Zoppo GJ (2010). The neurovascular unit in the setting of stroke. J Intern Med.

[R48] Delattre C, Bournonville C, Auger F, Lopes R, Delmaire C, Henon H, Mendyk AM, Bombois S, Devedjian JC, Leys D, Cordonnier C, Bordet R, Bastide M (2018). Hippocampal deformations and entorhinal cortex atrophy as an anatomical signature of long-term cognitive impairment: from the MCAO rat model to the stroke patient. Transl Stroke Res.

[R49] Deller T, Adelmann G, Nitsch R, Frotscher M (1996). The alvear pathway of the rat hippocampus. Cell Tissue Res.

[R50] Deng YH, Dong LL, Zhang YJ, Zhao XM, He HY (2021). Enriched environment boosts the post-stroke recovery of neurological function by promoting autophagy. Neural Regen Res.

[R51] Domingues HS, Portugal CC, Socodato R, Relvas JB (2016). Oligodendrocyte, astrocyte, and microglia crosstalk in myelin development, damage, and repair. Front Cell Dev Biol.

[R52] Dong D, Ren A, Yang Y, Su J, Liu L, Zhuo W, Liang Y (2022). VX-765 alleviates β-amyloid deposition and secondary degeneration in the ipsilateral hippocampus and ameliorates cognitive decline after focal cortical infarction in rats. J Mol Neurosci.

[R53] Drummond E, Kavanagh T, Pires G, Marta-Ariza M, Kanshin E, Nayak S, Faustin A, Berdah V, Ueberheide B, Wisniewski T (2022). The amyloid plaque proteome in early onset Alzheimer’s disease and down syndrome. Acta Neuropathol Commun.

[R54] Du B, Liang M, Zheng H, Fan C, Zhang H, Lu X, Du Z, Lian Y, Zhang Y, Bi X (2020). Anti-mouse CX3CR1 antibody alleviates cognitive impairment, neuronal loss and myelin deficits in an animal model of brain ischemia. Neuroscience.

[R55] Du SQ, Wang XR, Xiao LY, Tu JF, Zhu W, He T, Liu CZ (2017). Molecular mechanisms of vascular dementia: what can be learned from animal models of chronic cerebral hypoperfusion?. Molecular Neurobiology.

[R56] Eichenbaum H (2017). Prefrontal-hippocampal interactions in episodic memory. Nat Rev Neurosci.

[R57] Ekstrom AD, Ranganath C (2018). Space, time, and episodic memory: the hippocampus is all over the cognitive map. Hippocampus.

[R58] El Amki M, Clavier T, Perzo N, Bernard R, Guichet PO, Castel H (2015). Hypothalamic, thalamic and hippocampal lesions in the mouse MCAO model: potential involvement of deep cerebral arteries?. J Neurosci Methods.

[R59] El Husseini N, Katzan IL, Rost NS, Blake ML, Byun E, Pendlebury ST, Aparicio HJ, Marquine MJ, Gottesman RF, Smith EE (2023). Cognitive impairment after ischemic and hemorrhagic stroke: a scientific statement from the American Heart Association/American Stroke Association. Stroke.

[R60] Erdem A, Yaşargil G, Roth P (1993). Microsurgical anatomy of the hippocampal arteries. J Neurosurg.

[R61] Esneault E, Pacary E, Eddi D, Freret T, Tixier E, Toutain J, Touzani O, Schumann-Bard P, Petit E, Roussel S, Bernaudin M (2008). Combined therapeutic strategy using erythropoietin and mesenchymal stem cells potentiates neurogenesis after transient focal cerebral ischemia in rats. J Cereb Blood Flow Metab.

[R62] Fan J, Li X, Yu X, Liu Z, Jiang Y, Fang Y, Zong M, Suo C, Man Q, Xiong L (2023). Global burden, risk factor analysis, and prediction study of ischemic stroke, 1990–2030. Neurology.

[R63] Fan W, Zhang Y, Li X, Xu C (2021). S-oxiracetam facilitates cognitive restoration after ischemic stroke by activating α7nAChR and the PI3K-mediated pathway. Neurochem Res.

[R64] Fang J, Song F, Chang C, Yao M (2023). Intracerebral hemorrhage models and behavioral tests in rodents. Neuroscience.

[R65] Feng B, Jia S, Li L, Wang J, Zhou F, Gou X, Wang Q, Xiong L, Zeng Y, Zhong H (2023). TAT-LBD-Ngn2-improved cognitive functions after global cerebral ischemia by enhancing neurogenesis. Brain Behav.

[R66] Ferrari-Souza JP (2023). APOEε4 potentiates amyloid β effects on longitudinal tau pathology. Nat Aging.

[R67] Fluri F, Schuhmann MK, Kleinschnitz C (2015). Animal models of ischemic stroke and their application in clinical research. Drug Des Devel Ther.

[R68] Freedman DJ, Ibos G (2018). An integrative framework for sensory, motor, and cognitive functions of the posterior parietal cortex. Neuron.

[R69] Fricker M, Tolkovsky AM, Borutaite V, Coleman M, Brown GC (2018). Neuronal cell death. Physiol Rev.

[R70] Friedman NP, Robbins TW (2022). The role of prefrontal cortex in cognitive control and executive function. Neuropsychopharmacology.

[R71] Fu C, Wu Y, Liu S, Luo C, Lu Y, Liu M, Wang L, Zhang Y, Liu X (2022). Rehmannioside A improves cognitive impairment and alleviates ferroptosis via activating PI3K/AKT/Nrf2 and SLC7A11/GPX4 signaling pathway after ischemia. J Ethnopharmacol.

[R72] Fu HY, Cui Y, Li Q, Wang D, Li H, Yang L, Wang DJ, Zhou JW (2023). LAMP-2A ablation in hippocampal CA1 astrocytes confers cerebroprotection and ameliorates neuronal injury after global brain ischemia. Brain Pathol.

[R73] Gallagher M, Chiba AA (1996). The amygdala and emotion. Curr Opin Neurobiol.

[R74] Gangemi A, De Luca R, Fabio RA, Lauria P, Rifici C, Pollicino P, Marra A, Olivo A, Quartarone A, Calabrò RS (2023). Effects of virtual reality cognitive training on neuroplasticity: a quasi-randomized clinical trial in patients with stroke. Biomedicines.

[R75] Gao C, Cai Y, Zhang X, Huang H, Wang J, Wang Y, Tong X, Wang J, Wu J (2015). Ischemic preconditioning mediates neuroprotection against ischemia in mouse hippocampal CA1 neurons by inducing autophagy. PLoS One.

[R76] Gao L, Zhang Y, Sterling K, Song W (2022). Brain-derived neurotrophic factor in Alzheimer’s disease and its pharmaceutical potential. Transl Neurodegener.

[R77] Ge J, Huang Y, Zhang Y, Liu L, Gu T, Liu X, Yao L, Cai M, Sun J, Song J (2020). Metformin inhibits propofol-induced apoptosis of mouse hippocampal neurons HT-22 through downregulating Cav-1. Drug Des Devel Ther.

[R78] Geigenmüller JN, Tari AR, Wisloff U, Walker TL (2024). The relationship between adult hippocampal neurogenesis and cognitive impairment in Alzheimer’s disease. Alzheimers Dement.

[R79] Geiller T, Priestley JB, Losonczy A (2023). A local circuit-basis for spatial navigation and memory processes in hippocampal area CA1. Curr Opin Neurobiol.

[R80] Goetzen B, Sztamska E (1992). Comparative anatomy of the arterial vascularization of the hippocampus in man and in experimental animals (cat, rabbit and sheep). Neuropatol Pol.

[R81] Gong P, Jia HY, Li R, Ma Z, Si M, Qian C, Zhu FQ, Sheng-Yong L (2023). Downregulation of Nogo-B ameliorates cerebral ischemia/reperfusion injury in mice through regulating microglia polarization via TLR4/NF-kappaB pathway. Neurochem Int.

[R82] Gonzales MM, Garbarino VR, Pollet E, Palavicini JP, Kellogg DL, Kraig E, Orr ME (2022). Biological aging processes underlying cognitive decline and neurodegenerative disease. J Clin Invest.

[R83] Gooch J, Wilcock DM (2016). Animal models of vascular cognitive impairment and dementia (VCID). Cell Mol Neurobiol.

[R84] Green DR, Llambi F (2015). Cell death signaling. Cold Spring Harb Perspect Biol.

[R85] Grienberger C, Magee JC (2022). Entorhinal cortex directs learning-related changes in CA1 representations. Nature.

[R86] Gu C, Kang X, Chen X, Sun Y, Li X (2024). Intracerebroventricular infusion of secretoneurin inhibits neuronal NLRP3-Apoptosis pathway and preserves learning and memory after cerebral ischemia. Neurochem Int.

[R87] Gu Y, He M, Zhou X, Liu J, Hou N, Bin T, Zhang Y, Li T, Chen J (2016). Endogenous IL-6 of mesenchymal stem cell improves behavioral outcome of hypoxic-ischemic brain damage neonatal rats by supressing apoptosis in astrocyte. Sci Rep.

[R88] Guan Y, Cao YL, Liu JW, Liu LT, Zheng YJ, Ma XF, Zhai FG (2023). Ginsenoside Rg1 attenuates cerebral ischemia-reperfusion injury through inhibiting the inflammatory activation of microglia. Exp Cell Res.

[R89] Gupta R, Ambasta RK, Pravir K (2021). Autophagy and apoptosis cascade: which is more prominent in neuronal death?. Cell Mol Life Sci.

[R90] Gureev AP, Sadovnikova IS, Chernyshova EV, Tsvetkova AD, Babenkova PI, Nesterova VV, Krutskikh EP, Volodina DE, Samoylova NA, Andrianova NV, Silachev DN, Plotnikov EY (2024). Beta-hydroxybutyrate mitigates sensorimotor and cognitive impairments in a photothrombosis-induced ischemic stroke in mice. Int J Mol Sci.

[R91] Hadanny A, Rittblat M, Bitterman M, May-Raz I, Suzin G, Boussi-Gross R, Zemel Y, Bechor Y, Catalogna M, Efrati S (2020). Hyperbaric oxygen therapy improves neurocognitive functions of post-stroke patients - a retrospective analysis. Restor Neurol Neurosci.

[R92] Hainmueller T, Bartos M (2020). Dentate gyrus circuits for encoding, retrieval and discrimination of episodic memories. Nat Rev Neurosci.

[R93] Han B, Jiang W, Liu H, Wang J, Zheng K, Cui P, Feng Y, Dang C, Bu Y, Wang QM, Ju Z, Hao J (2020). Upregulation of neuronal PGC-1α ameliorates cognitive impairment induced by chronic cerebral hypoperfusion. Theranostics.

[R94] Han Y, Yuan M, Guo YS, Shen XY, Gao ZK, Bi X (2021). Mechanism of endoplasmic reticulum stress in cerebral ischemia. Front Cell Neurosci.

[R95] Han ZS (1996). Morphological heterogeneity of non-pyramidal neurons in the CA1 region of the rat hippocampus. Neurosci Res.

[R96] Hassan SI, Bigler S, Siegelbaum SA (2023). Social odor discrimination and its enhancement by associative learning in the hippocampal CA2 region. Neuron.

[R97] He J, Zhao C, Liu W, Huang J, Liang S, Chen L, Tao J (2018). Neurochemical changes in the hippocampus and prefrontal cortex associated with electroacupuncture for learning and memory impairment. Int J Mol Med.

[R98] Heminghyt E, Herrman H, Skogan AH, Konglund A, Egge A, Lossius M, Dietrichs E, Taubøll E (2022). Cognitive change after DBS in refractory epilepsy: a randomized-controlled trial. Acta Neurol Scand.

[R99] Henze DA, Urban NN, Barrionuevo G (2000). The multifarious hippocampal mossy fiber pathway: a review. Neuroscience.

[R100] Hernández CC, Burgos CF, Gajardo AH, Silva-Grecchi T, Gavilan J, Toledo JR, Fuentealba J (2017). Neuroprotective effects of erythropoietin on neurodegenerative and ischemic brain diseases: the role of erythropoietin receptor. Neural Regen Res.

[R101] Ho HY, Chen MD, Tsai CC, Chen HM (2022). Effects of computerized cognitive training on cognitive function, activity, and participation in individuals with stroke: a randomized controlled trial. NeuroRehabilitation.

[R102] Hong I, Kaang BK (2022). The complexity of ventral CA1 and its multiple functionalities. Genes Brain Behav.

[R103] Hort J, Duning T, Hoerr R (2023). Ginkgo biloba Extract EGb 761 in the treatment of patients with mild neurocognitive impairment: a systematic review. Neuropsychiatr Dis Treat.

[R104] Hossein Geranmayeh M, Farokhi-Sisakht F, Sadigh-Eteghad S, Rahbarghazi R, Mahmoudi J, Farhoudi M (2023). Simultaneous pericytes and M2 microglia transplantation improve cognitive function in mice model of mPFC ischemia. Neuroscience.

[R105] Hosseini SM, Gholami Pourbadie H, Naderi N, Sayyah M, Zibaii MI (2018). Photothrombotically induced unilateral selective hippocampal ischemia in rat. J Pharmacol Toxicol Methods.

[R106] Huang LK, Chao SP, Hu CJ, Chien LN, Chiou HY, Lo YC, Hsieh YC (2022). Plasma phosphorylated-tau181 is a predictor of post-stroke cognitive impairment: a longitudinal study. Front Aging Neurosci.

[R107] Huang M, Jiang X, Liang Y, Liu Q, Chen S, Guo Y (2017). Berberine improves cognitive impairment by promoting autophagic clearance and inhibiting production of β-amyloid in APP/tau/PS1 mouse model of Alzheimer’s disease. Exp Gerontol.

[R108] Huang S, Ren C, Luo Y, Ding Y, Ji X, Li S (2023). New insights into the roles of oligodendrocytes regulation in ischemic stroke recovery. Neurobiol Dis.

[R109] Huang X, Zhao X, Li B, Cai Y, Zhang S, Wan Q, Yu F (2022). Comparative efficacy of various exercise interventions on cognitive function in patients with mild cognitive impairment or dementia: a systematic review and network meta-analysis. J Sport Health Sci.

[R110] Hullinger R, Puglielli L (2017). Molecular and cellular aspects of age-related cognitive decline and Alzheimer’s disease. Behav Brain Res.

[R111] Ibrahim AA, Abdel Mageed SS, Safar MM, El-Yamany MF, Oraby MA (2023). MitoQ alleviates hippocampal damage after cerebral ischemia: the potential role of SIRT6 in regulating mitochondrial dysfunction and neuroinflammation. Life Sci.

[R112] Ikeda T, Kawabori M, Zheng Y, Yamaguchi S, Gotoh S, Nakahara Y, Yoshie E, Fujimura M (2024). Intranasal administration of mesenchymal stem cell-derived exosome alleviates hypoxic-ischemic brain injury. Pharmaceutics.

[R113] Iliff JJ, Wang M, Liao Y, Plogg BA, Peng W, Gundersen GA, Benveniste H, Vates GE, Deane R, Goldman SA, Nagelhus EA, Nedergaard M (2012). A paravascular pathway facilitates CSF flow through the brain parenchyma and the clearance of interstitial solutes, including amyloid β. Sci Transl Med.

[R114] Iliff JJ, Lee H, Yu M, Feng T, Logan J, Nedergaard M, Benveniste H (2013). Brain-wide pathway for waste clearance captured by contrast-enhanced MRI. J Clin Invest.

[R115] Ishikawa H, Shindo A, Mizutani A, Tomimoto H, Lo EH, Arai K (2023). A brief overview of a mouse model of cerebral hypoperfusion by bilateral carotid artery stenosis. J Cereb Blood Flow Metab.

[R116] Jackson RJ, Hyman BT, Serrano-Pozo A (2024). Multifaceted roles of APOE in Alzheimer disease. Nat Rev Neurol.

[R117] Jadhav P, Karande M, Sarkar A, Sahu S, Sarmah D, Datta A, Chaudhary A, Kalia K, Sharma A, Wang X, Bhattacharya P (2023). Glial cells response in stroke. Cell Mol Neurobiol.

[R118] Jaspers RM, Block F, Heim C, Sontag KH (1990). Spatial learning is affected by transient occlusion of common carotid arteries (2VO): comparison of behavioural and histopathological changes after ‘2VO’ and ‘four-vessel-occlusion’ in rats. Neurosci Lett.

[R119] Jeon Y, Kim B, Kim JE, Kim BR, Ban S, Jeong JH, Kwon O, Rhie SJ, Ahn CW, Kim JH, Jung SU, Park SH, Lyoo IK, Yoon S (2016). Effects of ganglioside on working memory and the default mode network in individuals with subjective cognitive impairment: a randomized controlled trial. Am J Chin Med.

[R120] Jeong N, Singer AC (2022). Learning from inhibition: functional roles of hippocampal CA1 inhibition in spatial learning and memory. Curr Opin Neurobiol.

[R121] Jia B, Xu Y, Zhu X (2025). Cognitive resilience in Alzheimer’s disease: mechanism and potential clinical intervention. Ageing Res Rev.

[R122] Jia J (2016). The effects of DL-3-n-butylphthalide in patients with vascular cognitive impairment without dementia caused by subcortical ischemic small vessel disease: a multicentre, randomized, double-blind, placebo-controlled trial. Alzheimers Dement.

[R123] Jiang C, Zou X, Zhu R, Shi Y, Wu Z, Zhao F, Chen L (2019). The correlation between accumulation of amyloid beta with enhanced neuroinflammation and cognitive impairment after intraventricular hemorrhage. J Neurosurg.

[R124] Jiang RQ, Li QQ, Sheng R (2023). Mitochondria associated ER membranes and cerebral ischemia: Molecular mechanisms and therapeutic strategies. Pharmacol Res.

[R125] Jin R, Liu L, Zhang S, Nanda A, Li G (2013). Role of inflammation and its mediators in acute ischemic stroke. J Cardiovasc Transl Res.

[R126] Kamel H, Iadecola C (2012). Brain-immune interactions and ischemic stroke: clinical implications. Arch Neurol.

[R127] Kang BS, Choi BY, Kho AR, Lee SH, Hong DK, Park MK, Lee SH, Lee CJ, Yang HW, Woo SY, Park SW, Kim DY, Park JB, Chung WS, Suh SW (2023). Effects of pyruvate kinase M2 (PKM2) Gene deletion on astrocyte-specific glycolysis and global cerebral ischemia-induced neuronal death. Antioxidants (Basel).

[R128] Kang SH, Kang M, Han JH, Lee ES, Lee KJ, Chung SJ, Suh SI, Koh SB, Eo JS, Kim CK, Oh K (2023). Independent effect of Aβ burden on cognitive impairment in patients with small subcortical infarction. Alzheimers Res Ther.

[R129] Kassraian P, Bigler SK, Gilly Suarez DM, Shrotri N, Barnett A, Lee HJ, Young WS, Siegelbaum SA (2024). The hippocampal CA2 region discriminates social threat from social safety. Nat Neurosci.

[R130] Khaksar S, Bigdeli M, Samiee A, Shirazi-Zand Z (2022). Antioxidant and anti-apoptotic effects of cannabidiol in model of ischemic stroke in rats. Brain Res Bull.

[R131] Khlif MS, Werden E, Bird LJ, Egorova-Brumley N, Brodtmann A (2022). Atrophy of ipsilesional hippocampal subfields vary over first year after ischemic stroke. J Magn Reson Imaging.

[R132] Kim H, Seo JS, Lee SY, Ha KT, Choi BT, Shin YI, Ju Yun Y, Shin HK (2020). AIM2 inflammasome contributes to brain injury and chronic post-stroke cognitive impairment in mice. Brain Behav Immun.

[R133] Kirino T, Tamura A, Sano K (1984). Delayed neuronal death in the rat hippocampus following transient forebrain ischemia. Acta Neuropathol.

[R134] Kishi T, Matsunaga S, Oya K, Nomura I, Ikuta T, Iwata N (2017). Memantine for Alzheimer’s disease: an updated systematic review and meta-analysis. J Alzheimers Dis.

[R135] Kisler K, Nelson AR, Montagne A, Zlokovic BV (2017). Cerebral blood flow regulation and neurovascular dysfunction in Alzheimer disease. Nat Rev Neurosci.

[R136] Kitagawa K, Matsumoto M, Yang G, Mabuchi T, Yagita Y, Hori M, Yanagihara T (1998). Cerebral ischemia after bilateral carotid artery occlusion and intraluminal suture occlusion in mice: evaluation of the patency of the posterior communicating artery. J Cereb Blood Flow Metab.

[R137] Kober SE, Schweiger D, Reichert JL, Neuper C, Wood G (2017). Upper alpha based neurofeedback training in chronic stroke: brain plasticity processes and cognitive effects. Appl Psychophysiol Biofeedback.

[R138] Kominami R, Sonomura T, Ito T, Shinohara H, Kishibe M, Uemura M, Honma S (2023). Three-dimensional anatomical structure formed by granule cell layer and pyramidal cell layer in human hippocampus. Anat Sci Int.

[R139] Kulbe JR, Mulcahy Levy JM, Coultrap SJ, Thorburn A, Bayer KU (2014). Excitotoxic glutamate insults block autophagic flux in hippocampal neurons. Brain Res.

[R140] Labat-gest V, Tomasi S (2013). Photothrombotic ischemia: a minimally invasive and reproducible photochemical cortical lesion model for mouse stroke studies. J Vis Exp.

[R141] Lana D, Ugolini F, Giovannini MG (2020). An overview on the differential interplay among neurons-astrocytes-microglia in CA1 and CA3 hippocampus in hypoxia/ischemia. Front Cell Neurosci.

[R142] Lanctôt KL, Lindsay MP, Smith EE, Sahlas DJ, Foley N, Gubitz G, Austin M, Ball K, Bhogal S, Blake T, Herrmann N, Hogan D, Khan A, Longman S, King A, Leonard C, Shoniker T, Taylor T, Teed M, de Jong A, Mountain A, Casaubon LK, Dowlatshahi D, Swartz RH (2020). Canadian stroke best practice recommendations: mood, cognition and fatigue following stroke, 6th edition update 2019. Int J Stroke.

[R143] Lang M, Colby S, Ashby-Padial C, Bapna M, Jaimes C, Rincon SP, Buch K (2024). An imaging review of the hippocampus and its common pathologies. J Neuroimaging.

[R144] Lavenex P, Banta Lavenex P (2013). Building hippocampal circuits to learn and remember: insights into the development of human memory. Behav Brain Res.

[R145] Laver KE, Lange B, George S, Deutsch JE, Saposnik G, Crotty M (2017). Virtual reality for stroke rehabilitation. Cochrane Database Syst Rev.

[R146] LeDoux JE (2000). Emotion circuits in the brain. Annu Rev Neurosci.

[R147] Lee JH, Kim JY, Noh S, Lee H, Lee SY, Mun JY, Park H, Chung WS (2021). Astrocytes phagocytose adult hippocampal synapses for circuit homeostasis. Nature.

[R148] Lee SH, Bolshakov VY, Shen J (2023). Presenilins regulate synaptic plasticity in the perforant pathways of the hippocampus. Mol Brain.

[R149] Lee SH, Jung EM (2024). Adverse effects of early-life stress: focus on the rodent neuroendocrine system. Neural Regen Res.

[R150] Lee TS, Goh SJ, Quek SY, Phillips R, Guan C, Cheung YB, Feng L, Teng SS, Wang CC, Chin ZY, Zhang H, Ng TP, Lee J, Keefe R, Krishnan KR (2013). A brain-computer interface based cognitive training system for healthy elderly: a randomized control pilot study for usability and preliminary efficacy. PLoS One.

[R151] Leszczyński M, Staudigl T (2016). Memory-guided attention in the anterior thalamus. Neurosci Biobehav Rev.

[R152] Levine DA, Galecki AT, Langa KM, Unverzagt FW, Kabeto MU, Giordani B, Wadley VG (2015). Trajectory of cognitive decline after incident stroke. JAMA.

[R153] Li A, Lv S, Yu Z, Zhang Y, Ma H, Zhao H, Piao H, Li S, Zhang N, Sun C (2010). Simvastatin attenuates hypomyelination induced by hypoxia-ischemia in neonatal rats. Neurol Res.

[R154] Li D, Bai X, Jiang Y, Cheng Y (2021). Butyrate alleviates PTZ-induced mitochondrial dysfunction, oxidative stress and neuron apoptosis in mice via Keap1/Nrf2/HO-1 pathway. Brain Res Bull.

[R155] Li H, Li Y, Liang W, Wei ZZ, Li X, Tian Y, Qiao S, Yang Y, Yang L, Wu D, Yin W, Liu H, Zhang W, Zhang Y, Wang Z (2022). The identification of PSEN1 p.Tyr159Ser mutation in a non-canonic early-onset Alzheimer’s disease family. Mol Cell Neurosci.

[R156] Li H, Zhang C, Zhou Y, Deng Y, Zheng X, Xue X (2023). Neurovascular protection of alisol A on cerebral ischemia mice through activating the AKT/GSK3β pathway. Aging (Albany NY).

[R157] Li J, Shen S, Shen H (2024). Heat-shock protein A12A attenuates oxygen-glucose deprivation/reoxygenation-induced human brain microvascular endothelial cell dysfunction via PGC-1α/SIRT3 pathway. Drug Dev Res.

[R158] Li MZ, Zhang Y, Zou HY, Wang YL, Cheng BY, Wang L, Zhang QX, Lei JF, Zhao H (2018). Xiaoshuan enteric-coated capsule alleviates cognitive impairment by enhancing hippocampal glucose metabolism, hemodynamics and neuroplasticity of rat with chronic cerebral hypoperfusion. Sci Rep.

[R159] Li N, Ren C, Li S, Yu W, Jin K, Ji X (2023). Remote ischemic conditioning alleviates chronic cerebral hypoperfusion-induced cognitive decline and synaptic dysfunction via the miR-218a-5p/SHANK2 pathway. Prog Neurobiol.

[R160] Li N, Wang H, Liu H, Zhu L, Lyu Z, Qiu J, Zhao T, Ren H, Huang L, Chen S, Hu X, Zhou L (2023). The effects and mechanisms of acupuncture for post-stroke cognitive impairment: progress and prospects. Front Neurosci.

[R161] Li PY, Roxin A (2023). Rapid memory encoding in a recurrent network model with behavioral time scale synaptic plasticity. PLoS Comput Biol.

[R162] Li S, Zhang X, Fang Q, Zhou J, Zhang M, Wang H, Chen Y, Xu B, Wu Y, Qian L, Xu Y (2017). Ginkgo biloba extract improved cognitive and neurological functions of acute ischaemic stroke: a randomised controlled trial. Stroke Vasc Neurol.

[R163] Li XN, Lin L, Li XW, Zhu Q, Xie ZY, Hu YZ, Long QS, Wei XB, Wen YQ, Zhang LY, Zhang QK, Jing YC, Wei XH, Li XS (2024). BSA-stabilized selenium nanoparticles ameliorate intracerebral hemorrhage’s-like pathology by inhibiting ferroptosis-mediated neurotoxicology via Nrf2/GPX4 axis activation. Redox Biol.

[R164] Li Y, Yang Y, Zhao Y, Zhang J, Liu B, Jiao S, Zhang X (2019). Astragaloside IV reduces neuronal apoptosis and parthanatos in ischemic injury by preserving mitochondrial hexokinase-II. Free Radical Biology and Medicine.

[R165] Li Y, Zhang J (2021). Animal models of stroke. Animal Model Exp Med.

[R166] Li Y, Tan L, Yang C, He L, Liu L, Deng B, Liu S, Guo J (2023). Distinctions between the Koizumi and Zea Longa methods for middle cerebral artery occlusion (MCAO) model: a systematic review and meta-analysis of rodent data. Sci Rep.

[R167] Li Z, Wang H, Xiao G, Du H, He S, Feng Y, Zhang B, Zhu Y (2021). Recovery of post-stroke cognitive and motor deficiencies by Shuxuening injection via regulating hippocampal BDNF-mediated neurotrophin/Trk signaling. Biomed Pharmacother.

[R168] Li ZY, Yang X, Wang JK, Yan XX, Liu F, Zuo YC (2024). MFGE8 promotes adult hippocampal neurogenesis in rats following experimental subarachnoid hemorrhage via modifying the integrin β3/Akt signaling pathway. Cell Death Discov.

[R169] Liddelow SA (2017). Neurotoxic reactive astrocytes are induced by activated microglia. Nature.

[R170] Lin YT, Seo J, Gao F, Feldman HM, Wen HL, Penney J, Cam HP, Gjoneska E, Raja WK, Cheng J, Rueda R, Kritskiy O, Abdurrob F, Peng Z, Milo B, Yu CJ, Elmsaouri S, Dey D, Ko T, Yankner BA, Tsai LH (2018). APOE4 causes widespread molecular and cellular alterations associated with alzheimer’s disease phenotypes in human iPSC-derived brain cell types. Neuron.

[R171] Liu L, Fang YQ, Xue ZF, He YP, Fang RM, Li L (2012). Beta-asarone attenuates ischemia-reperfusion-induced autophagy in rat brains via modulating JNK, p-JNK, Bcl-2 and Beclin 1. Eur J Pharmacol.

[R172] Liu X, Jia X (2024). Neuroprotection of stem cells against ischemic brain injury: from bench to clinic. Transl Stroke Res.

[R173] Liu Z, Li R, Jiang C, Zhao S, Li W, Tang X (2018). The neuroprotective effect of lithium chloride on cognitive impairment through glycogen synthase kinase-3β inhibition in intracerebral hemorrhage rats. Eur J Pharmacol.

[R174] Lopez-Rojas J, de Solis CA, Leroy F, Kandel ER, Siegelbaum SA (2022). A direct lateral entorhinal cortex to hippocampal CA2 circuit conveys social information required for social memory. Neuron.

[R175] Lu B, Nagappan G, Lu Y (2014). BDNF and synaptic plasticity, cognitive function, and dysfunction. Handb Exp Pharmacol.

[R176] Lu W, Wen J (2020). Neuroprotective roles of total flavones of Camellia on early brain injury andcognitive dysfunction following subarachnoid hemorrhage in rats. Metab Brain Dis.

[R177] Magee JC, Grienberger C (2020). Synaptic plasticity forms and functions. Annu Rev Neurosci.

[R178] Magid-Bernstein J, Girard R, Polster S, Srinath A, Romanos S, Awad IA, Sansing LH (2022). Cerebral hemorrhage: pathophysiology, treatment, and future directions. Circ Res.

[R179] Mao R, Zong N, Hu Y, Chen Y, Xu Y (2022). Neuronal death mechanisms and therapeutic strategy in ischemic stroke. Neurosci Bull.

[R180] Marchi S, Patergnani S, Missiroli S, Morciano G, Rimessi A, Wieckowski MR, Giorgi C, Pinton P (2018). Mitochondrial and endoplasmic reticulum calcium homeostasis and cell death. Cell Calcium.

[R181] Martin SJ, Grimwood PD, Morris RG (2000). Synaptic plasticity and memory: an evaluation of the hypothesis. Annu Rev Neurosci.

[R182] Maturana-Candelas A, Gómez C, Poza J, Rodríguez-González V, Pablo VG, Lopes AM, Pinto N, Hornero R (2021). Influence of PICALM and CLU risk variants on beta EEG activity in Alzheimer’s disease patients. Sci Rep.

[R183] McBean DE, Kelly PA (1998). Rodent models of global cerebral ischemia: a comparison of two-vessel occlusion and four-vessel occlusion. Gen Pharmacol.

[R184] Meisner OC, Nair A, Chang SWC (2022). Amygdala connectivity and implications for social cognition and disorders. Handb Clin Neurol.

[R185] Meng F, Cui J, Wang P, Wang J, Sun J, Li L (2024). The phenotype changes of astrocyte during different ischemia conditions. Brain Sci.

[R186] Merino-Serrais P, Plaza-Alonso S, Hellal F, Valero-Freitag S, Kastanauskaite A, Plesnila N, DeFelipe J (2024). Structural changes of CA1 pyramidal neurons after stroke in the contralesional hippocampus. Brain Pathol.

[R187] Mertens EJ, Leibner Y, Pie J, Galakhova AA, Waleboer F, Meijer J, Heistek TS, Wilbers R, Heyer D, Goriounova NA, Idema S, Verhoog MB, Kalmbach BE, Lee BR, Gwinn RP, Lein ES, Aronica E, Ting J, Mansvelder HD, Segev I, de Kock CPJ (2024). Morpho-electric diversity of human hippocampal CA1 pyramidal neurons. Cell Rep.

[R188] Miller AL, Evanson NK, Taylor JM (2024). Use of donepezil for neurocognitive recovery after brain injury in adult and pediatric populations: a scoping review. Neural Regen Res.

[R189] Miller EK, Cohen JD (2001). An integrative theory of prefrontal cortex function. Annu Rev Neurosci.

[R190] Mizushima N, Levine B (2020). Autophagy in human diseases. N Engl J Med.

[R191] Mok VCT, Cai Y, Markus HS (2024). Vascular cognitive impairment and dementia: Mechanisms, treatment, and future directions. Int J Stroke.

[R192] Moskowitz MA, Lo EH, Iadecola C (2010). The science of stroke: mechanisms in search of treatments. Neuron.

[R193] Niu XL, Jiang X, Xu GD, Zheng GM, Tang ZP, Yin N, Li XQ, Yang YY, Lv PY (2019). DL-3-n-butylphthalide alleviates vascular cognitive impairment by regulating endoplasmic reticulum stress and the Shh/Ptch1 signaling-pathway in rats. J Cell Physiol.

[R194] Nixon RA, Yang DS (2012). Autophagy and neuronal cell death in neurological disorders. Cold Spring Harb Perspect Biol.

[R195] Ois A, Cuadrado-Godia E, Solano A, Perich-Alsina X, Roquer J (2009). Acute ischemic stroke in anterior choroidal artery territory. J Neurol Sci.

[R196] Ojo OB, Amoo ZA, Saliu IO, Olaleye MT, Farombi EO, Akinmoladun AC (2019). Neurotherapeutic potential of kolaviron on neurotransmitter dysregulation, excitotoxicity, mitochondrial electron transport chain dysfunction and redox imbalance in 2-VO brain ischemia/reperfusion injury. Biomed Pharmacother.

[R197] Ong LK, Chow WZ, TeBay C, Kluge M, Pietrogrande G, Zalewska K, Crock P, Åberg ND, Bivard A, Johnson SJ, Walker FR, Nilsson M, Isgaard J (2018). Growth hormone improves cognitive function after experimental stroke. Stroke.

[R198] Ono SE, Mader-Joaquim MJ, de Carvalho Neto A, de Paola L, Dos Santos GR, Silvado CES (2021). Relationship between hippocampal subfields and verbal and visual memory function in Mesial Temporal Lobe Epilepsy patients. Epilepsy Res.

[R199] Ordy JM, Wengenack TM, Bialobok P, Coleman PD, Rodier P, Baggs RB, Dunlap WP, Kates B (1993). Selective vulnerability and early progression of hippocampal CA1 pyramidal cell degeneration and GFAP-positive astrocyte reactivity in the rat four-vessel occlusion model of transient global ischemia. Exp Neurol.

[R200] Ouyang F, Jiang Z, Chen X, Chen Y, Wei J, Xing S, Zhang J, Fan Y, Zeng J (2021). Is cerebral amyloid-β deposition related to post-stroke cognitive impairment?. Transl Stroke Res.

[R201] Pantoni L, del Ser T, Soglian AG, Amigoni S, Spadari G, Binelli D, Inzitari D (2005). Efficacy and safety of nimodipine in subcortical vascular dementia: a randomized placebo-controlled trial. Stroke.

[R202] Patabendige A, Singh A, Jenkins S, Sen J, Chen R (2021). Astrocyte activation in neurovascular damage and repair following ischaemic stroke. Int J Mol Sci.

[R203] Perosa V, Priester A, Ziegler G, Cardenas-Blanco A, Dobisch L, Spallazzi M, Assmann A, Maass A, Speck O, Oltmer J, Heinze HJ, Schreiber S, Düzel E (2020). Hippocampal vascular reserve associated with cognitive performance and hippocampal volume. Brain.

[R204] Potter T, Lioutas VA, Tano M, Pan A, Meeks J, Woo D, Seshadri S, Selim M, Vahidy F (2021). Cognitive impairment after intracerebral hemorrhage: a systematic review of current evidence and knowledge gaps. Front Neurol.

[R205] Pronier É, Morici JF, Girardeau G (2023). The role of the hippocampus in the consolidation of emotional memories during sleep. Trends Neurosci.

[R206] Puglisi CH, Ander BP, Peterson C, Keiter JA, Hull H, Hawk CW, Kalistratova VS, Izadi A, Gurkoff GG, Sharp FR, Waldau B (2023). Sustained ICP elevation is a driver of spatial memory deficits after intraventricular hemorrhage and leads to activation of distinct microglial signaling pathways. Transl Stroke Res.

[R207] Qin C, Zhou LQ, Ma XT, Hu ZW, Yang S, Chen M, Bosco DB, Wu LJ, Tian DS (2019). Dual functions of microglia in ischemic stroke. Neurosci Bull.

[R208] Qin C, Guo C, Li H, Mu R, Cheng M, Li H, Feng X, Zhang B, Li Y, Jin J, Zhao X, Zhang X (2025). Preliminary feasibility study on DTI to assess the early brain injury in germinal matrix-intraventricular hemorrhage rats. Sci Rep.

[R209] Qin S, Zhang Z, Zhao Y, Liu J, Qiu J, Gong Y, Fan W, Guo Y, Guo Y, Xu Z, Guo Y (2022). The impact of acupuncture on neuroplasticity after ischemic stroke: a literature review and perspectives. Front Cell Neurosci.

[R210] Qu M, Xing F, Xing N (2022). Mesenchymal stem cells for the treatment of cognitive impairment caused by neurological diseases. Biotechnol Lett.

[R211] Ramsaran AI, Wang Y, Golbabaei A, Aleshin S, de Snoo ML, Yeung BA, Rashid AJ, Awasthi A, Lau J, Tran LM, Ko SY, Abegg A, Duan LC, McKenzie C, Gallucci J, Ahmed M, Kaushik R, Dityatev A, Josselyn SA, Frankland PW (2023). A shift in the mechanisms controlling hippocampal engram formation during brain maturation. Science.

[R212] Rangel-Gomez M, Alberini CM, Deneen B, Drummond GT, Manninen T, Sur M, Vicentic A (2024). Neuron-glial interactions: implications for plasticity, behavior, and cognition. J Neurosci.

[R213] Ranjbar Taklimie F, Gasterich N, Scheld M, Weiskirchen R, Beyer C, Clarner T, Zendedel A (2019). Hypoxia induces astrocyte-derived lipocalin-2 in ischemic stroke. Int J Mol Sci.

[R214] Ravi R, Das S, Hakami T, B MP, Pushparajan L (2025). Pharmacotherapy for poststroke cognitive impairment and poststroke cognitive impairment with dementia: a review. Stroke Res Treat.

[R215] Reisberg B, Doody R, Stöffler A, Schmitt F, Ferris S, Möbius HJ (2003). Memantine in moderate-to-severe Alzheimer’s disease. N Engl J Med.

[R216] Remondes M, Wilson MA (2013). Cingulate-hippocampus coherence and trajectory coding in a sequential choice task. Neuron.

[R217] Ren H, Kong Y, Liu Z, Zang D, Yang X, Wood K, Li M, Liu Q (2018). Selective NLRP3 (Pyrin domain-containing protein 3) inflammasome inhibitor reduces brain injury after intracerebral hemorrhage. Stroke.

[R218] Reyes-Soto G, Pérez-Cruz JC, Delgado-Reyes L, Castillo-Rangel C, Cacho Diaz B, Chmutin G, Nurmukhametov R, Sufianova G, Sufianov A, Nikolenko V, Sufianov R, Goncharov E, Montemurro N, Encarnacion Ramirez MJ (2024). The vertebrobasilar trunk and its anatomical variants: a microsurgical anatomical study. Diagnostics (Basel).

[R219] Rolls ET, Wirth S (2018). Spatial representations in the primate hippocampus, and their functions in memory and navigation. Prog Neurobiol.

[R220] Rosen WG, Mohs RC, Davis KL (1984). A new rating scale for Alzheimer’s disease. Am J Psychiatry.

[R221] Rosenberg GA (2009). Matrix metalloproteinases and their multiple roles in neurodegenerative diseases. Lancet Neurol.

[R222] Rots ML, de Borst GJ, van der Toorn A, Moll FL, Pennekamp CWA, Dijkhuizen RM, Bleys R (2019). Effect of bilateral carotid occlusion on cerebral hemodynamics and perivascular innervation: an experimental rat model. J Comp Neurol.

[R223] Roy DS, Kitamura T, Okuyama T, Ogawa SK, Sun C, Obata Y, Yoshiki A, Tonegawa S (2017). Distinct neural circuits for the formation and retrieval of episodic memories. Cell.

[R224] Safari S, Ahmadi N, Mohammadkhani R, Ghahremani R, Khajvand-Abedeni M, Shahidi S, Komaki A, Salehi I, Karimi SA (2021). Sex differences in spatial learning and memory and hippocampal long-term potentiation at perforant pathway-dentate gyrus (PP-DG) synapses in Wistar rats. Behav Brain Funct.

[R225] Sammons RP, Vezir M, Moreno-Velasquez L, Cano G, Orlando M, Sievers M, Grasso E, Metodieva VD, Kempter R, Schmidt H, Schmitz D (2024). Structure and function of the hippocampal CA3 module. Proc Natl Acad Sci U S A.

[R226] Sano R, Reed JC (2013). ER stress-induced cell death mechanisms. Biochimica et Biophysica Acta.

[R227] Santello M, Toni N, Volterra A (2019). Astrocyte function from information processing to cognition and cognitive impairment. Nat Neurosci.

[R228] Sarkala HB, Jahanshahi M, Dolatabadi LK, Namavar MR (2023). G-CSF improved the memory and dendritic morphology impairments in the hippocampal CA1 pyramidal neurons after brain ischemia in the male rats. Metab Brain Dis.

[R229] Schaapsmeerders P, van Uden IW, Tuladhar AM, Maaijwee NA, van Dijk EJ, Rutten-Jacobs LC, Arntz RM, Schoonderwaldt HC, Dorresteijn LD, de Leeuw FE, Kessels RP (2015). Ipsilateral hippocampal atrophy is associated with long-term memory dysfunction after ischemic stroke in young adults. Hum Brain Mapp.

[R230] Sevigny J (2024). Progranulin AAV gene therapy for frontotemporal dementia: translational studies and phase 1/2 trial interim results. Nat Med.

[R231] Shanbhag NC, Henning RH, Schilling L (2016). Long-term survival in permanent middle cerebral artery occlusion: a model of malignant stroke in rats. Sci Rep.

[R232] Sheintuch L, Geva N, Deitch D, Rubin A, Ziv Y (2023). Organization of hippocampal CA3 into correlated cell assemblies supports a stable spatial code. Cell Rep.

[R233] Sheng R, Chen C, Chen H, Yu P (2023). Repetitive transcranial magnetic stimulation for stroke rehabilitation: insights into the molecular and cellular mechanisms of neuroinflammation. Front Immunol.

[R234] Shi E, Shi K, Qiu S, Sheth KN, Lawton MT, Ducruet AF (2019). Chronic inflammation, cognitive impairment, and distal brain region alteration following intracerebral hemorrhage. FASEB J.

[R235] Shi Y, Yan D, Nan C, Sun Z, Zhuo Y, Huo H, Jin Q, Yan H, Zhao Z (2024). Salvianolic acid A inhibits ferroptosis and protects against intracerebral hemorrhage. Sci Rep.

[R236] Shin MC, Lee TK, Lee JC, Kim HI, Park CW, Cho JH, Kim DW, Ahn JH, Won MH, Lee CH (2022). Therapeutic effects of stiripentol against ischemia-reperfusion injury in gerbils focusing on cognitive deficit, neuronal death, astrocyte damage and blood brain barrier leakage in the hippocampus. Korean J Physiol Pharmacol.

[R237] Simpson DSA, Oliver PL (2020). ROS generation in microglia: understanding oxidative stress and inflammation in neurodegenerative disease. Antioxidants (Basel).

[R238] Smith MA, Ellis-Davies GC, Magee JC (2003). Mechanism of the distance-dependent scaling of Schaffer collateral synapses in rat CA1 pyramidal neurons. J Physiol.

[R239] Soltesz I, Losonczy A (2018). CA1 pyramidal cell diversity enabling parallel information processing in the hippocampus. Nat Neurosci.

[R240] Sommer CJ (2017). Ischemic stroke: experimental models and reality. Acta Neuropathol.

[R241] Song K, Liu X, Zheng Q, Zhang L, Zhang H, Yu H, Zhu Y, Huang LA, Chen Y (2020). Secondary injury to distal regions after intracerebral hemorrhage influence neurological functional outcome. Aging (Albany NY).

[R242] Song S, Yu L, Hasan MN, Paruchuri SS, Mullett SJ, Sullivan MLG, Fiesler VM, Young CB, Stolz DB, Wendell SG, Sun D (2022). Elevated microglial oxidative phosphorylation and phagocytosis stimulate post-stroke brain remodeling and cognitive function recovery in mice. Commun Biol.

[R243] Squire LR, Zola-Morgan S (1991). The medial temporal lobe memory system. Science.

[R244] Steffenach HA, Sloviter RS, Moser EI, Moser MB (2002). Impaired retention of spatial memory after transection of longitudinally oriented axons of hippocampal CA3 pyramidal cells. Proc Natl Acad Sci U S A.

[R245] Sun D, Daniels TE, Rolfe A, Waters M, Hamm R (2015). Inhibition of injury-induced cell proliferation in the dentate gyrus of the hippocampus impairs spontaneous cognitive recovery after traumatic brain injury. J Neurotrauma.

[R246] Sun JH, Tan L, Yu JT (2014). Post-stroke cognitive impairment: epidemiology, mechanisms and management. Ann Transl Med.

[R247] Sun L, Zhang H, Wang W, Chen Z, Wang S, Li J, Li G, Gao C, Sun X (2020). Astragaloside IV exerts cognitive benefits and promotes hippocampal neurogenesis in stroke mice by downregulating interleukin-17 expression via wnt pathway. Front Pharmacol.

[R248] Sun S, Zhou J, Li Z, Wu Y, Wang H, Zheng Q, Adu-Nti F, Fan J, Tian Y (2022). Progranulin promotes hippocampal neurogenesis and alleviates anxiety-like behavior and cognitive impairment in adult mice subjected to cerebral ischemia. CNS Neurosci Ther.

[R249] Sun W, Chen Y, Zhang Y, Geng Y, Tang X, Guo R, Zhang Z, Xu H, Tian X (2021). A modified four vessel occlusion model of global cerebral ischemia in rats. J Neurosci Methods.

[R250] Sun Z, Sun L, Tu L (2020). GABAB receptor-mediated PI3K/Akt signaling pathway alleviates oxidative stress and neuronal cell injury in a rat model of Alzheimer’s disease. J Alzheimers Dis.

[R251] Sweeney MD, Sagare AP, Zlokovic BV (2018). Blood-brain barrier breakdown in Alzheimer disease and other neurodegenerative disorders. Nat Rev Neurol.

[R252] Tang EY, Amiesimaka O, Harrison SL, Green E, Price C, Robinson L, Siervo M, Stephan BC (2018). Longitudinal effect of stroke on cognition: a systematic review. J Am Heart Assoc.

[R253] Tang W, Shin JD, Jadhav SP (2023). Geometric transformation of cognitive maps for generalization across hippocampal-prefrontal circuits. Cell Rep.

[R254] Tang X, Wang C, Xia L, Zhu W, Zhao L, Zhu W (2012). Volumetric MRI and 1H MRS study of hippocampus in unilateral MCAO patients: relationship between hippocampal secondary damage and cognitive disorder following stroke. Eur J Radiol.

[R255] Tanninen SE, Nouriziabari B, Morrissey MD, Bakir R, Dayton RD, Klein RL, Takehara-Nishiuchi K (2017). Entorhinal tau pathology disrupts hippocampal-prefrontal oscillatory coupling during associative learning. Neurobiol Aging.

[R256] Torres-López C, Cuartero MI, García-Culebras A, de la Parra J, Fernández-Valle ME, Benito M, Vázquez-Reyes S, Jareño-Flores T, de Castro-Millán FJ, Hurtado O, Buckwalter MS, García-Segura JM, Lizasoain I, Moro MA (2023). Ipsilesional hippocampal GABA is elevated and correlates with cognitive impairment and maladaptive neurogenesis after cortical stroke in mice. Stroke.

[R257] Tu WJ, Wang LD (2023). China stroke surveillance report 2021. Mil Med Res.

[R258] Tuo QZ, Zou JJ, Lei P (2021). Rodent models of vascular cognitive impairment. J Mol Neurosci.

[R259] U KP, Gao L, Zhang H, Ji Z, Lin J, Peng S, Zhang X, Xue S, Qin W, Tsang LL, Kong Y, Xia Y, Tang PM, Wang T, Lee WYW, Li G, Jiang X (2025). KDM3A controls postnatal hippocampal neurogenesis via dual regulation of the Wnt/β-catenin signaling pathway. Cell Death Differ.

[R260] Uchida H, Fujita Y, Matsueda M, Umeda M, Matsuda S, Kato H, Kasahara J, Araki T (2010). Damage to neurons and oligodendrocytes in the hippocampal CA1 sector after transient focal ischemia in rats. Cell Mol Neurobiol.

[R261] Uzdensky AB (2018). Photothrombotic stroke as a model of ischemic stroke. Transl Stroke Res.

[R262] van de Ven V, Lee C, Lifanov J, Kochs S, Jansma H, De Weerd P (2020). Hippocampal-striatal functional connectivity supports processing of temporal expectations from associative memory. Hippocampus.

[R263] van Dyck CH, Swanson CJ, Aisen P, Bateman RJ, Chen C, Gee M, Kanekiyo M, Li D, Reyderman L, Cohen S, Froelich L, Katayama S, Sabbagh M, Vellas B, Watson D, Dhadda S, Irizarry M, Kramer LD, Iwatsubo T (2023). Lecanemab in early Alzheimer’s disease. N Engl J Med.

[R264] Venna VR, Verma R, O’Keefe LM, Xu Y, Crapser J, Friedler B, McCullough LD (2014). Inhibition of mitochondrial p53 abolishes the detrimental effects of social isolation on ischemic brain injury. Stroke.

[R265] Villar-Conde S, Astillero-Lopez V, Gonzalez-Rodriguez M, Villanueva-Anguita P, Saiz-Sanchez D, Martinez-Marcos A, Flores-Cuadrado A, Ubeda-Bañon I (2021). The human hippocampus in Parkinson’s disease: an integrative stereological and proteomic study. J Parkinsons Dis.

[R266] Wang F, Liang W, Lei C, Kinden R, Sang H, Xie Y, Huang Y, Qu Y, Xiong L (2015). Combination of HBO and memantine in focal cerebral ischemia: is there a synergistic effect?. Mol Neurobiol.

[R267] Wang F, Yang YJ, Yang N, Chen XJ, Huang NX, Zhang J, Wu Y, Liu Z, Gao X, Li T, Pan GQ, Liu SB, Li HL, Fancy SPJ, Xiao L, Chan JR, Mei F (2018). Enhancing oligodendrocyte myelination rescues synaptic loss and improves functional recovery after chronic hypoxia. Neuron.

[R268] Wang G, Tang X, Zhao F, Qin X, Wang F, Yang D, Zhu H, Chen X (2023). Total saponins from Trillium tschonoskii Maxim promote neurological recovery in model rats with post-stroke cognitive impairment. Front Pharmacol.

[R269] Wang J, Fu X, Jiang C, Yu L, Wang M, Han W, Liu L, Wang J (2014). Bone marrow mononuclear cell transplantation promotes therapeutic angiogenesis via upregulation of the VEGF-VEGFR2 signaling pathway in a rat model of vascular dementia. Behav Brain Res.

[R270] Wang S, Cheng H, Dai G, Wang X, Hua R, Liu X, Wang P, Chen G, Yue W, An Y (2013). Umbilical cord mesenchymal stem cell transplantation significantly improves neurological function in patients with sequelae of traumatic brain injury. Brain Res.

[R271] Wang X, Hu Y, Xu R (2024). The pathogenic mechanism of TAR DNA-binding protein 43 (TDP-43) in amyotrophic lateral sclerosis. Neural Regen Res.

[R272] Wang Y, Chen G, Yu X, Li Y, Zhang L, He Z, Zhang N, Yang X, Zhao Y, Li N, Qiu H (2016). Salvianolic acid B ameliorates cerebral ischemia/reperfusion injury through inhibiting TLR4/MyD88 signaling pathway. Inflammation.

[R273] Wang Y, Li Z, Zhao X, Wang D, Li H, Xian Y, Liu L, Wang Y (2017). Stroke care quality in China: Substantial improvement, and a huge challenge and opportunity. Int J Stroke.

[R274] Wang Y, Li S, Pan Y, Wang M, Liao X, Shi J, Wang Y (2021). The effects of blood pressure on post stroke cognitive impairment: BP and PSCI. J Clin Hypertens (Greenwich).

[R275] Wang Y, Fu AKY, Ip NY (2022). Instructive roles of astrocytes in hippocampal synaptic plasticity: neuronal activity-dependent regulatory mechanisms. FEBS J.

[R276] Wang Y, Leak RK, Cao G (2022). Microglia-mediated neuroinflammation and neuroplasticity after stroke. Front Cell Neurosci.

[R277] Wang Y, Wang Z, Guo S, Li Q, Kong Y, Sui A, Ma J, Lu L, Zhao J, Li S (2024). SVHRSP alleviates age-related cognitive deficiency by reducing oxidative stress and neuroinflammation. Antioxidants (Basel).

[R278] Wang Z, Zhou F, Dou Y, Tian X, Liu C, Li H, Shen H, Chen G (2018). Melatonin alleviates intracerebral hemorrhage-induced secondary brain injury in rats via suppressing apoptosis, inflammation, oxidative stress, DNA damage, and mitochondria injury. Transl Stroke Res.

[R279] Washida K, Hattori Y, Ihara M (2019). Animal models of chronic cerebral hypoperfusion: from mouse to primate. Int J Mol Sci.

[R280] Wen X, Qi D, Sun Y, Huang X, Zhang F, Wu J, Fu Y, Ma K, Du Y, Dong H, Liu Y, Liu H, Song Y (2014). H₂S attenuates cognitive deficits through Akt1/JNK3 signaling pathway in ischemic stroke. Behav Brain Res.

[R281] Wolman DN, Moraff AM, Heit JJ (2022). Anatomy of the intracranial arteries: the internal carotid artery. Neuroimaging Clin N Am.

[R282] Wong FCC, Yatawara C, Low A, Foo H, Wong BYX, Lim L, Wang B, Kumar D, Ng KP, Kandiah N (2021). Cerebral small vessel disease influences hippocampal subfield atrophy in mild cognitive impairment. Transl Stroke Res.

[R283] Xia M, Gao C, Wang H, Shang J, Liu R, You Y, Zang W, Zhang J (2022). Novel PSEN1 (P284S) mutation causes alzheimer’s disease with cerebellar amyloid β-protein deposition. Curr Alzheimer Res.

[R284] Xia Q, Zhan G, Mao M, Zhao Y, Li X (2022). TRIM45 causes neuronal damage by aggravating microglia-mediated neuroinflammation upon cerebral ischemia and reperfusion injury. Exp Mol Med.

[R285] Xie J, Zhang Y, Li B, Xi W, Wang Y, Li L, Liu C, Shen L, Han B, Kong Y, Yao H, Zhang Z (2024). Inhibition of OGFOD1 by FG4592 confers neuroprotection by activating unfolded protein response and autophagy after ischemic stroke. J Transl Med.

[R286] Xie Y, Hou W, Song X, Yu Y, Huang J, Sun X, Kang R, Tang D (2016). Ferroptosis: process and function. Cell Death Differ.

[R287] Xin W, Chan JR (2020). Myelin plasticity: sculpting circuits in learning and memory. Nat Rev Neurosci.

[R288] Xing Y, Bai Y (2020). A review of exercise-induced neuroplasticity in ischemic stroke: pathology and mechanisms. Mol Neurobiol.

[R289] Xiong XY, Liu L, Yang QW (2016). Functions and mechanisms of microglia/macrophages in neuroinflammation and neurogenesis after stroke. Prog Neurobiol.

[R290] Xu G, Hao F, Zhao W, Qiu J, Zhao P, Zhang Q (2022). The influential factors and non-pharmacological interventions of cognitive impairment in children with ischemic stroke. Front Neurol.

[R291] Xu L, Gao Y, Hu M, Dong Y, Xu J, Zhang J, Lv P (2022). Edaravone dexborneol protects cerebral ischemia reperfusion injury through activating Nrf2/HO-1 signaling pathway in mice. Fundam Clin Pharmacol.

[R292] Xu P, Kong L, Tao C, Zhu Y, Cheng J, Li W, Shen N, Li R, Zhang C, Wang L, Zhang Y, Wang G, Liu X, Sun W, Hu W (2023). Elabela-APJ axis attenuates cerebral ischemia/reperfusion injury by inhibiting neuronal ferroptosis. Free Radic Biol Med.

[R293] Xu Q, Yin W, Zhou X, Wang S, Chen S, Yang J, Xi C, Sun Z (2024). Transcranial direct current stimulation for patients with walking difficulties caused by cerebral small vessel disease: a randomized controlled study. Front Aging Neurosci.

[R294] Xu R, Hu X, Jiang X, Zhang Y, Wang J, Zeng X (2020). Longitudinal volume changes of hippocampal subfields and cognitive decline in Parkinson’s disease. Quant Imaging Med Surg.

[R295] Xuan A, Long D, Li J, Ji W, Hong L, Zhang M, Zhang W (2012). Neuroprotective effects of valproic acid following transient global ischemia in rats. Life Sci.

[R296] Xue B, Huang J, Ma B, Yang B, Chang D, Liu J (2019). Astragaloside IV protects primary cerebral cortical neurons from oxygen and glucose deprivation/reoxygenation by activating the PKA/CREB pathway. Neuroscience.

[R297] Xue B, Ma B, Yao Y, Zhao A, Gao Y, Liu J (2022). BYHW decoction improves cognitive impairments in rats with cerebral microinfarcts via activation of the PKA/CREB pathway. Oxid Med Cell Longev.

[R298] Yamagata K (2021). Astrocyte-induced synapse formation and ischemic stroke. J Neurosci Res.

[R299] Yan N, Xu Z, Qu C, Zhang J (2021). Dimethyl fumarate improves cognitive deficits in chronic cerebral hypoperfusion rats by alleviating inflammation, oxidative stress, and ferroptosis via NRF2/ARE/NF-κB signal pathway. Int Immunopharmacol.

[R300] Yan W, Fan J, Zhang X, Song H, Wan R, Wang W, Yin Y (2021). Decreased neuronal synaptosome associated protein 29 contributes to poststroke cognitive impairment by disrupting presynaptic maintenance. Theranostics.

[R301] Yang LC, Li J, Xu SF, Cai J, Lei H, Liu DM, Zhang M, Rong XF, Cui DD, Wang L, Peng Y, Wang XL (2015). L-3-n-butylphthalide promotes neurogenesis and neuroplasticity in cerebral ischemic rats. CNS Neurosci Ther.

[R302] Yang Y, Kimura-Ohba S, Thompson J, Rosenberg GA (2016). Rodent models of vascular cognitive impairment. Transl Stroke Res.

[R303] Yang Y, Wang JZ (2017). From structure to behavior in basolateral amygdala-hippocampus circuits. Front Neural Circuits.

[R304] Yang Y, Song J, Liu N, Wei G, Liu S, Zhang S, Jiang N, Yang H, Du G (2022). Salvianolic acid A relieves cognitive disorder after chronic cerebral ischemia: involvement of Drd2/Cryab/NF-κB pathway. Pharmacol Res.

[R305] Yao D, Li T, Yu L, Hu M, He Y, Zhang R, Wu J, Li S, Kuang W, Yang X, Liu G, Xie Y (2024). Selective degradation of hyperphosphorylated tau by proteolysis-targeting chimeras ameliorates cognitive function in Alzheimer’s disease model mice. Front Pharmacol.

[R306] Yasuda K, Yamada S, Uenishi S, Ikeda N, Tamaki A, Ohoshi Y, Tsuji T, Takahashi S (2022). Hippocampal subfield volumes and cognitive function in schizophrenia and mood disorders. Neuropsychobiology.

[R307] Yeh TH, Hwang HM, Chen JJ, Wu T, Li AH, Wang HL (2005). Glutamate transporter function of rat hippocampal astrocytes is impaired following the global ischemia. Neurobiol Dis.

[R308] Yi Z, Liang W, Ruan W, Hua W, Lin X (2020). A meta-analysis of Au/polypropionic acid nanoparticles loaded with olacetam for the treatment of vascular cognitive impairment. J Nanosci Nanotechnol.

[R309] You W, Li Y, Liu K, Mi X, Li Y, Guo X, Li Z (2024). Latest assessment methods for mitochondrial homeostasis in cognitive diseases. Neural Regen Res.

[R310] Yu F, Vock DM, Zhang L, Salisbury D, Nelson NW, Chow LS, Smith G, Barclay TR, Dysken M, Wyman JF (2021). Cognitive effects of aerobic exercise in Alzheimer’s disease: a pilot randomized controlled trial. J Alzheimers Dis.

[R311] Yuan M, Zhang XX, Fu XC, Bi X (2020). Enriched environment alleviates post-stroke cognitive impairment through enhancing α7-nAChR expression in rats. Arq Neuropsiquiatr.

[R312] Zeng T, Liu J, Zhang W, Yu Y, Ye X, Huang Q, Li P, Jiang Q (2024). Update on the mechanism of microglia involvement in post-stroke cognitive impairment. Front Aging Neurosci.

[R313] Zera KA, Buckwalter MS (2020). The local and peripheral immune responses to stroke: implications for therapeutic development. Neurotherapeutics.

[R314] Zhang B, Chen X, Lv Y, Wu X, Gui L, Zhang Y, Qiu J, Song G, Yao W, Wan L, Zhang C (2019). Cdh1 overexpression improves emotion and cognitive-related behaviors via regulating hippocampal neuroplasticity in global cerebral ischemia rats. Neurochem Int.

[R315] Zhang J, Xiao Y, Liu H, Xu L, Guo X, Gao Y, Li M, Xu J, Qi Q, Lv P (2023). Edaravone dexborneol alleviates neuroinflammation by reducing neuroglial cell proliferation and suppresses neuronal apoptosis/autophagy in vascular dementia rats. Neurochem Res.

[R316] Zhang N, Gordon ML (2018). Clinical efficacy and safety of donepezil in the treatment of Alzheimer’s disease in Chinese patients. Clin Interv Aging.

[R317] Zhang Q, Jia M, Wang Y, Wang Q, Wu J (2022). Cell death mechanisms in cerebral ischemia-reperfusion injury. Neurochem Res.

[R318] Zhang Q, Liu Z, Li B, Mu L, Sheng K, Xiong Y, Cheng J, Zhou J, Xiong Z, Zhou L, Jiang L, Wu J, Cai X, Zheng Y, Du W, Li Y, Zhu Y (2024). Platinum-loaded cerium oxide capable of repairing neuronal homeostasis for cerebral ischemia-reperfusion injury therapy. Adv Healthc Mater.

[R319] Zhang X, Li H, Wang H, Zhang Q, Deng X, Zhang S, Wang L, Guo C, Zhao F, Yin Y, Zhou T, Zhong J, Feng H, Chen W, Zhang J, Feng H, Hu R (2024). Iron/ROS/Itga3 mediated accelerated depletion of hippocampal neural stem cell pool contributes to cognitive impairment after hemorrhagic stroke. Redox Biol.

[R320] Zhang X, Zhang Q, Zhang Q, Wang H, Yin Y, Li H, Huang Q, Guo C, Zhong J, Zhou T, Chen Y, Chen Z, Shan Q, Hu R (2024). Tetrahydrofolate attenuates cognitive impairment after hemorrhagic stroke by promoting hippocampal neurogenesis via PTEN signaling. eNeuro.

[R321] Zhang Y, Khan S, Liu Y, Wu G, Yong VW, Xue M (2022). Oxidative stress following intracerebral hemorrhage: from molecular mechanisms to therapeutic targets. Front Immunol.

[R322] Zhang Y, Shen L, Xie J, Li L, Xi W, Li B, Bai Y, Yao H, Zhang S, Han B (2023). Pushen capsule treatment promotes functional recovery after ischemic stroke. Phytomedicine.

[R323] Zhao K, Li GZ, Nie LY, Ye XM, Zhu GY (2022). Edaravone for acute ischemic stroke: a systematic review and meta-analysis. Clin Ther.

[R324] Zhao M, Gao J, Cui C, Zhang Y, Jiang X, Cui J (2021). Inhibition of PTEN ameliorates secondary hippocampal injury and cognitive deficits after intracerebral hemorrhage: involvement of AKT/FoxO3a/ATG-mediated autophagy. Oxid Med Cell Longev.

[R325] Zhao M, Gao J, Zhang Y, Jiang X, Tian Y, Zheng X, Wang K, Cui J (2021). Elevated miR-29a contributes to axonal outgrowth and neurological recovery after intracerebral hemorrhage via targeting PTEN/PI3K/Akt pathway. Cell Mol Neurobiol.

[R326] Zhao W, Luo C, Wang J, Gong J, Li B, Gong Y, Wang J, Wang H (2014). 3-N-butylphthalide improves neuronal morphology after chronic cerebral ischemia. Neural Regen Res.

[R327] Zhao Y, Wang J, Du J, Li B, Gou X, Liu J, Hou L, Sang H, Deng B (2018). TAT-Ngn2 enhances cognitive function recovery and regulates caspase-dependent and mitochondrial apoptotic pathways after experimental stroke. Front Cell Neurosci.

[R328] Zhao Y, Yang J, Li C, Zhou G, Wan H, Ding Z, Wan H, Zhou H (2020). Role of the neurovascular unit in the process of cerebral ischemic injury. Pharmacol Res.

[R329] Zhao Y, Zhang J, Zheng Y, Zhang Y, Zhang XJ, Wang H, Du Y, Guan J, Wang X, Fu J (2021). NAD(+) improves cognitive function and reduces neuroinflammation by ameliorating mitochondrial damage and decreasing ROS production in chronic cerebral hypoperfusion models through Sirt1/PGC-1α pathway. J Neuroinflammation.

[R330] Zhao Z, Cai H, Zheng W, Liu T, Sun D, Han G, Zhang Y, Wu D (2021). Atrophic pattern of hippocampal subfields in post-stroke demented patient. J Alzheimers Dis.

[R331] Zheng J, Fang Y, Zhang M, Gao Q, Li J, Yuan H, Jin W, Lin Z, Lin W (2024). Mechanisms of ferroptosis in hypoxic-ischemic brain damage in neonatal rats. Exp Neurol.

[R332] Zhou B, Wang H, Zhang B, Zhang L (2021). Licochalcone B attenuates neuronal injury through anti-oxidant effect and enhancement of Nrf2 pathway in MCAO rat model of stroke. Int Immunopharmacol.

[R333] Zhou L, Jin Y, Wu D, Cun Y, Zhang C, Peng Y, Chen N, Yang X, Zhang S, Ning R, Kuang P, Wang Z, Zhang P (2023). Current evidence, clinical applications, and future directions of transcranial magnetic stimulation as a treatment for ischemic stroke. Front Neurosci.

[R334] Zhu T, Wang L, Xie W, Meng X, Feng Y, Sun G, Sun X (2021). Notoginsenoside R1 improves cerebral ischemia/reperfusion injury by promoting neurogenesis via the BDNF/Akt/CREB pathway. Front Pharmacol.

